# An Engineered Solidified Peptide Hemocyte Sponge as Nanomotor Storage to Combat Bacterial Colitis

**DOI:** 10.1002/advs.202515620

**Published:** 2025-11-29

**Authors:** Yuxin Fang, Jianming Yan, Tongxin Yu, Zichuan An, Kaikai Lv, Chenyu Xue, Hongyang Yu, Na Dong, Anshan Shan

**Affiliations:** ^1^ Laboratory of Molecular Nutrition and Immunity College of Animal Science and Technology Northeast Agricultural University Harbin 150030 P. R. China; ^2^ Department of Radiation Oncology The Second Affiliated Hospital Harbin Medical University Harbin 150076 P. R. China

**Keywords:** bacterial infections, cellular engineering, endotoxin neutralization, immobilized antimicrobial peptides, nanomotors

## Abstract

The emergence of drug‐resistant bacteria has significantly heightened the urgency for effective interventions within the global public healthcare system. Furthermore, the structural diversity of endotoxins across different bacterial species poses a major limitation on the clearance efficiency of structure‐specific anti‐endotoxin antibodies. In this study, a peptide‐based material with membrane‐disrupting functionality is developed. The topological configuration of the peptide is immobilized through Ni^2+^ coordination, and the thrust force generated by a redox reaction is harnessed to facilitate deep tissue penetration and enhance resistance to oxidative degradation. Notably, it is discovered that the immobilized peptide‐based nanomotor storage system, coated with red blood cell membranes, can effectively mitigate colitis‐induced damage by concentrating and sequestering endotoxins while distributing the bacterial burden. This approach operates independently of molecular structure‐specific binding. Following endotoxin capture, the nanomotor storage system utilizes electrostatic interactions from immobilized peptide to adsorb endotoxins, thereby preventing excessive release of pro‐inflammatory cytokines during prolonged infection, ultimately enabling effective management of bacterial colitis. The top‐down fabrication of endotoxin storage materials presents a broadly applicable strategy against bacterial infections. The peptides immobilized via metal coordination offer an efficient pathway for bacterial and endotoxin capture, enabling the development of comprehensive storage systems.

## Graphical abstract

Peptides immobilized via nickel coordination can stabilize the framework structure. Following coating with red blood cell membranes, these constructs are capable of continuously sequestering endotoxins and mitigating colonic pathogenic damage by alleviating bacterial‐induced ferroptosis.

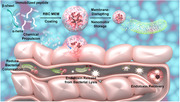

## 1 Introduction

Numerous human diseases are attributed to bacterial infections, including sepsis, bacterial dermatological infections, and a range of bacterial respiratory tract infections, the majority of which are currently treatable with antibiotics.^[^ ^1^, ^2^ ^]^ However, drug‐resistant bacterial strains have emerged and continue to evolve, presenting significant challenges to the effective use of antibiotic therapies.^[^ ^3^, ^4^ ^]^ Furthermore, conventional anti‐infective strategies have predominantly focused on bacterial eradication, frequently overlooking the importance of endotoxin clearance.^[^ ^5^ ^]^ Notably, endotoxins released by Gram‐negative bacteria during cellular growth, lysis, or antibiotic‐induced death can initiate inflammatory cascades and are primarily responsible for clinical manifestations such as fever, shock, organ dysfunction, and even mortality.^[^ ^6^, ^7^ ^]^ Consequently, the use of anti‐endotoxin antibodies and endotoxin‐neutralizing proteins to adsorb and neutralize circulating endotoxins represents a critical therapeutic approach. Accordingly, an increasing number of therapeutic agents are being developed to target both bacterial pathogens and endotoxins within and beyond infected cells.^[^ ^8^, ^9^, ^10^ ^]^ However, this growing complexity has reduced the binding affinity and clearance efficacy of currently available endotoxin‐neutralizing agents. Although several anti‐endotoxin therapeutics have advanced to clinical trials, the inflammatory response has not been effectively mitigated as anticipated.^[^ ^11^ ^]^

In our previous studies, numerous LPS‐neutralizing antimicrobial peptides have been developed, capable of sequestering endotoxins through electrostatic interactions and neutralizing multiple pro‐inflammatory cytokines for the treatment of bacterial infection‐induced peritonitis.^[^ ^12^, ^13^ ^]^ However, despite their antimicrobial activity being comparable to that of conventional antibiotics, the in vivo application of antimicrobial peptides has been consistently hindered by proteolytic degradation. Although various peptide motifs with resistance to high concentrations of digestive enzymes in vitro have been engineered, their effective use in gastrointestinal anti‐infective therapy remains limited. Moreover, the persistent disruption of the intestinal barrier caused by endotoxin release from Gram‐negative bacteria further exacerbates the burden on antimicrobial peptides to effectively clear endotoxins. Therefore, to achieve efficient elimination of both bacteria and endotoxins for the treatment of bacterial infections, it is essential to develop next‐generation biomimetic formulations that preserve the spatial conformation of peptide‐based therapeutics within the gastrointestinal tract, thereby enabling their biological activity. Recently, the erythrocyte membrane camouflaging strategy has emerged as a promising approach to overcome challenges such as systemic toxicity, off‐target effects, and limited therapeutic efficacy in complex pathological microenvironments, particularly in bionic nanomedicine platforms designed for detoxification. Red blood cell membranes have garnered significant attention due to their superior biocompatibility and prolonged circulation mediated by CD47.^[^ ^14^ ^]^ In particular, cell membranes have garnered significant attention as nano‐sponges, offering a promising and innovative therapeutic strategy. Owing to their capacity to broadly neutralize diverse biological threats, these nano‐sponges act as decoy cells and effectively sequester inflammatory mediators. This approach exhibits enhanced efficacy in comparison to monoclonal antibodies, which are limited to targeting specific pro‐inflammatory cytokines.^[^ ^15^, ^16^, ^17^ ^]^ For example, platelet membrane‐based biomimetic modifications allow PM@Pic/PtCD@NP to utilize neutrophils as carriers to reach inflammatory sites and release chrysin, thereby inhibiting neutrophil aggregation.^[^ ^18^ ^]^ Additionally, cell membrane coatings can preserve the topological structure of protein‐based therapeutics, thereby facilitating drug recycling within the gastrointestinal tract.

**Scheme 1:**
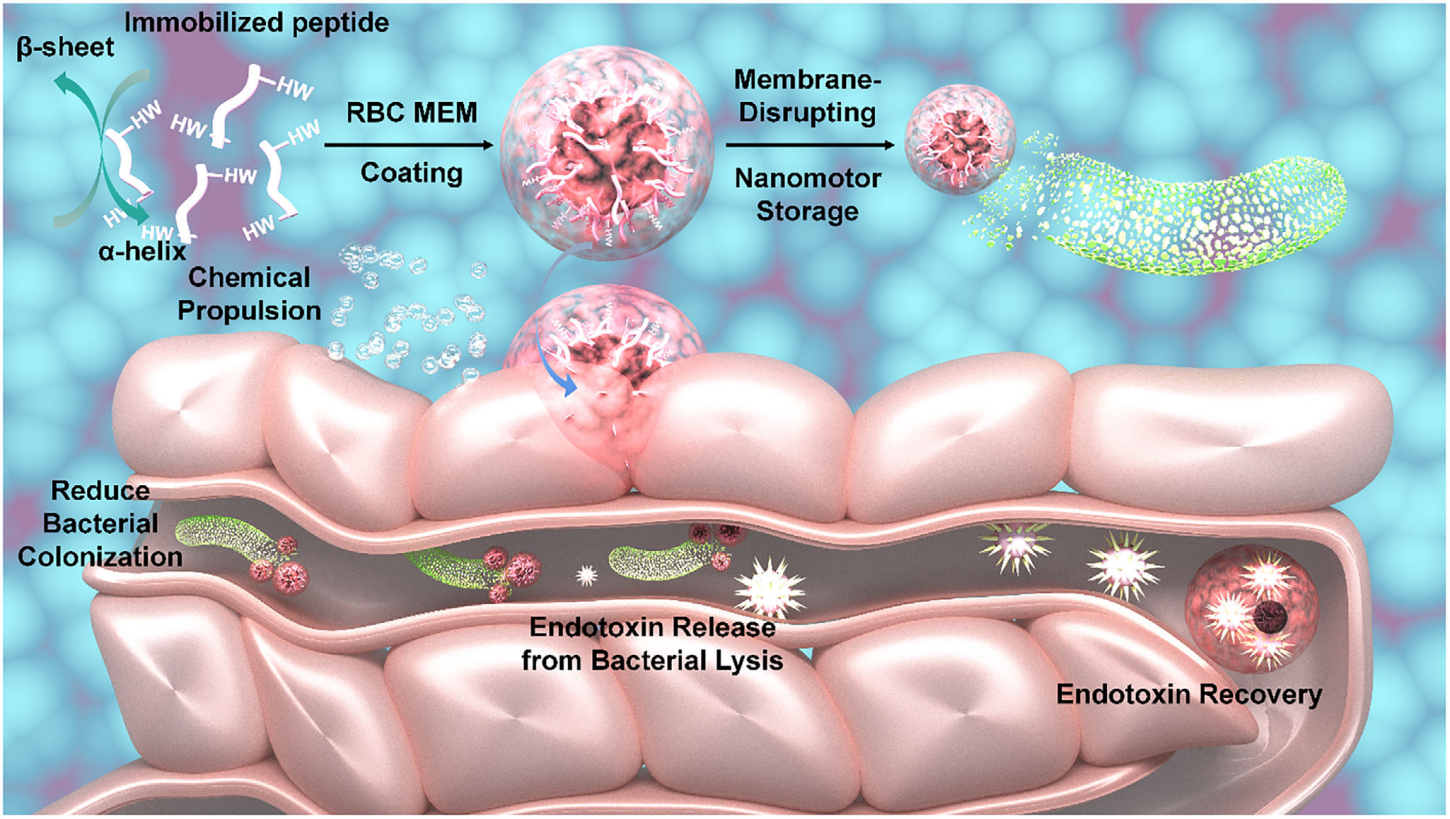
Schematic diagram of the formation and storage of endotoxin by nanomotor storage.

Antimicrobial peptides, as evolutionarily conserved components of the innate immune system, play a critical role in host defense against microbial pathogens. Owing to their broad‐spectrum antimicrobial activity and favorable biocompatibility, antimicrobial peptides exhibit cell‐selective properties comparable to those of conventional antibiotics, while demonstrating significantly lower propensity for inducing resistance. Consequently, they are considered among the most promising alternative therapeutic agents in the postantibiotic era. Moreover, the 3D topological architecture of antimicrobial peptides may serve as an ideal core scaffold for the effective capture of bacterial endotoxins. To address the challenge of neutralizing bacterial infections, we employed machine learning models to systematically screen for potent antimicrobial motifs and developed enzyme‐stable antimicrobial peptides by strategically avoiding proteolytic cleavage sites. Given the inherent flexibility and conformational variability of antimicrobial peptides, Ni^2+^ was utilized to immobilise their structural conformation, ensuring functional integrity under varying environmental conditions. Red blood cell membranes, functioning as biological decoys, shield the immobilized antimicrobial peptides from immune recognition. Notably, it was observed that the red blood cell membrane coating could serve as an endotoxin storage reservoir by concentrating and sequestering endotoxins, thereby preventing intestinal barrier disruption during prolonged infections. Mechanistic studies revealed that the membrane‐coated system significantly attenuated oxidative stress in an ETEC K88‐induced chronic colitis model, with therapeutic efficacy comparable to that of the clinically used antibiotic ciprofloxacin, highlighting its potential as an advanced biomimetic therapeutic platform (**Scheme** **1**).

## 2 Results and Discussion

### 2.1 Preparation, Characterizations, and Biological Potential of Biomimetic Peptides (BPs) Inspired by Deep Learning

In the livestock industry, peptide antibiotics have been extensively utilized as alternatives to conventional antibiotics for mitigating postweaning diarrhea in piglets caused by pathogenic microorganisms.^[^ ^19^, ^20^ ^]^ However, several challenges remain, including their low stability in systemic circulation, uncontrolled drug releases, and the consequent issues of drug residues, cytotoxicity toward normal cells, and the potential development of drug resistance due to overuse.^[^ ^21^, ^22^ ^]^ Therefore, novel strategies are required to enhance the performance of peptide antibiotics and to develop more effective antibacterial therapies. We propose that the introduction of coordination peptides with well‐defined structures, diverse geometric configurations, and topological architectures may address the key limitations of current peptide antibiotics, particularly in balancing nanoparticle size and cytotoxicity.^[^ ^23^ ^]^

**Figure 1:**
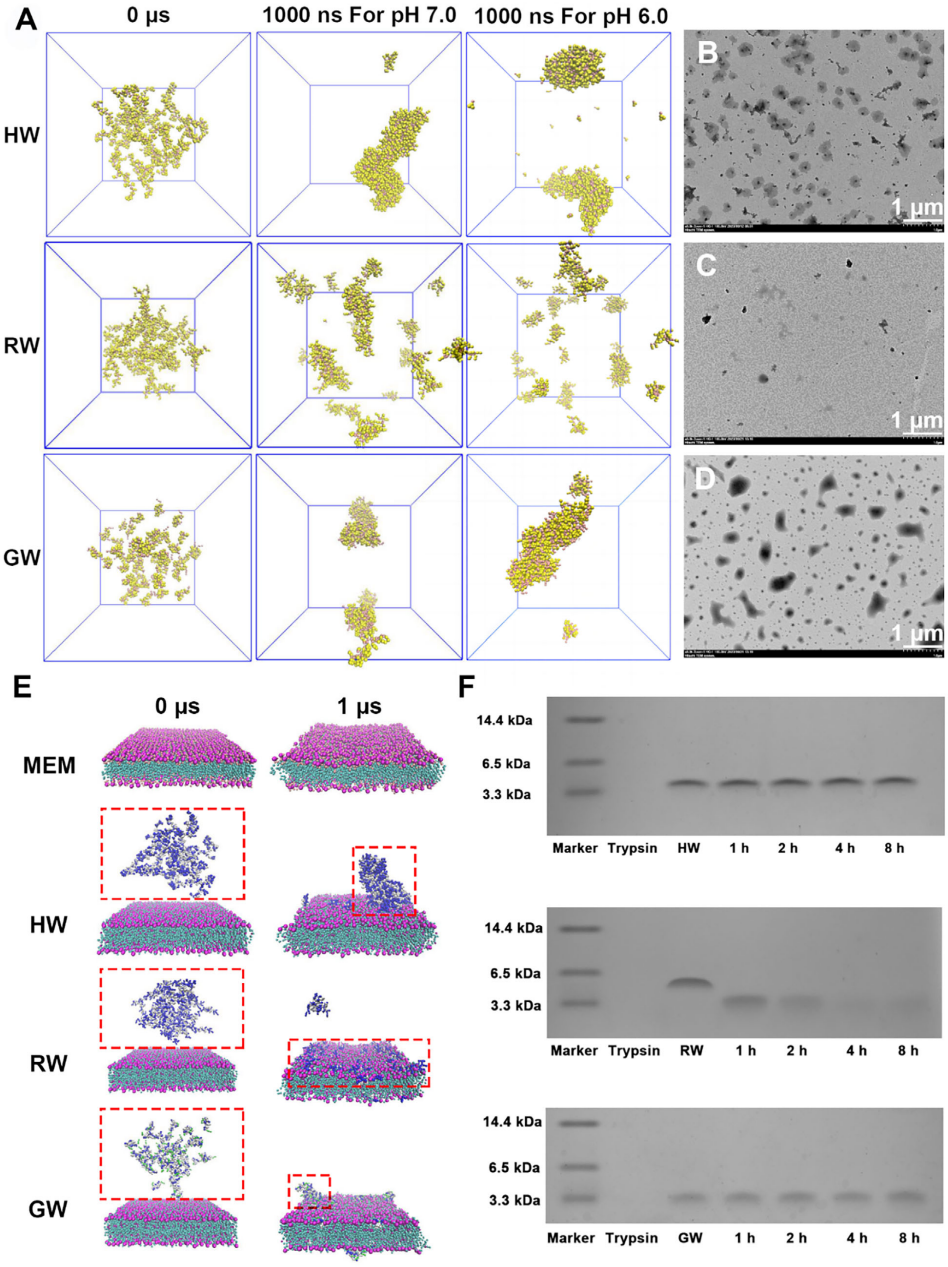
Structural prediction and characterization of BPs. A) Visualization of the BPs structure following a 1000 ns molecular dynamics simulation under varying pH conditions. The model exhibiting the highest structural confidence was selected and placed in the GROMACS simulation environment. Subsequently, 50 molecules were randomly introduced using CHARMM‐GUI and subjected to optimization. Prior to the optimization process, the MARTINI coarse‐grained (CG) force field was applied to enhance the spatiotemporal resolution of the system, which is typically limited in conventional all‐atom (AA) simulations. TEM showing the formation of diverse nanostructures by B) HW, C) RW, and D) GW in PBS solution at pH 7.4. E) Molecular dynamics simulation (1 µs) of the interaction between BPs and a simulated bacterial phospholipid bilayer, performed with the GROMACS program under the MARTINI force field. F) Tricine‐SDS‐PAGE analysis revealed the structural integrity of BPs post‐hydrolysis by trypsin. The peptide sequences and corresponding physicochemical parameters of BPs are presented in detail in Table S1 (Supporting Information).

First, based on a self‐constructed BPs database—comprising 460 samples meticulously collected by our laboratory under consistent experimental conditions over the past decade—188 highly selective BPs were identified. Drawing inspiration from naturally occurring enzyme inhibition sites, we proposed a stable structural framework composed of a Trp‐zipper dual‐domain motif and a proline‐rich hinge region.^[^ ^24^, ^25^ ^]^ This design incorporated a histidine‐containing tail chain to facilitate nonspecific recognition and preserve the motif's antiferroptotic properties,^[^ ^12^ ^]^ aiming to develop a peptide system that combines structural stability with broad‐spectrum bactericidal activity (Figures S1–S3 and Table S1, Supporting Information). High‐confidence structural models of these BPs were generated using I‐TASSER and SPICKER‐based structure prediction methods, followed by molecular dynamics simulations (Figures S4 and S5, Supporting Information). A 1 µs simulation revealed that HW and GW tended to form nanosheets or loosely aggregated structures due to hydrophobic and electrostatic interactions, whereas RW failed to achieve stable self‐assembly due to strong electrostatic repulsion (**Figure** **1**A). Multi‐dye fluorescence analysis was employed to investigate the self‐assembly behavior and determine the critical micelle concentration of BPs (Figures S6–S8, Supporting Information). Transmission electron microscopy (TEM) confirmed that HW could form well‐defined nanosheets at a concentration of 256 µm, which was consistent with the simulation results (Figure 1B–D). Dynamic light scattering analysis demonstrated that BPs maintained a stable particle size under neutral conditions (Figure S9, Supporting Information). Furthermore, BPs retain a β‐sheet secondary structure across diverse environmental conditions (Figure S10, Supporting Information).

Based on full‐scale antimicrobial evaluation against multiple pathogens (Gram‐positive/+− bacteria and fungi), the BPs exhibited excellent MIC/GM values, with their potency originating from a membrane disruption mechanism enhanced by Trp‐zipper and cation‐π synergistic interactions (Figure S11 and Tables S2–S4, Supporting Information). To further clarify the interaction between BPs and bacterial membranes, we introduced the peptides into a large unilamellar vesicle (LUV) system composed of 1‐palmitoyl‐2‐oleoyl‐sn‐glycero‐3‐phosphoglycerol (POPG) and conducted molecular dynamics (MD) simulations at a 1‐µs timescale within the GROMACS framework. Consistent with prior simulation results, HW and GW formed large and small clusters interacting with membrane components, respectively, while RW remained attached to the membrane surface as individual molecules. This indicates that HW and GW interact with microbial membranes in self‐assembled conformations (Figure 1E). Further molecular analyses revealed that exposure to BPs reduced the density of the phospholipid bilayer, thereby deteriorating membrane fluidity (Figure S12, Supporting Information). Collectively, these findings demonstrate that the BPs disrupt membrane integrity by binding to both phospholipid headgroups and acyl tails, subsequently penetrating bacterial interiors to perturb metabolic processes and induce cell death.

Moreover, the BPs maintained high stability under physiological ionic strength, 50% and 25% serum environments, and extreme pH/temperature conditions without significant loss of activity (Tables S5 and S6, Supporting Information), confirming their clinical applicability as broad‐spectrum antimicrobial agents. Cytotoxicity assessments showed that the peptides exhibited <5% hemolysis against hRBCs at the highest concentration, and cell viability remained >80% for RAW 264.7, IPEC‐J2, and PK‐15 cells after 4‐hour exposure, verifying their low cytotoxicity and cross‐species biocompatibility (Figure S13, Supporting Information).

Digestive enzyme resistance studies demonstrated that HW and GW maintained stable antimicrobial activity after 8 h of treatment with trypsin and gastric/intestinal fluids, while RW lost its activity within 1 h due to rapid degradation by trypsin (Tables S7 and S8). Tricine‐SDS‐PAGE and HPLC confirmed that RW was fragmented into small peptides after enzymatic digestion, accompanied by a decrease in [θ] signals and the formation of aggregates (Figure 1F; Figure S14, Supporting Information). Concurrently, zeta potential shifts indicated the exposure of charged residues (Figure S14H, Supporting Information). Molecular docking revealed that RW exhibited the strongest binding affinity with trypsin (ΔG = −62.43 kcal mol^−1^) but showed low mechanical stability (Figure S15 and Tables S9–S11, Supporting Information). Selectivity index (SI)‐based screening (**Table** **1**) identified HW as a promising candidate for targeting intestinal infections, owing to its highest SI value (93.97) and robust anti‐enzymatic activity (AI = 102.34). These findings validate the design strategy centered on proline hinge–Trp‐zipper–arginine synergistic interactions.

**Table 1:** The minimum hemolytic concentration (MHC), geometric mean of the minimum inhibitory concentration (GM), therapeutic index (TI), anti‐enzymolysis index (AI), and selectivity index (SI) of the engineered BPs were determined.

BPs		HW	RW	GW
MHC^a^ ^)^		>256.00	>256.00	>256.00
GM^b)^	Gram‐ bacteria	3.10	2.78	27.66
	Gram+ bacteria	5.88	7.80	42.44
	Fungi	11.46	6.68	43.55
	ALL	5.93	5.25	37.11
TI^c)^	Gram‐ bacteria	165.16	184.17	18.51
	Gram+ bacteria	87.07	65.64	12.06
	Fungi	44.68	76.65	11.76
	ALL	86.29	97.49	13.80
AI^d)^	Trypsin	89.09	9.92	66.74
	Chymotrypsin	100.00	59.46	63.00
	Pepsase	112.25	70.71	89.09
	Proteinase K	94.39	84.09	50.00
	Papain	118.92	84.09	79.37
	ALL	102.34	49.43	68.30
SI^e)^		93.97	69.42	30.70

^a)^ MHC is the minimum hemolytic concentration that causes 10% hemolysis of hRBCs. When no detectable hemolytic activity was observed at 128 µm, a value of 256 µm was used to calculate the TI.

^b)^ The GM value is the geometric mean of the MIC value.

^c)^ TI = MHC/GM.

^d)^ The AI value is the reciprocal of the geometric mean of the MIC value of engineered BPs under protease treatment.

^e)^ SI is the geometric mean of the TI value and AI value.

In vivo experiments demonstrated that HW pretreatment significantly alleviated hepatic and renal injury (Figure S16, Supporting Information), and reversed jejunal villi atrophy and inflammatory infiltration (Figure S17C, Supporting Information). Pathogenic mechanism analysis revealed that ETEC K88 preferentially colonized the jejunum (3.22 × 10^6^ CFU mL^−1^) and translocated to the liver and kidneys, while no colonization was observed in other organs (Figure S18, Supporting Information). HW reduced oxidative stress by upregulating jejunal antioxidant markers (e.g., SOD, GSH) and inhibiting lipid peroxidation (Figure S17D–F, Supporting Information), with its effects potentially linked to the activation of the GPX4‐mediated GSH‐GSSG cycle, suggesting that HW suppresses ferroptosis by modulating redox homeostasis. The cell survival rate following treatment with multi‐cell death agonists demonstrated this finding (Figure S19, Supporting Information). Translational validation in large mammals showed that HW markedly mitigated ETEC K88‐induced diarrhea in weaned piglets (progressing from soft stools on day 3 to watery diarrhea by day 4) and inhibited intestinal oxidative damage (Figure S22B,D–F, Supporting Information). Mechanistically, ETEC K88 disrupted the structural integrity of jejunal GPX4 protein, whereas HW pretreatment preserved its expression levels (Figure S22G, Supporting Information). Collectively, these findings substantiate HW as a target‐active molecule with robust antimicrobial efficacy both in vitro and in vivo.

### 2.2 The Antimicrobial Mechanism of BPs Disrupting Bacterial Membrane Components

**Figure 2:**
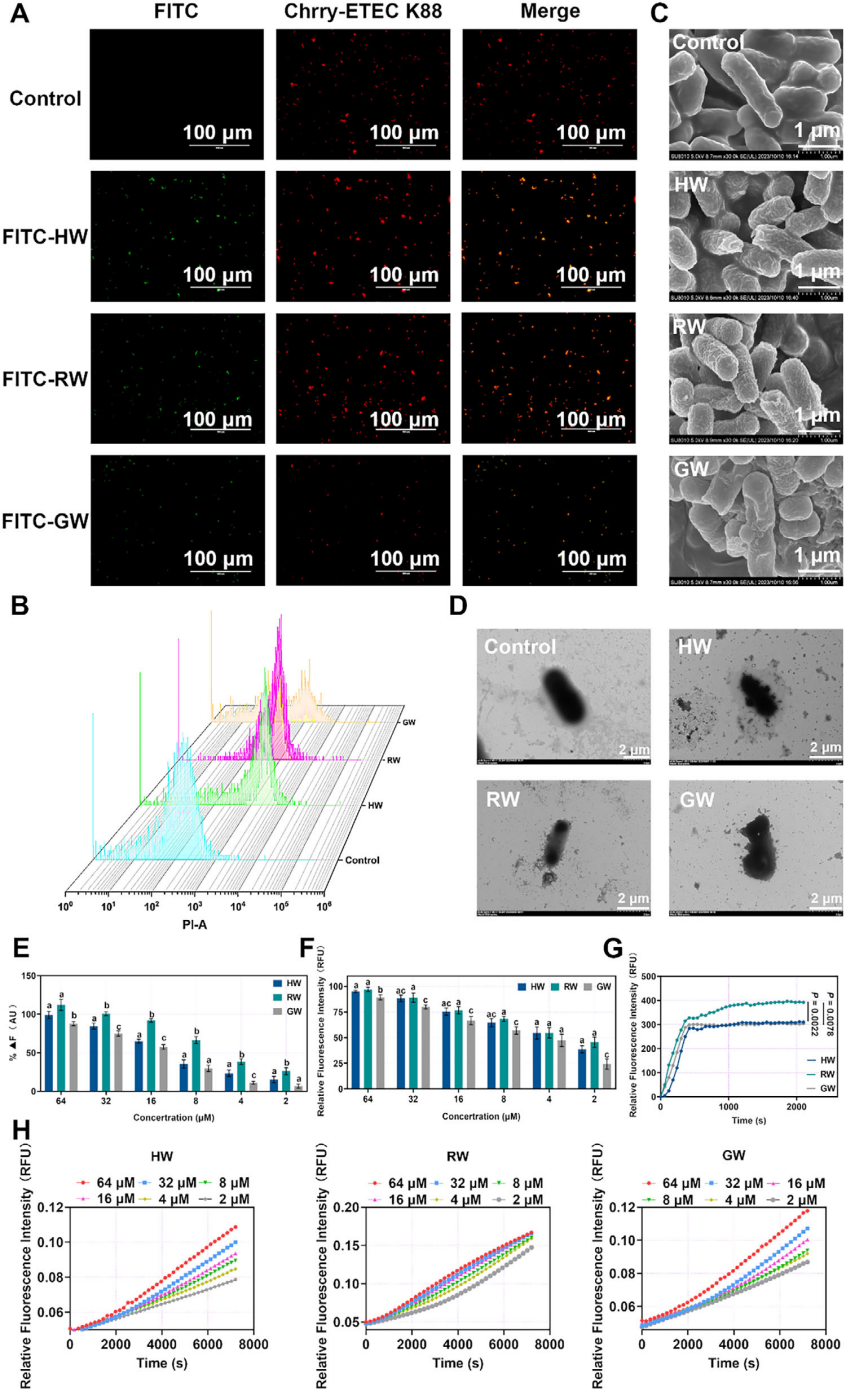
Engineered BPs self‐assemble in situ on bacterial membranes, leading to membrane disruption. A) Membrane damage in ETEC K88 after treatment with engineered BPs, observed under a fluorescence microscope. Green signal: FITC‐BPs; red signal: ETEC K88 expressing mCherry; scale bar: 100 µm. B) Flow cytometry of ETEC K88 treated with engineered BPs. C) SEM images of ETEC K88 after engineered BPs treatment; scale bar: 1 µm. D) TEM images of ETEC K88 in the control, HW, RW, and GW groups; scale bar: 2 µm. E) Binding interaction between LPS and engineered BPs. F) Dose‐dependent destruction of ETEC K88 outer membrane integrity by different concentrations of engineered BPs. G) Disruption of the plasma membrane potential in ETEC K88 caused by engineered BPs. H) The inner membrane permeability of the BPs. The hydrolysis of o‐nitrophenyl‐β‐D‐galactoside (ONPG) due to the release of cytoplasmic β‐galactosidase of ETEC K88 was measured spectroscopically at absorbance of 420 nm as a function of time. Statistical differences between groups exposed to the same concentration were analyzed by one‐way ANOVA followed by Tukey's post hoc test (*n *= 3, different letters indicate significant differences *p* < 0.05) E, F, and H).

To investigate whether the cytotoxic effect of BPs against ETEC K88 is attributable to their disruption of the bacterial membrane system, we performed comprehensive morphological analyses. ETEC K88 cells transfected with mCherry, a red fluorescent protein, were co‐incubated with FITC‐labeled BPs for 1 h. Fluorescence imaging using an inverted microscope demonstrated that the red fluorescence emitted by mCherry was uniformly distributed throughout the ETEC K88 cells and largely co‐localized with the green fluorescence from FITC‐BPs (**Figure** **2**A). To quantitatively evaluate the degree of membrane damage induced by BPs, the uptake of propidium iodide (PI), which intercalates with nucleic acids, was assessed via flow cytometry. The results indicated that PI effectively bound to the nucleic acids of ETEC K88 (Figure 2B), with the percentages of PI‐positive cells following treatment with HW, RW, and GW reaching 67.20%, 84.80%, and 31.50%, respectively. Furthermore, scanning electron microscopy (SEM) and transmission electron microscopy (TEM), combined with counterstaining techniques, were employed to directly visualize structural alterations in the bacterial membrane (Figure 2C,D). Morphological changes such as membrane contraction, budding, and deformation were clearly observed after BP treatment. Collectively, these results indicate that BPs exert their antibacterial activity by compromising the integrity of the ETEC K88 membrane system.

The complex, multi‐layered membrane system of Gram‐negative bacteria necessitates a sequential penetration of its layers by bacteriocins to thoroughly investigate their interactions with individual components. ETEC K88 was chosen as a representative model for mechanistic studies. Upon contact with ETEC K88, the outermost antigenic layer—specifically, the lipopolysaccharide (LPS) layer—is the first barrier encountered.^[^ ^26^ ^]^ This interaction is primarily influenced by the negatively charged phosphate groups (PO^4−^) present in LPS, which interact electrostatically with the arginine residues located in the bacteriocin's pocket region. Preliminary experimental results demonstrated that the binding of BPs to LPS follows a concentration‐dependent mechanism (Figure 2E). Once the initial defensive barrier is compromised, the underlying outer membrane serves as the subsequent line of defense for BPs. The interaction of BPs with the outer membrane follows a concentration‐dependent trend, with permeability exceeding 50% at twice the MIC. However, at this concentration, the permeation effect of RW does not show a statistically significant difference compared to HW and GW (Figure 2F). These results suggest that RW is capable of effectively penetrating the defense barriers of ETEC K88, potentially due to the high arginine content in its hydrophobic tail. Nevertheless, its ability to disrupt the outer membrane is comparable to that of HW and GW. The plasma membrane serves as a critical component of the microbial membrane system, facilitating electron transport and influencing the respiratory chain in Gram‐negative bacteria. Disruption of this membrane may lead to metabolic dysfunction in ETEC K88, prompting us to assess the impact of BPs on plasma membrane potential (Figure 2G). Notably, RW again demonstrated a pronounced effect on membrane integrity, which may be attributed to the influence of the positively charged arginine residues on the transmembrane potential. Finally, when BPs targeted the inner membrane—the last line of defense of ETEC K88—their effects remained concentration‐dependent (Figure 2H). Collectively, these findings indicate that BPs exert antibacterial activity by disrupting the LPS layer and increasing the permeability of both the outer and inner membranes, while also impairing membrane potential.

### 2.3 Nanomotor with both Antibacterial and Peroxide Elimination Capabilities

**Figure 3:**
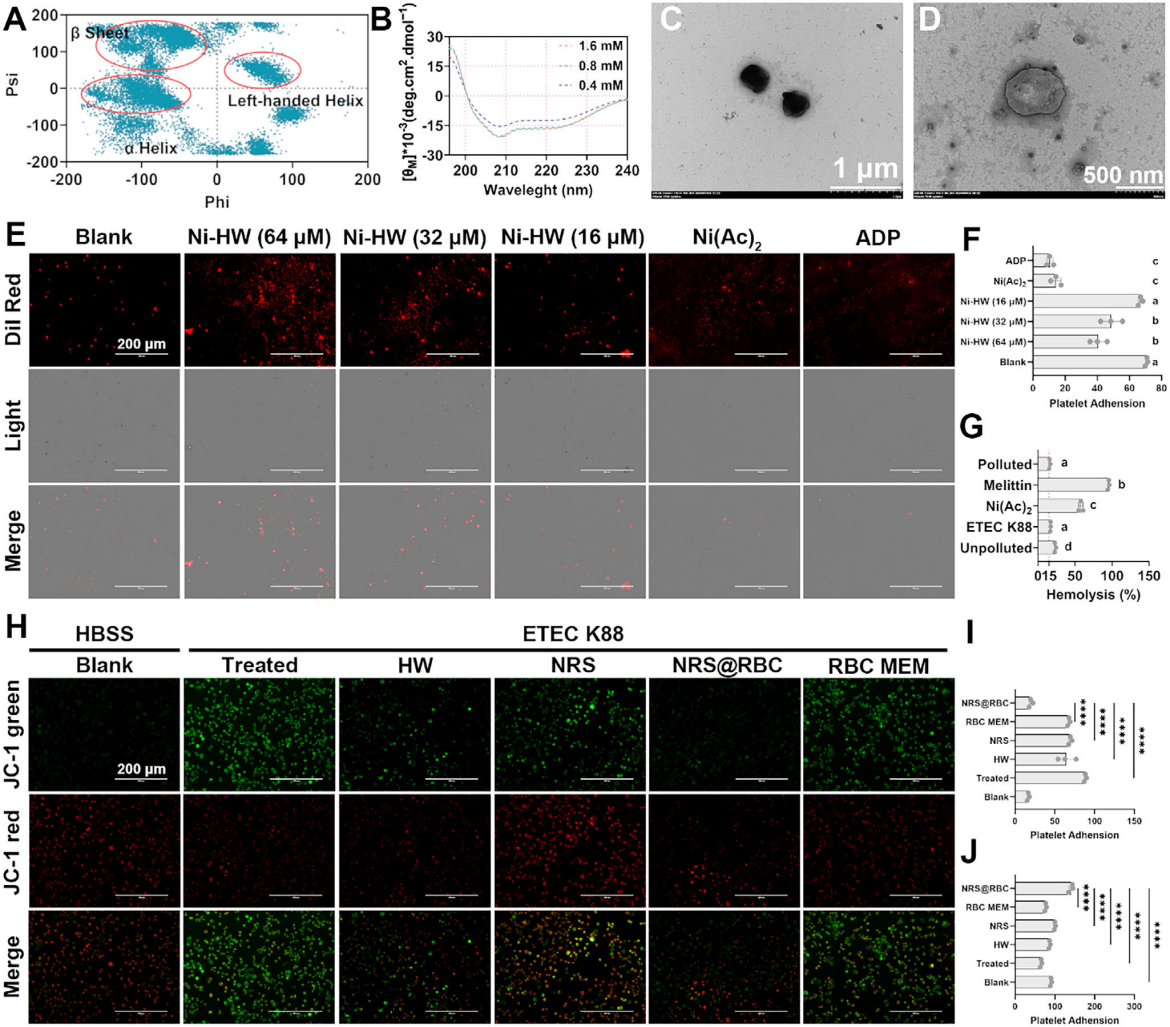
Structural Characterization and Biological Activity of Nanomotor Storage. A) The secondary structure abundance of HW was determined through Raman spectroscopy analysis following a GROMACS‐based molecular dynamics simulation. B) CD spectra of Ni‐HW in different concentrations of Ni(Ac)_2_ solutions. TEM observation of the morphological and structural features of C) Ni‐HW and D) NRS@RBC; scale bar: 1 µm and 500 nm. E) Representative fluorescence images depicting platelet adhesion and F) corresponding quantitative analysis following treatment with various compounds. Platelets were stained with Dil, showing an orange‐red fluorescence; scale bar: 200 µm. G) Hemolytic activity of Ni‐HW in the presence and absence of contamination. H–J) Immunofluorescence staining and semi‐quantitative evaluation of JC‐1 in IPEC‐J2 cells across experimental groups. Statistical differences between groups exposed to the same concentration were analyzed by one‐way ANOVA followed by Tukey's posthoc test (*n *= 3, different letters indicate significant differences *p* < 0.05) F, G, I, and J).

To enhance the formation of well‐defined structures, metal salts capable of coordinating with the imidazole ring of histidine residues were introduced. These salts facilitate the formation of a structure analogous to the active site of natural enzymes, thereby reinforcing the ordered nanostructure of HW and mimicking the catalytic activity characteristic of nanozymes.^[^ ^27^ ^]^ According to GROMACS simulations, the β‐conformation may represent the dominant structural feature of BPs assembly (**Figure** **3**A). Subsequently, CD spectroscopy was employed to analyze the secondary structure of HW in the presence of varying concentrations of nickel salts. Overall, the β‐sheet conformation of HW transitioned into an α‐helix conformation across all tested nickel acetate concentrations (Figure 3B). Through systematic screening of various nickel acetate concentrations, it was determined that 0.4 mm Ni(Ac)_2_ optimally promoted the formation of a distinct α‐helix secondary structure and fully developed nanomicelles (designated as Ni‐HW) (Figure 3C). Furthermore, UV–vis spectroscopic analysis revealed that the absorbance of the nickel acetate ion‐coordinated polypeptide in the 200–250 nm range was markedly higher than that of either nickel acetate ions or the polypeptide alone (Figure S23, Supporting Information). This spectral region is typically associated with π→π transitions of the peptide backbone, indicating that Ni^2+^ influences the electronic distribution within the backbone and suggesting potential coordination involving carbonyl oxygen or nitrogen atoms. Moreover, in the 350–450 nm range, the nickel acetate ion‐coordinated polypeptide displayed a distinct shoulder absorption peak, accompanied by a slight redshift relative to the spectrum of free Ni^2+^. This redshift implies an enhanced coordination field strength around the nickel center, which can be attributed to the interaction between Ni^2+^ and donor atoms—specifically oxygen and nitrogen—present in the peptide matrix. These findings demonstrate that HW self‐assembles into α‐helix‐based nanostructures and that this organization is effectively driven by Ni(Ac)_2_‐mediated metal coordination.

During a cytotoxicity assessment of Ni‐HW on human RBCs, we inadvertently used bacteria‐contaminated plasma to isolate purified RBCs. Although the initial results indicated low cytotoxicity of Ni‐HW toward RBCs, this issue was identified promptly after the experiment, prompting us to repeat the cytotoxicity test. Surprisingly, the follow‐up results revealed that Ni‐HW induced partial hemolysis of red blood cells and platelet aggregation, despite the hemolysis rate remaining below 15% (Figure 2E–G). We hypothesized that Ni‐HW might preferentially interact with bacteria, thereby reducing its impact on platelet function. To evaluate this hypothesis, we pre‐incubated Ni‐HW with ETEC K88 prior to exposure. The findings demonstrated that Ni‐HW retained cytotoxic effects on RBCs even after binding to the bacterial membrane, contradicting our initial assumption. It is plausible that Ni‐HW exceeded the threshold concentration required for membrane interaction, and the sub‐MIC concentration alone may not induce significant hemolysis. Regardless, these observations prompted us to explore the potential of coating Ni‐HW with RBC membranes to mitigate its hemolytic toxicity.

To prepare Ni‐HW@RBC (NRS@RBC), RBC membranes obtained via the swelling method were coated onto the surface of Ni‐HW using the extrusion technique. Following membrane coating, the hydrodynamic size of NRS@RBC increased from ≈531.2 nm to ≈1484.0 nm. Furthermore, the ζ‐potential of NRS@RBC was higher than that of Ni‐HW (Figure S25, Supporting Information), which can be attributed to the enhanced surface charge density resulting from Ni^2+^ coordination. TEM analysis also revealed a visible coating layer of RBC on Ni‐HW, further confirming the successful fabrication of NRS@RBC (Figure 3D). Subsequently, the biological activity and catalytic performance of NRS@RBC were evaluated in comparison with those of Ni‐HW and Ni^2+^ to assess their synergistic effects on H_2_O_2_ consumption and O_2_ generation. As shown in the time‐dependent H_2_O_2_ assay (Figure S26, Supporting Information), NRS and NRS@RBC decomposed 40.91% and 42.31% of H_2_O_2_, respectively, within 30 min. Additionally, NRS produced 18.81 mg L^−1^ of O_2_ within 10 min, while NRS@RBC generated 17.70 mg L^−1^ under identical conditions, indicating that the presence of RBC did not significantly affect oxygen production.

Furthermore, the inflammatory chemotactic properties of NRS@RBC enable it to effectively penetrate throughout inflamed tissues. Building on this observation, the permeability of NRS@RBC across an in vitro mucus intestinal barrier was further evaluated. Fluorescently labeled formulations were introduced into the apical chamber, and after a 1 h incubation, fluorescence in the basolateral compartment of IPEC‐J2 cells or mouse intestinal crypt organoids was assessed using fluorescence microscopy. As shown in the fluorescence analysis (Figure S27, Supporting Information), the fluorescence intensity in the NRS@RBC + H_2_O_2_ group was significantly higher than that in the NRS@RBC group, indicating that H_2_O_2_ enhances the nanomotor's permeability through the mucus layer, thereby improving the delivery efficiency of the oral drug delivery system. Given these favorable in vitro permeability characteristics, we proceeded to investigate the in vivo intestinal barrier penetration of NRS@RBC. Consistent with the in vitro findings, a substantial proportion of NRS@RBC traversed the mucus layer in colitis‐induced mice, whereas minimal penetration was observed in healthy control mice. Collectively, these results demonstrate that the Janus‐structured NRS@RBC can efficiently penetrate the mucus barrier via self‐propulsion, highlighting its potential for targeted therapeutic applications in inflammatory bowel disease.

The inflammatory response associated with pathogenic colitis is typically accompanied by the accumulation of substantial amounts of lipid peroxides.^[^ ^28^, ^29^ ^]^ Excessive lipid peroxide levels can trigger programmed cell death, such as ferroptosis, and lead to the release of numerous inflammatory cytokines.^[^ ^30^, ^31^ ^]^ To further confirm that NRS@RBC helps maintain mitochondrial membrane potential, JC‐1 staining was employed to label mitochondria and assess potential alterations. IPEC‐J2 cells were pre‐treated with NRS, NRS@RBC, or RBC@MEM for 2 h before being exposed to continuous bacterial stimulation for an additional 4 h. As illustrated in Figure 3H–J, bacterial infection significantly decreased mitochondrial membrane potential; however, treatment with NRS and NRS@RBC effectively preserved mitochondrial membrane integrity. These findings indicate that both NRS and NRS@RBC possess antioxidant properties, preventing lipid peroxide formation through enzyme‐like catalytic activity.

### 2.4 Nanomotor Storage with Endotoxin Storage Function

**Figure 4:**
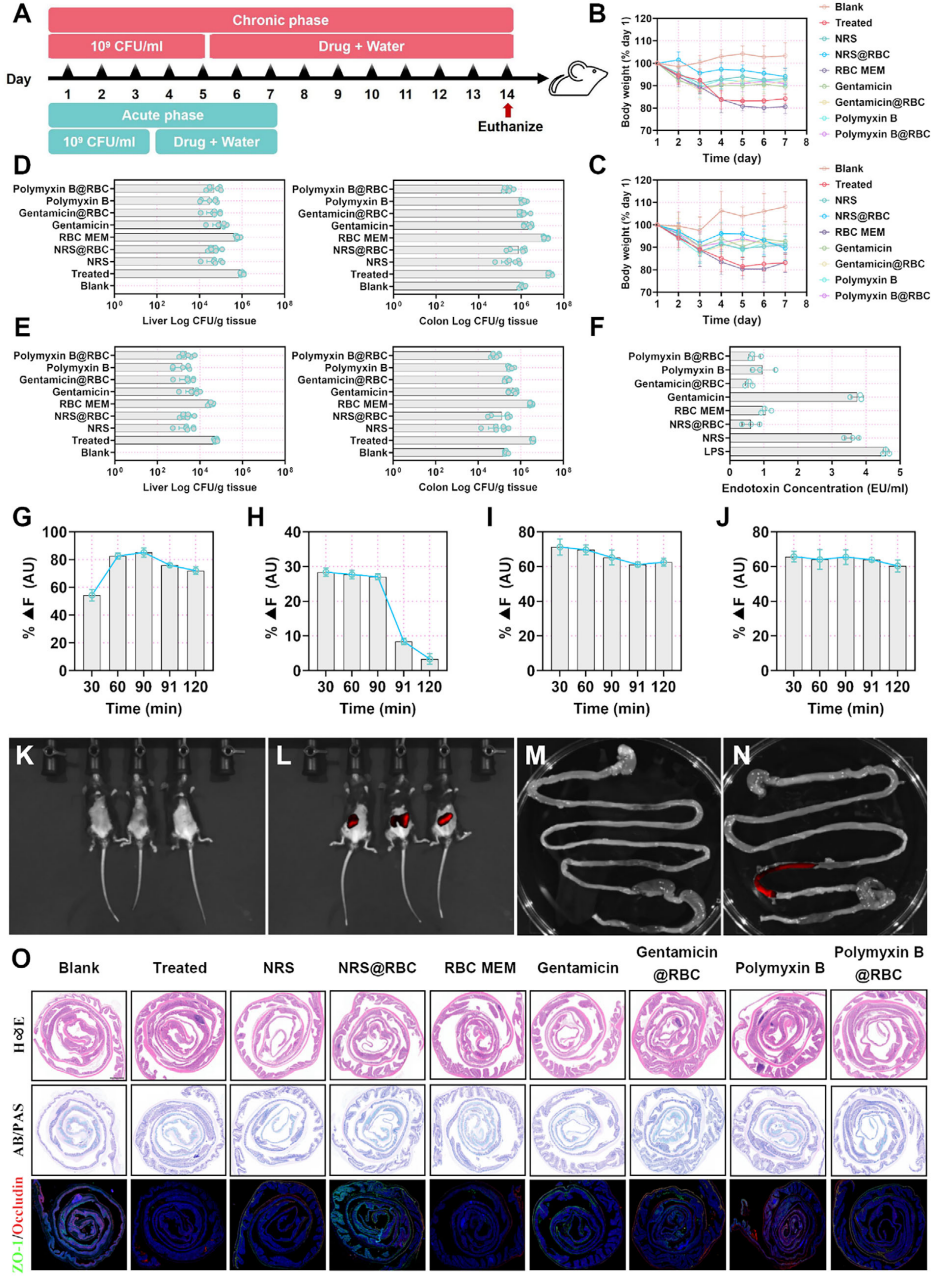
Various compounds and their membrane coating materials notably alleviated the pathogenic colitis. A) Schematic diagram of drug intervention plan for pathogenic colitis. Body weight changes over time in nine groups of mice during B) the acute phase and C) the chronic phase, expressed as a percentage of body weight on day 0 (*n* = 6). Bacterial loads in liver and colon tissues across nine groups of mice during D) the acute phase and E) the chronic phase (*n* = 6). F) The effect of membrane‐coated materials on endotoxin reduction compared to non‐membrane‐coated materials (*n* = 3). The capacity of G) NRS@RBC, H) RBC MEM, I) gentamicin@RBC, and J) polymyxin B@RBC to bind LPS was evaluated (*n* = 3). In vivo fluorescence imaging of mice following oral administration of K and M) physiological saline or (groups L and N) Cy5‐labeled NRS@RBC. O) Colon Swiss rolls images stained with H&E and AB/PAS, along with immunofluorescence staining for ZO‐1 and occludin (scale bar: 1000 µm). Statistical differences between groups exposed to the same concentration were analyzed by one‐way ANOVA followed by Tukey's post hoc test (*n* = 6 for B‐E, *n* = 3 for F–J, different letters indicate significant differences *p* < 0.05).

To further evaluate the in vivo bacterial neutralization efficacy of NRS and NRS@RBC, a pathogenic colitis mouse model was established through oral gavage of a high‐concentration ETEC K88 suspension (2 × 10^9^ CFU per mouse) (**Figure** **4**A). Three days postinfection, mice were orally administered with NRS, NRS@RBC, or RBC MEM, respectively, while gentamicin and polymyxin B were used as antibiotic control groups. The bacterial distribution across various organs and tissues was subsequently monitored. On day 7, bacterial counts in the colon and liver of mice treated with NRS, NRS@RBC, and antibiotics were significantly reduced compared to those in the control group (Figure 4D). This reduction in bacterial burden correlated with a decrease in pro‐inflammatory cytokine levels (Figure S26, Supporting Information). However, an unexpected phenomenon was observed in mice subjected to long‐term treatment. Although bacterial loads in the intestine and liver of mice euthanized on day 14 were markedly decreased (Figure 4E), a more pronounced inflammatory cytokine response was detected in all groups except for the partially red blood cell membrane‐coated treatment group, compared to short‐term treatment (7 days) (Figure S28, Supporting Information). Based on the cytokine data from mice treated with NRS alone, we ruled out the possibility of immunogenicity associated with NRS. *E. coli*, a Gram‐negative bacterium, releases endotoxins (primarily LPS) during infection or membrane lysis,^[^ ^32^ ^]^ which can trigger excessive secretion of pro‐inflammatory cytokines in infected tissues. Notably, long‐term treatment resulted in nearly complete clearance of ETEC K88 adhered to the intestinal mucosa. Therefore, we hypothesize that a substantial amount of endotoxin was released during the bacterial lysis phase in the long‐term treatment period. Although these endotoxins did not induce mortality or diarrhea, they continuously stimulated inflammatory responses in the mucosal tissues. Furthermore, the red blood cell membrane, being an amphiphilic phospholipid bilayer, can act as a molecular decoy to attract lipid peroxides or LPS.^[^ ^33^ ^]^ Thus, the membrane coating may function as a reservoir for concentrating LPS, preventing its direct interaction with intestinal epithelial cells. Another supporting rationale for this hypothesis is that polymyxin B treatment, which lacks membrane coating, did not induce a significant increase in inflammatory cytokines, likely due to its strong binding affinity for LPS. In contrast, gentamicin, which exhibits potent bactericidal activity, was associated with elevated TNF‐α levels.

To validate this hypothesis, we employed the limulus amebocyte lysate (LAL) assay to assess residual endotoxin levels in the presence of red blood cell membrane coatings.^[^ ^34^ ^]^ Following co‐incubation of 10 ng of LPS with either membrane‐coated or nonmembrane‐coated materials for 4 h, the results are presented in Figure 4F. Compared to nonmembrane‐coated materials, the membrane‐coated formulations substantially attenuated the chromogenic response indicative of endotoxin activity, whereas gentamicin failed to reduce endotoxin levels under any experimental conditions. To evaluate the capacity of membrane‐coated materials to bind and sequester LPS, we utilized the BODIPY TR Cadaverine fluorescent probe as a reporter of LPS interaction. As the incubation time between membrane‐coated materials and LPS increased, a marked enhancement in fluorescence intensity was observed. Once the fluorescence signal reached a plateau, Triton X‐100 was introduced to disrupt the red blood cell membrane vesicles. A rapid decrease in fluorescence intensity was observed for RBC@MEM (Figure 4G–J), indicating the effective binding and subsequent release of LPS. These findings collectively confirm that the membrane‐coated, LPS‐binding formulation exhibits a robust capacity for endotoxin neutralization.

Another challenge in validating the endotoxin‐neutralizing capability of the membrane‐coated material is determining whether LPS constitutes the primary source of endotoxins. To address this, we removed the LPS layer from the surface of ETEC K88 using EDTA chelation, and the formation of protoplasts confirmed the successful removal of the LPS layer.^[^ ^35^ ^]^ Endotoxin levels were then measured, and no significant difference was observed in the endotoxin content in the supernatant of protoplasts treated with NRS, NRS@RBC, polymyxin B, or polymyxin B@RBC compared to that of untreated ETEC K88 with an intact LPS layer (Figure S28, Supporting Information). These results indicate that the neutralization of LPS is essential for the reduction of pro‐inflammatory cytokine production.

An essential characteristic of targeted oral drug delivery systems is the capability to efficiently and stably reach the target lesion site.^[^ ^36^ ^]^ NRS@RBC‐Cy5 was orally administered to mice, and fluorescence images were acquired 2 h postadministration using an in vivo imaging system. As shown in Figure 4K–N, NRS@RBC reached the colonic region within 2 h after oral administration of NRS@RBC‐Cy5. To further demonstrate the effectiveness of our approach, the therapeutic efficacy of NRS@RBC was evaluated in both acute and chronic bacterial colitis models (Figure 4O). Histopathological changes were assessed using H&E staining. Treatment with NRS@RBC preserved the integrity of the colonic epithelium, reduced infiltration of inflammatory cells, and alleviated inflammatory manifestations in the colon. Immunofluorescence analysis revealed that, compared to the NRS group, NRS@RBC significantly enhanced the expression levels of ZO‐1 and occludin in colonic tissues. To assess the in vivo safety profile of NRS@RBC following oral administration, serum biochemical parameters were analyzed. No significant differences were observed in the levels of lactate dehydrogenase, creatine kinase, blood urea nitrogen, aspartate aminotransferase, or alanine aminotransferase across treatment groups (Figure S29A,B, Supporting Information). Histological examination of major organs (liver, kidney, lung, and spleen) further confirmed the absence of notable cellular damage or pathological alterations following various treatments (Figure S29C, Supporting Information).

### 2.5 Regulation of Intestinal Microbiota

**Figure 5:**
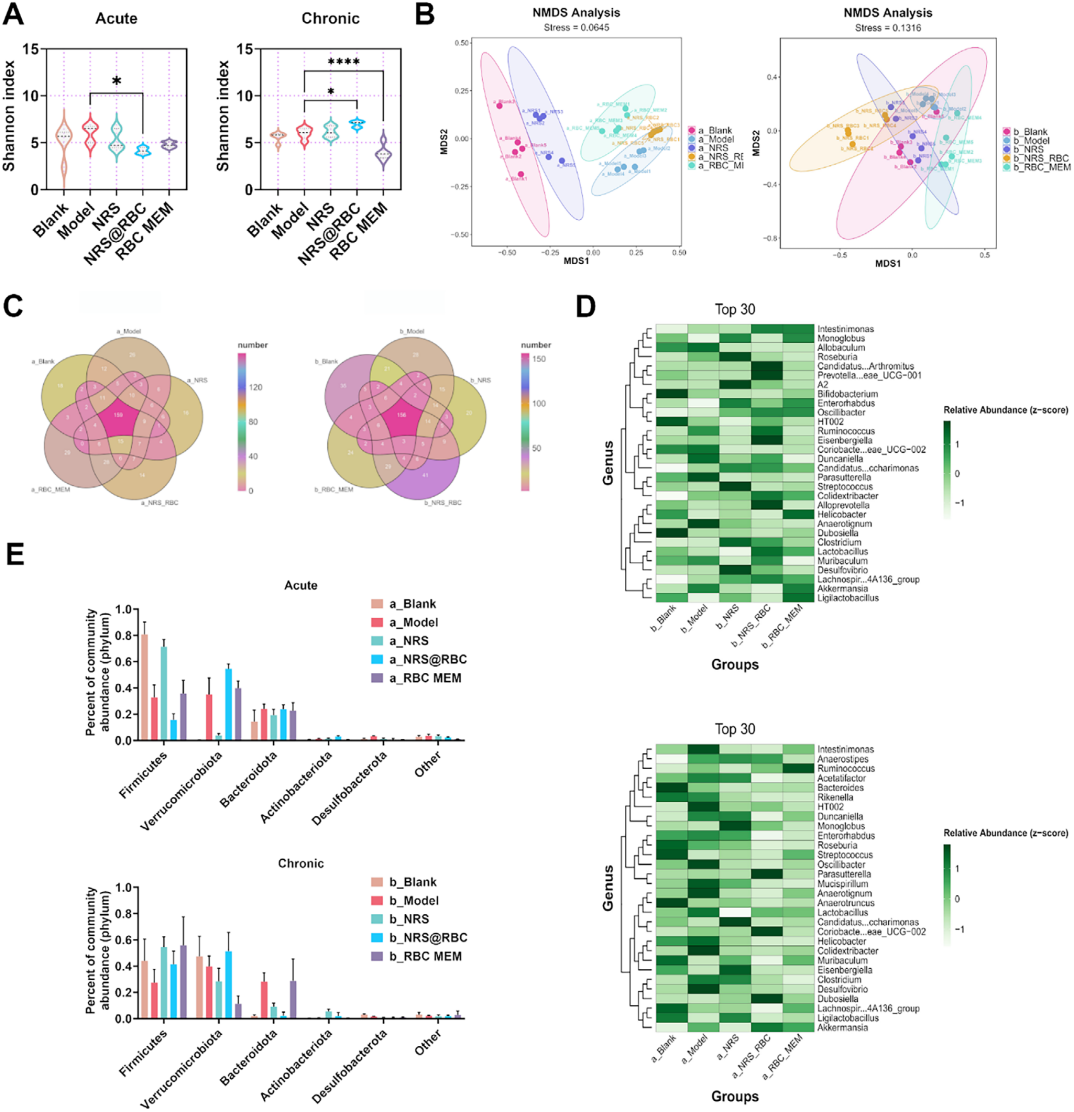
NRS@RBC treatment effectively restored intestinal microbiota homeostasis in mice following ETEC K88‐induced disruption. A) The Shannon index of operational taxonomic units observed during both the acute and chronic phases in mice treated with ETEC K88 reflects the α‐diversity of the intestinal microbial community. B) The NMDS score plot illustrates the β‐diversity of the gut microbiota. C) Venn diagrams depicting different sets of species groups. D) Heatmap illustrates the relative abundance of each taxonomic group at the genus level across individual mouse samples. E) Histograms depicting the horizontal distribution of the door across each group. Statistical differences between groups exposed to the same concentration were analyzed by one‐way ANOVA followed by Tukey's post hoc test (*n* = 5, ^*^ indicates *p* < 0.05, ^****^ indicates *p* < 0.001).

The pathogenesis of infectious colitis is closely associated with the dysbiosis of the intestinal symbiotic microbiota.^[^ ^37^, ^38^ ^]^ To investigate the effects and distinctions of NRS@RBC on the composition and abundance of the gut microbiota, 16S ribosomal RNA (rRNA) sequencing was employed to analyze the microbial profiles in the colonic contents of both acute and chronic disease phases. Alpha diversity analysis, based on the Shannon index of observed species, demonstrated that NRS@RBC administration during the acute phase significantly enhanced microbial richness, indicating its potential to restore the disrupted microbiota (**Figure** **5**A). Linear discriminant analysis (LDA) effect size (LEfSe) was applied to identify taxonomic features that were differentially abundant across groups. The LDA scores highlighted dominant taxa and their relative influence across taxonomic levels from phylum to genus. Compared with the healthy control group and drug‐treated groups, pathogen‐induced colitis altered the overall composition of the intestinal microbiota (Figure S30, Supporting Information). Furthermore, the non‐metric multidimensional scaling (NMDS) plot revealed a clear separation between pathogen‐infected colitis mice and healthy controls (Figure 5B). At the phylum and family levels, the observed differences in community structure and species distribution indicated that NRS@RBC increased the abundance of mucin‐degrading and sugar‐utilizing taxa such as *Akkermansia* and *Muribaculaceae* during the acute phase, while decreasing the abundance of pro‐inflammatory taxa such as *Fusobacteriaceae*. Additionally, NRS enhanced the population of beneficial probiotic taxa such as *Lactobacillaceae* (Figure 5E; Figure S29, Supporting Information). Under long‐term treatment, both NRS and NRS@RBC promoted the proliferation of *Lactobacillaceae*. Collectively, NRS and NRS@RBC suppressed the colonization and overgrowth of ETEC K88 through two primary mechanisms: 1) direct antimicrobial activity; and 2) enhancing probiotic populations to competitively inhibit pathogenic bacteria.

### 2.6 Therapeutic Efficacy Against ETEC K88‐Induced Colitis

**Figure 6:**
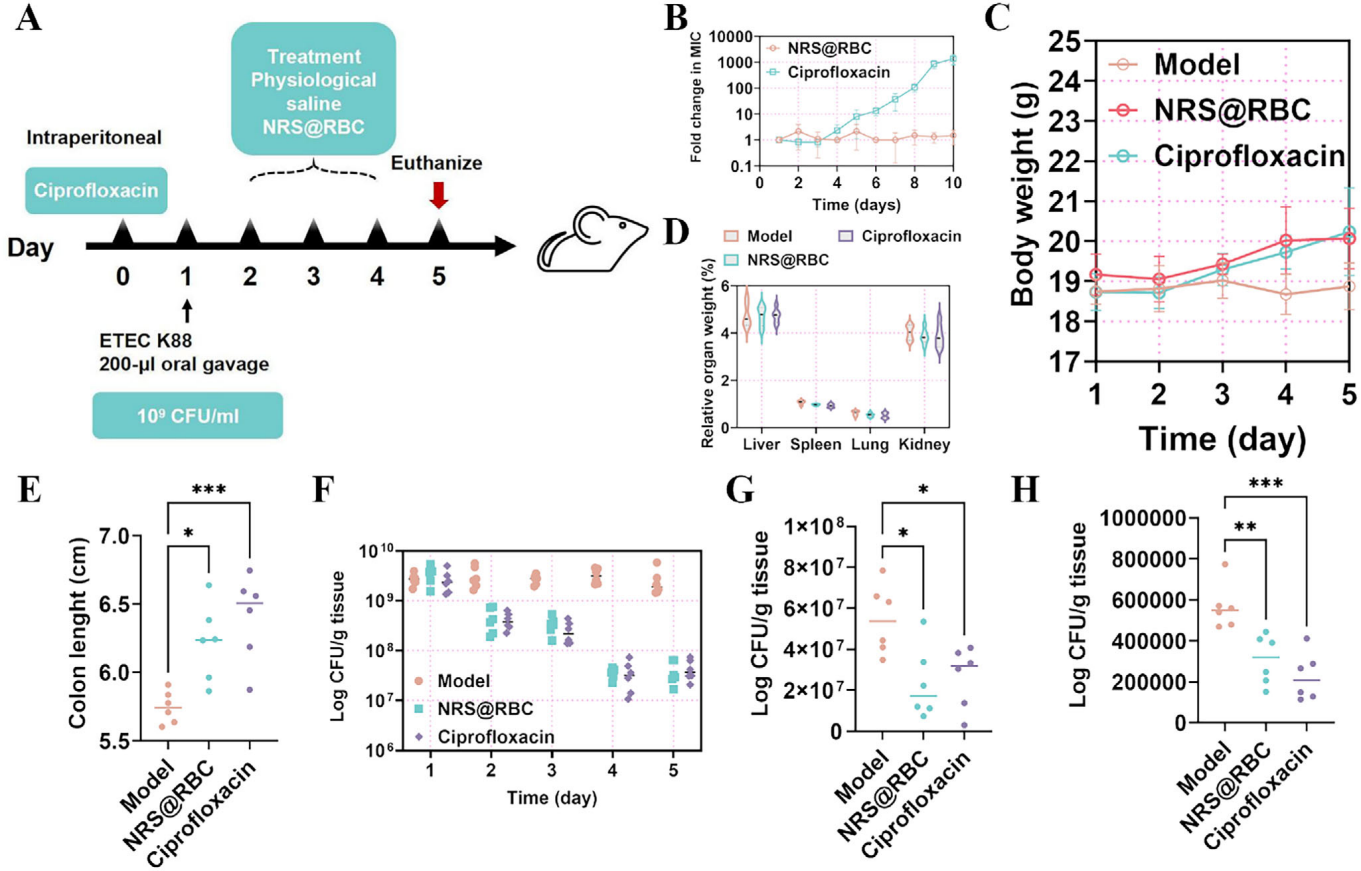
NRS@RBC and ciprofloxacin treat ETEC K88 infections in mice. A) Schematic diagram of drug intervention plan for ETEC K88 infections. B) The emergence of resistance in ETEC K88 when exposed to antibiotics at sub‐MIC levels. C–E) Mice body weight, relative organ weight, and colon length. *n *= 6 mice per group. F–H) Bacterial burden of ETEC K88 in the F) feces, contents of the G) colon, and H) colon tissues from infected mice. Each point represents a mouse. *n *= 6 for each group. Statistical differences between groups exposed to the same concentration were analyzed by one‐way ANOVA followed by Tukey's post hoc test (*n* = 6, ^*^ indicates *p* < 0.05, ^**^ indicates *p* < 0.01 and ^***^ indicates *p* < 0.01).

To evaluate the therapeutic efficacy of NRS@RBC against clinical opportunistic pathogens, we employed an ETEC K88 infection model to compare the protective effects of NRS@RBC and the commercially available antibiotic ciprofloxacin in the context of intestinal infection. Ciprofloxacin, a fluoroquinolone antimicrobial agent, exhibits potent bactericidal activity against common clinical opportunistic pathogens;^[^ ^39^, ^40^ ^]^ however, its administration may promote the overgrowth of ETEC K88. In our previous studies, no spontaneous resistance of ETEC K88 to NRS@RBC nanoparticles was observed, which may be attributed to its bactericidal mechanism involving the disruption of bacterial cell walls and membranes (**Figure** **6**A). In contrast, the minimum inhibitory concentration (MIC) of ciprofloxacin increased by 1024‐fold after only 10 generations of bacterial passage under sub‐MIC exposure (Figure 6B).

A sustained ETEC K88 intestinal infection model was established, and ciprofloxacin was administered one day prior to model induction. No significant differences were observed in body weight or relative organ weight among the experimental groups (Figure 6C,D). Colon shortening is a characteristic outcome of colonic inflammation, which results from tissue edema and structural damage affecting colon length. Compared with the control group, treatment with NRS@RBC significantly restored colon length (Figure 6E). The fecal ETEC K88 load reached ≈10^9^ CFU g^−1^ within 24 h postinfection. While the control group maintained a high bacterial load (≈10^9^ CFU g^−1^) on day 5 post‐infection, both treatment groups exhibited notable bactericidal effects as early as 24 h after initiation of therapy. Following five days of treatment, the bacterial burden in colon tissues was markedly reduced in both the NRS@RBC and ciprofloxacin groups (Figure 6F). Both NRS@RBC and ciprofloxacin significantly decreased the bacterial load in both colon contents and tissues (Figure 6E,F). In summary, these results demonstrate that NRS@RBC exerts potent therapeutic effects in the treatment of antibiotic‐associated pathogenic colitis, with colon‐protective activity comparable to that of ciprofloxacin.

## 3 Conclusion

In summary, we designed and synthesized NRS@RBC nanomaterials, which possess the dual capabilities of stable intestinal delivery and efficient endotoxin sequestration. Building upon conventional antimicrobial peptides, Ni^2+^ was incorporated to stabilize the structural framework of NRS, thereby enhancing its antioxidant capacity in vitro. Upon coating with red blood cell membranes, NRS forms a bilayer vesicular structure that exhibits antibacterial, anti‐inflammatory, and epithelial barrier‐repairing properties in both acute and chronic colonic injuries induced by ETEC K88 in mice. Notably, NRS@RBC captures LPS via the red blood cell membrane and centrally stores them, enabling subsequent electrostatic binding with NRS in the core. This mechanism effectively prevents the excessive release of pro‐inflammatory cytokines caused by prolonged endotoxin stimulation during chronic treatment. Furthermore, NRS@RBC contributes to intestinal health by modulating the gut microbiota, demonstrating therapeutic efficacy comparable to that of the commercial antibiotic ciprofloxacin. By stabilizing antimicrobial peptides and neutralizing endotoxins, our nanomaterials offer a novel strategy for the precise treatment of intestinal pathogenic infections.

## 4 Experimental Section

### Animal Studies

For the in vivo studies, female C57BL/6 mice aged 6–8 weeks and weighing between 20 and 22 g were obtained from Liaoning Changsheng Biotechnology Co., Ltd. These animals were kept in an environmentally controlled facility set at a temperature of 23 ± 1 °C and humidity ranging from 40% to 60%, under a 12 h light‐dark cycle (from 08:00 to 20:00). Prior to the start of the experiments, the mice were allowed a week to adjust to the laboratory environment, during which they had free access to food and water. All procedures adhered to the guidelines endorsed by the Institutional Animal Care and Use Committee of Northeast Agricultural University, ensuring compliance with ethical standards for animal welfare (Approval Number: NEAUEC20240221).

### Statistical Analysis

Statistical analysis was conducted using GraphPad Prism 9 software. Statistical methods for each experiment are described in the corresponding Figure legends. Results are expressed as mean ± SD. For comparisons between two groups, a two‐tailed Student's *t*‐test was utilized, whereas for multiple group comparisons, one‐way ANOVA followed by Tukey's post hoc test was applied (using SPSS v22.0 for Windows). Statistical significance was set at a *p*‐value of less than 0.05. At least three independent in vitro antibacterial or cell experiments and at least six independent in vivo animal experiments were conducted.

## Conflict of Interest

The authors declare no conflict of interest.

## Supporting information



Supporting Information

## Data Availability

Data sharing is not applicable to this article as no new data were created or analyzed in this study.
