# Supplementary material for: An Engineered Solidified Peptide Hemocyte Sponge as Nanomotor Storage to Combat Bacterial Colitis
Source: Adv Sci (Weinh). 2025 Nov 29;13(8):e15620. doi: 10.1002/advs.202515620 (PMC12884796; doi:10.1002/advs.202515620)
Supplement: Supplementary file 1 — Supporting Information [file ADVS-13-e15620-s001.docx]

**Supporting Information**

**An Engineered Solidified Peptide Hemocyte Sponge as Nanomotor Storage to Combat Bacterial Colitis**

Yuxin Fang^1^, Jianming Yan^1^, Tongxin Yu^1^, Zichuan An^1^, Kaikai Lv, Chenyu Xue^1^, Hongyang Yu^2^, Na Dong^1^*, Anshan Shan^1^

^1^Laboratory of Molecular Nutrition and Immunity, College of Animal Science and Technology, Northeast Agricultural University, Harbin, 150030, PR China

^2^Harbin Med Univ, Affiliated Hosp 2, Dept Radiat Oncol, Harbin, Peoples R China

*Corresponding author. Email: ndong@neau.edu.cn (N.D.)

**Materials and Methods**

8-Anilino-1-naphthalenesulfonic acid (1, 8-ANS), Nile red, Triton X-100, Methylthiazolyltetrazolium (MTT), DMSO, trypsin, protease, pepsin, papain, proteinase K, ethanol, phosphate buffer saline, hyaluronic acid, sodium dodecyl sulfate (SDS), trifluoroacetic acid (TFE), bovine serum albumin (BSA), propidium iodide (PI), *E. coli* O111 LPS, N-phenyl-1-naphthylamine (NPN), and Disc3-5 were purchased from Sigma-Aldrich (China). BODIPY-TR-cadaverine (BC) and BODIPY 581/591 C11 were purchased from Thermo Fisher (China). Mueller–Hilton broth (MHB) Mueller-Hinton Agar (MHA) were purchased from AoboX (China). NaCl, KCl, CaCl₂, MgCl₂, ZnCl₂, FeCl₃, NH₄Cl, EDTA, ortho-nitrophenyl-β-galactoside (ONPG), gentamicin, streptomycin, ampicillin, vancomycin, metronidazole, erastin, RSL3, TNF-α + SM-164 and TSZ were purchased from Macklin (China). RPMI-1640 and fetal bovine serum were purchased from Hyclone. GPX4 rabbit polyclonal antibody and Cy3-labeled Goat anti-Rabbit IgG(H+L) were purchased from Beyotime (China).

1. *Machine Learning*

Data acquisition and processing: Peptide sequence data and corresponding bioactive tags were sourced from a self-developed peptide database. The data underwent preprocessing for subsequent modeling analysis. Frequent itemset mining was employed to extract peptide sequence features that reflect recurring amino acid combinations. Initially, the frequency of individual amino acids was calculated based on the subsets present in the peptide database. After generating an itemset list C1, amino acids meeting the support threshold was identified, and frequent itemset L1 was established. In the subsequent step, amino acid combinations that satisfied the frequency requirements were compiled. We re-selected amino acid combinations meeting the frequency criteria, creating a binomial set C2 and determining the frequent binomial set L2. Confidence analysis was applied to assess whether the strong association rules between amino acids met the required confidence thresholds. Additionally, the dataset included a fourth column representing an additional feature, and three distinct bioactive labels in the fifth, sixth, and seventh columns, respectively. The dataset was split into training and test sets, with 80% used for model training and 20% for testing.

Decision tree model training and evaluation: Decision tree models were constructed for each bioactive label, designated as model_5, model_6, and model_7. The models were trained on the training set, followed by evaluation using the test set to calculate accuracy and classification reports. Feature importance analysis was conducted to determine the contribution of each feature to model performance, with horizontal bar plots depicting the extent of feature impact on the model. To further elucidate the decision-making process, decision tree visualizations were generated for each model, providing an intuitive understanding of their classification outcomes.

1. *Molecular Dynamics Simulation*

A one-component lipid bilayer comprising 1-palmitoyl-2-oleoyl-sn-glycero-3-phosphoglycerol (POPG) was constructed using the CHARMM-GUI Membrane Builder.^[1]^ The bilayer was solvated with coarse-grained (CG) water molecules and neutralized by adding appropriate counterions to achieve electrostatic equilibrium in the presence of the engineered BPs. Each leaflet of the bilayer contained 64 lipid molecules, and periodic boundary conditions (PBC) were applied in all directions. Within the engineered BPs–bilayer system, the peptide-to-lipid (P/L) ratio was maintained at 25:64, which has been demonstrated to ensure sufficient peptide–membrane interaction without excessive aggregation. The initial z-directional separation between the BPs and the lipid bilayer was carefully optimized to prevent steric clashes and artificial perturbations arising from initial configuration artifacts, thereby ensuring a physically realistic equilibration process.

The coarse-grained parameters for POPG lipids and water molecules were generated following the standard MARTINI 2.2 parameterization scheme, while those for the designed antimicrobial peptides were obtained through the automated CHARMM-GUI Coarse-Grained Model Generator, which converts all-atom CHARMM structures to their MARTINI representations based on established mapping rules.^[2]^ In accordance with the MARTINI framework, atoms are grouped into interaction sites (“beads”), typically representing four heavy atoms or their chemical equivalents, with interaction potentials fine-tuned to reproduce thermodynamic and structural properties of the underlying atomistic system.

Given that the MARTINI model and GROMACS engine do not natively support dynamic pH control, pH-dependent simulations were implemented through a constant-pH molecular dynamics (CpHMD) approximation available in the CHARMM-GUI Constant-pH Simulator module. Specifically, prior to coarse-graining, the protonation states of titratable residues within the peptides were adjusted to reflect the target pH conditions based on their reference pKa values predicted by the CHARMM-GUI CpHMD tool. This procedure assigns static protonation states corresponding to the specified pH environment, thereby mimicking pH-dependent electrostatic and structural effects within the MARTINI representation. For systems involving BPs in the absence of lipids, additional solvent-only CpHMD simulations were conducted to examine the stability of BPs under different protonation regimes. These constant-pH simulations were generated using CHARMM-GUI, which automatically parameterizes residue charges and provides compatible GROMACS input files for subsequent equilibration and production runs.

All MD simulations were performed using GROMACS 2021.3 with a time step of 20 fs. The Berendsen thermostat and Parrinello–Rahman barostat were employed to maintain a temperature of 310 K and a pressure of 1 bar, respectively. The nonbonded cutoff was set to 1.1 nm, and the neighbor list was updated every 10 steps. Electrostatic interactions were computed using the reaction-field method, consistent with MARTINI best practices.

1. *Solid-Phase Peptide Synthesis and HPLC Retention Time*

Engineered BPs were synthesized at Sangon Biotech (Shanghai, China) using the Fmoc-based solid-phase synthesis method and purified to a level exceeding 95%, consistent with previous studies.^[3]^ The compound powder was dissolved in ultrapure water at a stock concentration of 2.56 mM and stored at −80 °C. Prior to use, the stock solution was diluted with an appropriate solvent to the desired working concentration.

1. *The preparation of Ni-HW*

Nickel acetate was prepared as solutions within a defined concentration range (0.0 – 3.2 mM) and subjected to ultrasonic treatment for a specified duration. Subsequently, the nickel acetate solution was mixed with the peptide solution in a 1:1 volume ratio and allowed to cool slowly overnight at 25 °C.

1. *Preparation of Stable Delivery Nanomotor Storage Station*

The preparation of erythrocyte membrane-derived vesicles was based on previously established protocols.^[4]^ Blood was collected from female C57BL/6 mice via orbital puncture and anticoagulated with 1.5 mg of EDTA per milliliter. The samples were then subjected to centrifugation at 1000×g for 10 min at a temperature of 4°C, facilitating the separation of plasma and leukocytes. This was followed by three meticulous washes in ice-cold 1× PBS. To isolate the erythrocytes, a hypotonic lysis procedure was conducted using a 0.25× PBS solution (prepared by diluting PBS with deionized water in a 1:3 ratio) in an ice bath for 30 min. After removing the liberated hemoglobin through centrifugation at 15,000 rpm for 5 min, the resultant light-pink cell pellet was harvested and washed twice with 1× PBS. This intricate process ultimately yielded erythrocyte ghosts, which were subsequently processed to generate membrane-derived vesicles.

NRS@RBC were meticulously engineered through an advanced emulsification process. Specifically, 6 mg of PGCL was precisely dissolved in 500 μL of acetone. This solution was subsequently amalgamated with 1 ml of a 6.4 mM Ni-HW self-assembled product. The resulting concoction was carefully transferred to a pristine Abe bottle and subjected to vigorous magnetic stirring at 1000 rpm. In a gradual manner, 1.5 mL of a 0.5% poloxamer solution was delicately introduced. The temperature was methodically elevated to 40°C, and the stirring persisted for an additional hour. The resultant emulsion was then collected and subjected to centrifugation at 14,000 rpm, followed by washing with an aqueous solution containing 5% DMSO, before being preserved at −80°C. For the erythrocyte membrane coating of NRS@RBC, 1 mL of NRS@RBC was harmoniously blended with 1 mL of vesicles derived from erythrocyte membranes. This sophisticated mixture was extruded 15 times through a 200 nm polycarbonate porous membrane using an Avanti micro-extruder. The resultant NRS@RBC were incubated overnight in 1× PBS buffer at 4°C, preparing them for subsequent applications.

1. *Characterizations based on Ultraviolet-visible*

The self-assembled HW and Ni-HW samples were separately introduced into quartz cuvettes, and their absorbance spectra were recorded across a wavelength range of 200 to 800 nm using an ultraviolet-visible spectrophotometer.

1. *1,8-ANS Fluorescence Spectroscopy to Characterize the Engineered BPs*

The self-assembly properties of engineered BPs were assessed using the 1,8-ANS fluorescent probe. A 20 μM solution of 1,8-ANS was prepared in DMF, and varying concentrations of engineered BPs (1280, 640, 320, 160, 80, 40, 20, 10, 5, 2.5, and 1.25 μM) were mixed 1:1 with the fluorescent probe and diluted to the target concentrations. Following a 15 min incubation, fluorescence intensity was measured at 10 nm intervals, with an excitation wavelength of 369 nm and an emission range of 440–550 nm.

1. *Critical Micelle Concentration (CMC) Assay*

The CMC of each engineered BPs was determined using a solvatochromic Nile red probe. A 2.5 μM Nile red solution was prepared in ethanol, and engineered BPs were mixed with the probe at a 1:1 ratio, then diluted to the desired concentrations (1280, 640, 320, 160, 80, 40, 20, 10, 5, and 2.5 μM). After 15 min of incubation, fluorescence intensity was measured at 10 nm intervals, with an excitation wavelength of 550 nm and an emission range of 600–750 nm.

1. *Dynamic Light Scattering (DLS)*

Engineered BPs were diluted to their critical micelle concentration using PBS (10 mM, pH 7.4 or 6.0) and incubated at 37°C for 2 h. The particle size distribution was then assessed via DLS. For NRS and NRS@RBC, measurements were performed directly on the prepared solutions.

1. *Zeta-Potential Assay*

The zeta potential of the engineered BPs was measured after dilution to their critical micelle concentration in PBS (10 mM, pH 7.4 or 6.0) following a 2 h incubation at 37°C. Zeta potential measurements for NRS and NRS@RBC were conducted directly on the prepared nanoparticle suspensions.

1. *Transmission Electron Microscopy (TEM) Assay*

Samples of engineered BPs, Ni-HW and NRS@RBC were prepared by depositing droplets onto carbon-coated 230 mesh copper grids. After 2 min of deposition, excess liquid was removed with filter paper, and the samples were air-dried at room temperature. Negative staining was carried out using a 1 wt% phosphotungstic acid solution, followed by further air drying for subsequent TEM analysis.

1. *Circular dichroism (CD) Spectroscopy*

The CD spectra of the engineered BPs and Ni-HW were meticulously recorded in ultrapure water, 30 mM sodium dodecyl sulfate (SDS), and 50% trifluoroethanol (TFE) at a concentration of 150 μM. Additionally, the engineered BPs underwent trypsin digestion for 4 hours at 37°C, with a final trypsin concentration of 4 mg/mL and a 1:1 volume ratio. To thoroughly assess the structural stability of the engineered BPs, comprehensive CD wavelength scans were performed across the spectrum from 195 to 250 nm. The secondary structure content was determined using the fitting method described by *Yang* and *Read*. The average residue ellipticity was calculated according to the following formula: (observed ellipticity × 1000) / (peptide concentration × optical path length × number of amino acid residues).

1. *MIC Assays Against Individual Bacterial Strains*

The bacterial strains used for the MIC assay included: *Acinetobacter baumannii*, *Klebsiella pneumoniae*, *Escherichia coli* 987P, *E. coli* 1515, *E. coli* ER2738, *E. coli* K88, *E. coli* ETEC K88, *E. coli* K99, *E. coli* UB1005, *E. coli* 25922, *E. coli* 078, *Pseudomonas aeruginosa* ATCC 27853, *P. aeruginosa* PAO1, *P. aeruginosa* CICC 21625, swine paratyphoid 021493, *Salmonella Enteritidis* CVCC 3377, *S. pullorum* C7913, *S. Typhimurium* ATCC 14028, *S. Typhimurium* C7731, *Listeria monocytogenes* CGMCC 1.1075, *Bacillus cereus* CGMCC 1.932, *Staphylococcus aureus* ATCC 29213, *S. aureus* 43300, *Staphylococcus epidermidis* ATCC 12228, *Enterococcus faecalis* ATCC 29212, *S. aureus* 25923, *Lactobacillus rhamnosus* 8014, and *L. rhamnosus* 7469. Fungal strains included: *Candida albicans* CGMCC 2.2086, *C. albicans* SP3931, *C. albicans* SP3903, *C. albicans* SP3876, *C. albicans* 56452, *C. albicans* 56214, *C. albicans* 14936, *C. albicans* 17546, and *Candida parapsilosis* CGMCC 2.3989. Bacterial and fungal strains were cultured in MHB and YPD media, respectively. For *L. rhamnosus* strains 8014 and 7469, M17 medium was used.

The absorbance of each strain during growth was measured at OD_600nm_ using a UV spectrophotometer, while OD_492nm_ was used for drug sensitivity testing with an enzyme reader. Bacterial cultures were inoculated the night before the MIC experiment. On the day of the assay, engineered BPs solutions were thawed and transferred to a culture chamber. Engineered BPs were serially diluted in BSA solution across 96-well plates (50 µl) with final concentrations ranging from 64 to 0 μM. Subsequently, 50 µL of pathogenic microorganisms diluted 1000-fold in the growth medium was added to each well. Plates were incubated at 37°C for 16-18 h, after which bacterial growth was assessed by measuring OD_492nm_. The MIC was defined as the lowest concentration of engineered BPs that inhibited >99% growth.

1. *In Vitro Resistance Acquisition Assays*

ETEC K88 cells were continuously cultured for 10 days in the presence of NRS@RBC and ciprofloxacin at sub-inhibitory concentrations. Briefly, ETEC K88 was cultivated in LB medium supplemented with either NRS@RBC or ciprofloxacin. Every 24 h, bacterial cultures from the highest concentration that supported visible growth were selected, and the inoculation process was repeated. Additionally, the MIC value was reassessed every 24 h to monitor potential changes in antimicrobial susceptibility.

1. *Hemolytic Activity Assay*

The hemolytic activity of engineered BPs, Ni-HW, polluted Ni-HW and ETEC K88 was evaluated by monitoring hemoglobin release from RBCs obtained from a healthy donor at Northeast Agricultural University Hospital. The study was approved by the Ethics Committee of the hospital, and all procedures followed established guidelines and regulations. In brief, healthy human RBCs were collected in a polycarbonate tube containing EDTA as an anticoagulant, and centrifuged at 1000 g at 4°C for 10 min. The RBCs were washed three times and re-suspended in PBS (10 mM, pH 7.4). A 50 μL RBC suspension was incubated in PBS (10 mM, pH 7.4) at 37°C for 1 h, followed by centrifugation at 1000 g at 4°C for 10 min. The hemolytic activity was quantified by measuring the absorbance of the supernatant at 570 nm using a microplate reader. PBS (10 mM, pH 7.4) and 0.1% Triton X-100 were used as negative and positive controls, respectively.

1. *Platelet Aggregation Assay*

First, collect fresh mouse blood into anticoagulant tubes containing sodium citrate and centrifuge at 200 × g for 8 min. Carefully aspirate the supernatant and centrifuge it at 1028 × g for 12 min. Resuspend the resulting pellet in an equal volume of HEPES buffer to obtain a platelet suspension. Mix the platelet suspension with the drug at a 1:1 volume ratio and incubate at 37 °C on a shaker at 30 rpm for 15 min. Subsequently, apply 20 μL of the treated platelet suspension onto the surface of a glass slide for adhesion incubation. After an additional 15-min incubation, fix the platelets with 4% paraformaldehyde for 10 min. Wash the glass slide gently with fresh HEPES buffer to remove residual fixative. Following air drying, stain the platelets with 100 μL of Dil fluorescent dye in the dark for 20 min, and then remove unbound dye using HEPES buffer. Finally, observe platelet adhesion under a fluorescence inverted microscope and perform quantitative image analysis using ImageJ software.

1. *Cytotoxicity Assay*

Cell lines including RAW264.7 (mouse macrophages), IPEC-J2 (porcine intestinal epithelial cells), and PK15 (porcine kidney cells) were cultured in RPMI-1640, DMEM high-glucose, and standard DMEM media, respectively, supplemented with 100 U/mL penicillin, 0.1 mg/mL streptomycin, and 10% fetal bovine serum. Cells were incubated at 37°C in a humidified atmosphere of 5% CO_2_ and 95% air. The cytotoxicity of engineered BPs was assessed using an MTT cell viability assay. Briefly, cells were seeded into 96-well plates at a density of 1 × 10^5^ cells per well. Engineered BPs were serially diluted and incubated with the cells for 4 h. Following incubation, MTT reagent was added to each well and further incubated for 4 h. After discarding the supernatant, formazan crystals were dissolved in DMSO, and absorbance was measured at 570 nm using a microplate reader. The culture medium without cells served as a blank, and blank absorbance values were subtracted from those of each sample. Cell viability was calculated relative to control values.

1. *Sensitivity Assays*

A solution containing 150 mM NaCl, 4.5 mM KCl, 2 mM CaCl₂, 1 mM MgCl₂, 8 μM ZnCl₂, 4 μM FeCl₃, and 6 μM NH₄Cl was prepared. Engineered BPs were subsequently mixed with the prepared ionic solution at varying concentrations, followed by determination of their MIC. The thermal stability of the engineered BPs was assessed by incubating them at 100°C and 80°C for 30 min. To evaluate pH stability, engineered BPs were incubated for 1 h in PBS solutions with different pH values prior to performing MIC assays. Serum stability was assessed by incubating engineered BPs with varying concentrations of serum for 1 h, followed by MIC determination. Protease stability was examined by incubating engineered BPs with trypsin, protease, pepsin, papain, and proteinase K at 37°C for 30 min, with each protease at a final concentration of 4 mg/mL, after which MIC values were measured.

1. *Molecular Docking Assay*

The trypsin sequence was obtained from the UniProt database. Given the length of the peptide sequence, traditional peptide-protein docking methods were unsuitable. Instead, AlphaFold v2.3.2 multimer modeling was employed to input both the protein and peptide sequences, constructing the optimal binding conFigureuration via deep learning-based simulation. The active sites of the peptides and trypsin were further characterized using MM-GBSA calculations, where MM-GBSA ΔG_bind approximated the binding free energy of peptide-protein complexes. Lower binding free energy values indicate greater stability in peptide-protein interactions. Three peptide-protein complexes were color-labeled, and 3D surface representations were generated for spatial visualization. Additionally, the Protein Interaction Analysis module was utilized to pinpoint the specific regions involved in peptide-protein binding.

To refine the binding conFigureurations of protein-peptide complexes, traditional molecular dynamics simulations were conducted using the Desmond software suite. Protein and small molecule parameters were meticulously defined using the advanced OPLS4 force field, while water molecules were modeled with the SPCE model for enhanced accuracy. The complexes were enveloped in a cubic water box, and charge neutrality was achieved by introducing 0.150 mM chloride and sodium ions. System energy minimization was performed through an exhaustive steepest descent method over 50,000 steps. Subsequently, NVT and NPT equilibration phases were executed, each comprising an additional 50,000 steps with heavy atom positions restrained to ensure stability. The simulation conditions were maintained at a temperature of 300 K and a pressure of 1 bar. Following equilibration, an unconstrained molecular dynamics simulation was carried out for 100 ns, and the resulting interactions were meticulously analyzed using Maestro 2023.

1. *Proteolytic Resistance Assays*

For the protease stability assays, engineered BPs (2.56 mM) were incubated in protease solutions (8 mg/mL) at 37°C for varying durations (1 h, 2 h, 4 h, and 8 h). The controls consisted of engineered BPs alone and protease alone. After the incubation period, all samples were subjected to heating at 100°C for 30 min, followed by centrifugation at 13,000 g for 30 min to pellet the protease. The resulting supernatants were then evaluated using 16.5% Tricine-SDS-PAGE and HPLC.

1. *Exploration of Intestinal Colonization*

To assess the tissue colonization of ETEC K88 in mice, a pseudo-sterile mouse model was established. Mice were given free access to drinking water containing antibiotics (2.5 mg/mL streptomycin, 1 mg/mL ampicillin, 0.5 mg/mL vancomycin, and 0.5 mg/mL metronidazole) for 7 d to clear intestinal flora. Following this, mice were administered ETEC K88 (OD_600nm_ = 0.8) for four consecutive days before euthanasia. Blood, liver, spleen, lungs, kidneys, feces, and samples from the duodenum, jejunum, and ileum were collected. The samples were serially diluted in saline and plated on MHA, followed by incubation at 37°C for 16–18 h. CFU per mL were then calculated.

1. *Intestinal Bacterial Injury Model in Mice*

Female C57BL/6 mice (6–8 weeks old) were also procured from Liaoning Changsheng Biotechnology Co., Ltd., fed a standard diet, and subjected to experimental intestinal bacterial damage over a 7 d period. Briefly, the mice received a daily administration of 20 mg/kg HW saline suspension at 20:00 for 7 consecutive days. From day 4, the mice were administered an ETEC K88 saline suspension (OD_600nm_ = 0.8) each morning for 4 consecutive days. On the 8 d, the mice were anesthetized with ether, and blood was collected via the orbital sinus to isolate serum. Mice were then sacrificed, and the jejunal tissues were harvested for further analysis.

1. *Intestinal Bacterial Injury Model in Weaned Piglet*

In a parallel experiment, 24 weaned piglets with an average body weight of 10 ± 2 kg (three-way crossbreed: Duroc × Large White × Landrace) were selected from a commercial farm. The piglets were housed in stainless steel metabolic cages equipped with automatic feeders and water spouts, allowing ad libitum access to food and water. After a 7 d acclimatization period, the pigs were randomly assigned to four experimental groups, with each animal housed individually. The groups were as follows: Control (-) (sterile saline), ETEC K88 (+) (ETEC K88 saline suspension, OD_600nm_ = 1.0), HW (5 mg/kg HW saline solution containing 1% sucrose), and Prve Gro (combined treatment of drug and ETEC K88, following the same treatment protocol as in mice). Forced feeding was conducted over a 7 d period, and all piglets were euthanized on the 8th day. Tissue samples were immediately harvested post-slaughter, rinsed, and stored at -80°C until analysis.

1. *Serum Biochemical Analysis*

Serum biochemical indices, including alanine aminotransferase (ALT), aspartate aminotransferase (AST), lactate dehydrogenase (LDH), total protein (TP), and albumin (ALB), were measured via colorimetric methods using an automated biochemical analyzer (FULLY, Italy) according to the manufacturer’s instructions.

1. *Histological Staining*

The tissue samples were fixed in formalin, embedded in paraffin, and stained with hematoxylin and eosin (H&E). The stained slides were visualized and imaged using an American Moticam 3000 photomicrographic system. An independent and blinded researcher conducted the histopathological assessments.

1. *T-SOD Activity, GSH and MDA Detection Assay*

T-SOD activity was determined using the Total Superoxide Dismutase Assay Kit with WST-8 (Beyotime). Intestinal tissues were homogenized in an ice bath, with 100 μL of SOD sample preparation solution added to every 10 mg of tissue. Absorbance at 450 nm was measured according to the manufacturer’s protocol.

Tissue GSH levels were assessed using the GSH and GSSG Assay Kit (Beyotime). The tissues were flash-frozen in liquid nitrogen, ground into powder, and the total GSH content was calculated according to the provided instructions.

MDA levels, indicative of lipid peroxidation, were quantified using the Lipid Peroxidation MDA Assay Kit (Beyotime). Test tissues were lysed using Western and IP cell lysates, and MDA content in the sample was determined following the manufacturer’s protocol.

1. *Immunofluorescence Staining Assay*

RAW264.7 cells were seeded in 24-well plates (50,000 cells per well), with subsequent treatments initiated the following day. Cells were stimulated with ETEC K88 at OD_600nm_ = 0.4 for 1 h, 2 h, and 4 h. HW, prepared under CMC conditions, was added to the cell culture medium 1 h prior to stimulation. For paraffin-embedded jejunum or colon tissue sections, dewaxing was performed in xylene for 5 min, followed by two additional xylene treatments. Sections were sequentially immersed in anhydrous ethanol twice for 5 min, in 90% ethanol twice for 5 min, and in 70% ethanol once for 5 min. After immersion in distilled water twice for 5 min, the slices were subjected to antigen retrieval by heating 10 mM sodium citrate (pH 6.0) at 95°C for 12 min, followed by gradual cooling to room temperature. The cells or sections were then fixed with immunostaining fixative and washed twice with immunostaining wash buffer. Immunostaining blocker was applied for 60 min. Subsequently, GPX4 rabbit polyclonal antibody and FITC/Cy3-labeled goat anti-rabbit IgG (H+L) were utilized for primary and secondary antibody detection, respectively. Imaging was performed using super-resolution microscopy.

1. *Cell Viability Assay*

For cell viability assays, RAW264.7 cells were seeded in 96-well plates and allowed to adhere overnight. On the following day, cells were treated with cell death inducers. Prophylactic compounds were added 1 h prior to treatment. Cell viability was assessed 4 h after treatment with erastin, RSL3, TNF-α + SM-164, or TSZ using the Cell Counting Kit-8 (CCK-8). Cell viability was expressed as a percentage relative to the control sample, which was set at 100%.

1. *ROS Detection* *Assay*

The ROS content in RAW264.7 cells was measured using the Reactive Oxygen Species Assay Kit (Beyotime). DCFH-DA was diluted in serum-free medium at a ratio of 1:1000 to a final concentration of 10 μM. Cells were suspended in the diluted DCFH-DA solution and incubated at 37°C for 20 min, with periodic mixing every 3–5 min to ensure adequate contact between the probe and cells. After washing the cells three times with serum-free medium, they were stimulated with the test compound and ETEC K88. Fluorescence intensity was measured at 1 h, 2 h, and 4 h post-stimulation, using an excitation wavelength of 488 nm and an emission wavelength of 525 nm.

1. *Iron-Chelating Activity Assay*

To determine the iron content in RAW264.7 cells, the Serum Ferri Ion Content Assay Kit (Solarbio) was used to measure total iron, Fe^3+^, and Fe^2+^ levels according to the manufacturer’s protocol.

1. *Lipid peroxides Detection* *Assay*

Lipid peroxide levels in RAW264.7 cells were quantified using the Lipid Peroxide (LPO) Content Assay Kit (Solarbio) and Liperfluo (DOJINDO). The LPO content was calculated according to the provided protocol and expressed as the concentration of lipid peroxides in the sample.

1. *BODIPY 581/591 C11 Staining*

For lipid peroxidation assays, RAW264.7 cells (50,000 cells per well) were seeded in 6-well plates on the day prior to the experiment. On the second day, cells were treated with ETEC K88 (OD_600nm_ = 0.4) for 1 h, 2 h, and 4 h, with HW (CMC) added 1 h prior to ETEC K88 treatment. After incubation with ETEC K88, cells were incubated with 1.5 μM BODIPY 581/591 C11 at 37°C for 30 min. The cells were then trypsinized, resuspended in 300 µL HBSS, filtered through a 40 µm cell filter, and imaged using super-resolution microscopy.

1. *LPS Dissipation Assay*

ETEC K88 was incubated with 0.25 mM EDTA, and CFUs were measured following the same protocol as the CFU counting assay. All tests were performed in triplicate to ensure reproducibility.

1. *LPS binding Assay*

LPS binding assays were conducted as previously described.^[5]^ Briefly, 50 μg/mL LPS and 5 μg/mL BC were incubated in 50 mM Tris buffer (pH 7.4) at 37°C for 4 h to facilitate LPS-BC binding. LPS-bound probes were then mixed with membrane material. Wells containing only LPS probes were used as negative controls to determine background fluorescence. To assess membrane recycling function, samples were collected at 90 minutes of incubation, and 0.2% Triton X-100 was added prior to continuing the assay. Fluorescence intensity was measured using an enzyme-labeled reader, with excitation and emission wavelengths set at 580 nm and 620 nm, respectively.

1. *Fluorescence Inverted Microscope Imaging Assay*

To evaluate the integrity of the ETEC K88 cell membrane, bacterial cells were labeled with FITC-conjugated BPs and examined using fluorescence microscopy. ETEC K88 bacteria in the logarithmic growth phase were collected by centrifugation, then re-suspended in PBS (10 mM, pH 7.4) and adjusted to an OD_600nm_ of 0.1. The bacterial solution was combined at a 1:1 ratio with FITC-BPs (to achieve a final concentration at CMC) in a 96-well plate and incubated at 37°C for 2 h. Fluorescence microscopy images were obtained using an inverted microscope, with unlabeled samples used as negative controls. It is important to note that these bacteria express mCherry as an inherent marker protein.

1. *Flow Cytometry Analysis Assay*

To further examine bacterial membrane disruption, flow cytometry was utilized. ETEC K88 cells in the logarithmic growth phase were adjusted to an OD_600nm_ of 0.1 by dilution in PBS (10 mM, pH 7.4). The engineered BPs were prepared at concentrations twice their CMC through serial dilution. A volume of 100 μL from the bacterial suspension was combined with 100 μL of the BPs solution and incubated at 37°C for 60 min. PI was then added to achieve a final concentration of 20 μg/mL, followed by an additional incubation period of 30 min at 37°C. Fluorescence intensity was assessed using flow cytometry, with control samples containing no BPs.

1. *SEM and TEM Observation of the Morphological Changes of Bacteria*

For SEM analysis, ETEC K88 cells in the logarithmic growth phase were suspended in PBS (10 mM, pH 7.4) to achieve an OD_600nm_ of 0.2. The bacterial suspension was then incubated with engineered BPs at a concentration double the CMC for 2 h at 37°C. Following this treatment, the cells were collected via centrifugation at 12,000 rpm, and the supernatant was removed. The cell pellet was fixed using 2.5% glutaraldehyde and kept overnight at 4°C. Subsequently, the cells underwent sequential dehydration using increasing concentrations of ethanol (50%, 70%, 90%, and 100%), followed by treatment with 50% and 100% tert-butanol. Samples were dried using liquid CO_2_, sputter-coated with palladium, and visualized by SEM. For TEM, cells were adsorbed onto copper grids for 30 s, stained with phosphotungstic acid for 8 s, and imaged by TEM.

1. *Outer Membrane Permeability Assay*

Logarithmic-phase bacterial cells were collected and suspended in 5 mM HEPES buffer (with 5 mM glucose, pH 7.4) until the OD_600nm_ reached 0.2. A volume of 2 mL of this bacterial suspension was combined with 10 μM N-phenyl-1-naphthylamine (NPN) and then exposed to different concentrations of engineered BPs. Negative controls, which included bacterial cells and NPN, served as the baseline for 0% permeability, whereas positive controls, consisting of bacterial cells, NPN, and 10 µg/mL polymyxin B, represented 100% permeability. Each experiment was conducted independently in triplicate. The cell wall permeability was assessed by measuring fluorescence intensity, with excitation at 350 nm and emission at 420 nm. Permeability was determined using the formula: NPN uptake (%) = [(F_obs_ − F_o_)/(F_100_− F_o_)] × 100%, where F_obs_ is the fluorescence intensity following treatment with engineered BPs, and F_o_ and F_100_ are the fluorescence intensities of the negative and positive controls, respectively.

1. *Plasma Membrane Depolarization Assay*

Bacterial cells during their logarithmic growth phase were harvested and suspended in 5 mM HEPES buffer (which included 20 mM glucose, pH adjusted to 7.4) until the OD_600nm_ reached 0.05. Following a 60 min incubation with 0.4 μM Disc3-5, these cells were exposed to 100 mM potassium ions (K⁺) for an additional 30 min. Subsequently, engineered BPs were introduced into the suspension at a final concentration double that of the CMC, within a 24-well plate setup. Control wells, containing only bacteria and dye, were used for background correction. Changes in fluorescence intensity were tracked using a plate reader, employing excitation and emission wavelengths of 622 nm and 670 nm, respectively.

1. *Inner Membrane Permeability Assay*

To evaluate intimal permeability, bacterial cultures grew in MHB containing 2% lactose at 37°C until they reached the mid-exponential growth phase. The cells were subsequently collected and resuspended to an OD_600nm_ of 0.05 in a buffer solution composed of 5 mM HEPES (pH 7.4), 20 mM glucose, and 1.5 mM ONPG. Equal volumes of this bacterial suspension and varying concentrations of engineered BPs (from 2 μM to 64 μM) were combined in a sterile 96-well plate and incubated at 37°C. The OD_420nm_, indicative of ONPG uptake, was recorded at 3 min intervals for a total duration of 120 min, allowing for the analysis of intimal permeability.

1. *Determination of Hydrogen Peroxide Decomposition and Oxygen Evolution*

NRS, NRS@RBC, and RBC MEM were added to a 10 mM hydrogen peroxide solution at room temperature. The hydrogen peroxide concentration was subsequently determined using a hydrogen peroxide detection kit (Solarbio). Dissolved oxygen levels were measured using a dissolved oxygen meter. In a separate experiment, NRS, NRS@RBC, and RBC MEM were added to a 10 mM hydrogen peroxide solution at room temperature, and the oxygen concentration was recorded within 10 minutes using the oxygen probe of the dissolved oxygen meter.

1. *Assessment of Mitochondrial Membrane Potential in Cells*

In brief, IPEC-J2 cells were first pretreated with ETEC K88 (OD_600nm_ = 0.4), and then cultured with HW, NRS, NRS@RBC and RBC MEM respectively. Subsequently, the cells were incubated with JC-1 reagent for 20 min and then photographed under a fluorescence inverted microscope.

1. *Determination of Endotoxin Content*

The endotoxin residue in the samples was quantified using the LAL assay. LPS (10 ng) was individually mixed with NRS, NRS@RBC, RBC MEM, gentamicin, gentamicin@RBC, polymyxin B, and polymyxin B@RBC, followed by incubation at 37 °C for 2 h. Subsequently, the mixtures were centrifuged at 13,000 × g at room temperature for 20 min, and the supernatants were collected for endotoxin analysis. Endotoxin levels were then measured using an LAL test kit (Beyotime) according to the manufacturer's instructions.

1. *The intestinal barrier permeability study of NRS@RBC*

To evaluate the capability of functionalized nanoparticles to penetrate the mucus layer, a Transwell system was employed. IPEC-J2 cells or mouse intestinal crypt-derived organoids were seeded in the lower chamber of the Transwell plate. Nanoparticles were labeled with Cy5, and mucus (with or without 2 mM H_2_O_2_) was stained using FITC-conjugated wheat germ agglutinin. The nanoparticles were introduced into the upper chamber, followed by incubation at 37°C and 50 rpm in an orbital shaker for 30 min. Subsequently, the cells or organoids were visualized under a fluorescence microscope. Confocal laser scanning microscopy (CLSM) z-stack imaging was performed at 1-micron intervals to assess nanoparticle distribution. Cy5 served as a fluorescent tracer encapsulated within the nanoparticles. In in vivo experiments, 200 μL of NRS@RBC was administered orally to healthy and colitis-induced mice, while 100 μL of FITC-conjugated wheat germ agglutinin was delivered via rectal enema. Following biopsy, whole colon tissue samples were immediately mounted on glass slides and examined under a fluorescence microscope.

1. *Pathogenic colitis model*

Female C57BL/6 mice were randomly assigned to eighteen experimental groups (n = 8 per group), with nine groups designated for the acute phase study and the remaining nine for the chronic phase study. The group designations were as follows: (i) Blank group, (ii) Model group, (iii) NRS group, (iv) NRS@RBC group, (v) RBC MEM group, (vi) Gentamicin group, (vii) Gentamicin@RBC group, (viii) Polymyxin B group, and (ix) Polymyxin B@RBC group. Mice in the Blank group served as the healthy control. For induction of infection, mice in all other groups were orally gavaged with ETEC K88 (10^9^ CFU) for the initial three days (acute phase) or five days (chronic phase). This was followed by treatment with the respective therapeutic agents for four days (acute phase) or nine days (chronic phase). Body weight changes were monitored throughout the experimental period. At the conclusion of the study, all animals were anesthetized, and blood samples were collected via orbital enucleation for serum isolation. Colon tissues and major organs were harvested, homogenized, and serially diluted before being uniformly spread onto MHA medium. The plates were incubated at 37 °C for 12–16 h, after which colony counts were recorded. For histopathological analysis, colon Swiss roll sections were prepared and stained with H&E as well as AB/PAS. The expression levels of tight junction proteins, including ZO-1 and Claudin-1, were assessed using immunofluorescence staining. All stained tissue sections were examined under a fluorescence microscope.

1. *In vivo fluorescence imaging*

The healthy female C57BL/6 mice were orally administered normal saline or Cy5-labeled NRS@RBC (5 mg/kg) via gavage. After 3 h, whole-body fluorescence imaging was performed utilizing an *in vivo* spectral imaging system (IVIS Spectrum). Upon completion of the imaging procedure, the mice were humanely euthanized, and their entire gastrointestinal tracts were harvested for subsequent fluorescence imaging analysis.

1. *Intestinal microbiota analysis*

The colonic contents from each mouse were collected individually and stored at -80 °C until further processing. Total DNA was extracted from the colonic contents using a commercial DNA extraction kit (E.Z.N.A. Stool DNA Kit, Omega Biotek). The purity of the extracted DNA was assessed using a NanoDrop spectrophotometer, with acceptable A260/280 ratios ranging from 1.8 to 2.0. Subsequently, the integrity of the nucleic acid samples was evaluated by agarose gel electrophoresis. The V4 hypervariable region of the bacterial 16S rRNA gene was amplified on the LC-Bio platform (Hangzhou, China) using the primer pair 515F (5'-GTGCCAGCMGCCGCGG-3') and 806R (5'-GGACTACHVGGGTWTCTAAT-3'). Operational taxonomic units (OTUs) were then clustered using UPARSE software (version 7.1). Taxonomic classification of the representative OTU sequences was performed using the RDP Classifier algorithm.

1. *ETEC K88 -induced colitis in mice*

Female C57BL/6 mice were randomly assigned to four experimental groups (n = 6 per group): (i) Blank group, (ii) Model group, (iii) NRS@RBC group, and (iv) Ciprofloxacin group. On day 0, all mice received intraperitoneal injections of ciprofloxacin (1 mg/kg). On day 1, mice in all groups were orally gavaged with ETEC K88 (10^9^ CFU/mL). From day 2 to day 4, mice in the NRS@RBC and Ciprofloxacin groups were orally administered NRS@RBC or ciprofloxacin, respectively, once daily. Fecal samples were collected throughout this period. The fecal samples were homogenized in physiological saline, filtered, and serially diluted before being evenly spread onto MHA medium. On day 5, the mice were euthanized, and colon contents and tissues were collected and homogenized. The resulting homogenates were plated onto MHA medium using the same procedure. Following static incubation at 37 °C for 12–18 h, colony-forming units (CFUs) were enumerated.

References

[1] a) S. Jo, T. Kim, V. G. Iyer, W. Im, Software news and updates - CHARNIM-GUI: A web-based grraphical user interface for CHARMM. *Journal of Computational Chemistry* **2008**, *29* (11), 1859, <https://doi.org/10.1002/jcc.20945>; b) Y. Qi, H. I. Ingolfsson, X. Cheng, J. Lee, S. J. Marrink, W. Im, CHARMM-GUI Martini Maker for Coarse-Grained Simulations with the Martini Force Field. *Journal of Chemical Theory and Computation* **2015**, *11* (9), 4486, <https://doi.org/10.1021/acs.jctc.5b00513>.

[2] a) L. Monticelli, S. K. Kandasamy, X. Periole, R. G. Larson, D. P. Tieleman, S.-J. Marrink, The MARTINI coarse-grained force field: Extension to proteins. *Journal of Chemical Theory and Computation* **2008**, *4* (5), 819, <https://doi.org/10.1021/ct700324x>; b) D. H. de Jong, G. Singh, W. F. D. Bennett, C. Arnarez, T. A. Wassenaar, L. V. Schafer, X. Periole, D. P. Tieleman, S. J. Marrink, Improved Parameters for the Martini Coarse-Grained Protein Force Field. *Journal of Chemical Theory and Computation* **2013**, *9* (1), 687, <https://doi.org/10.1021/ct300646g>.

[3] C. Shao, Y. Zhu, Q. Jian, Z. Lai, P. Tan, G. Li, A. Shan, Cross-Strand Interaction, Central Bending, and Sequence Pattern Act as Biomodulators of Simplified β-Hairpin Antimicrobial Amphiphiles. *Small* **2021**, *17* (7), <https://doi.org/10.1002/smll.202003899>.

[4] L. Rao, L.-L. Bu, J.-H. Xu, B. Cai, G.-T. Yu, X. Yu, Z. He, Q. Huang, A. Li, S.-S. Guo, W.-F. Zhang, W. Liu, Z.-J. Sun, H. Wang, T.-H. Wang, X.-Z. Zhao, Red Blood Cell Membrane as a Biomimetic Nanocoating for Prolonged Circulation Time and Reduced Accelerated Blood Clearance. *Small* **2015**, *11* (46), 6225, <https://doi.org/10.1002/smll.201502388>.

[5] a) C. Shao, Y. Wang, G. Li, H. Guan, Y. Zhu, L. Zhang, N. Dong, A. Shan, Novel design of simplified β-hairpin antimicrobial peptide as a potential food preservative based on Trp-pocket backbone. *Food Chemistry* **2024**, *448*, <https://doi.org/10.1016/j.foodchem.2024.139128>; b) Y. Fang, Y. Zhu, L. Li, Z. Lai, N. Dong, A. Shan, Biomaterial-Interrelated Bacterial Sweeper: Simplified Self-Assembled Octapeptides with Double-Layered Trp Zipper Induces Membrane Destabilization and Bacterial Apoptosis-Like Death. *Small Methods* **2021**, *5* (12), <https://doi.org/10.1002/smtd.202101304>.


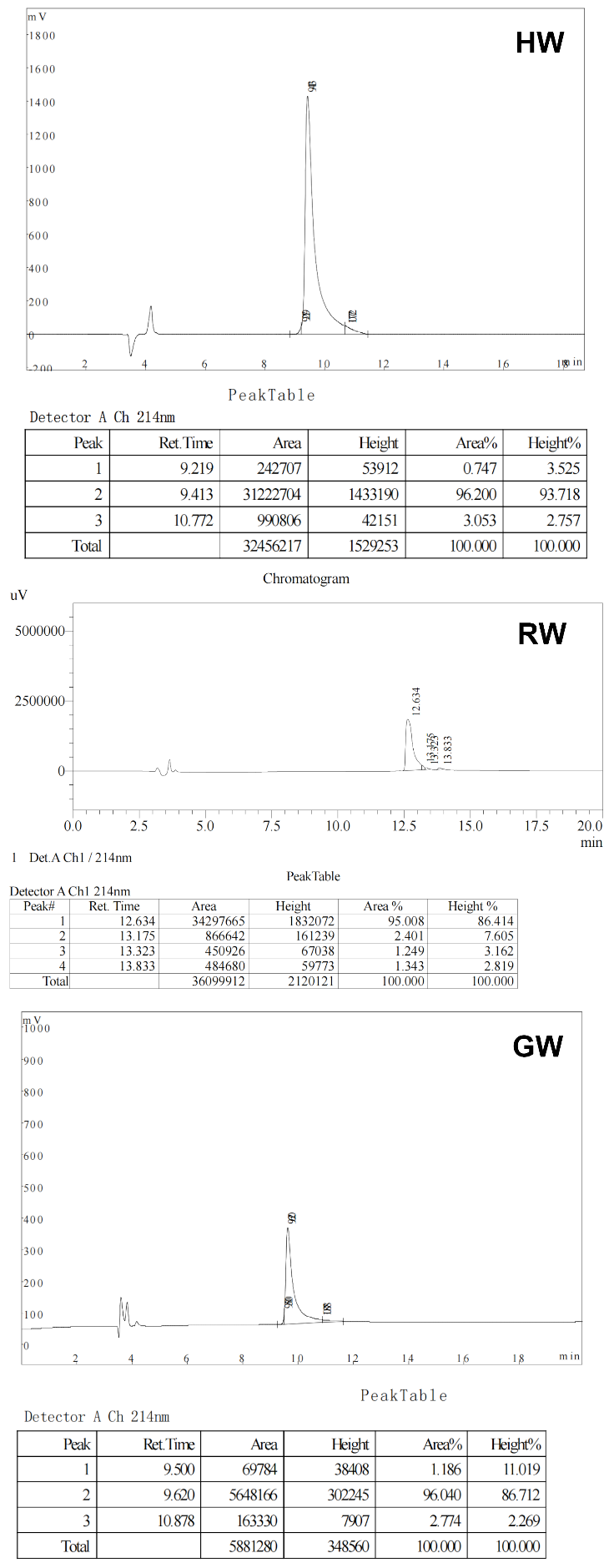


# Figure S1. High-performance liquid chromatography (HPLC) spectra of the BPs.


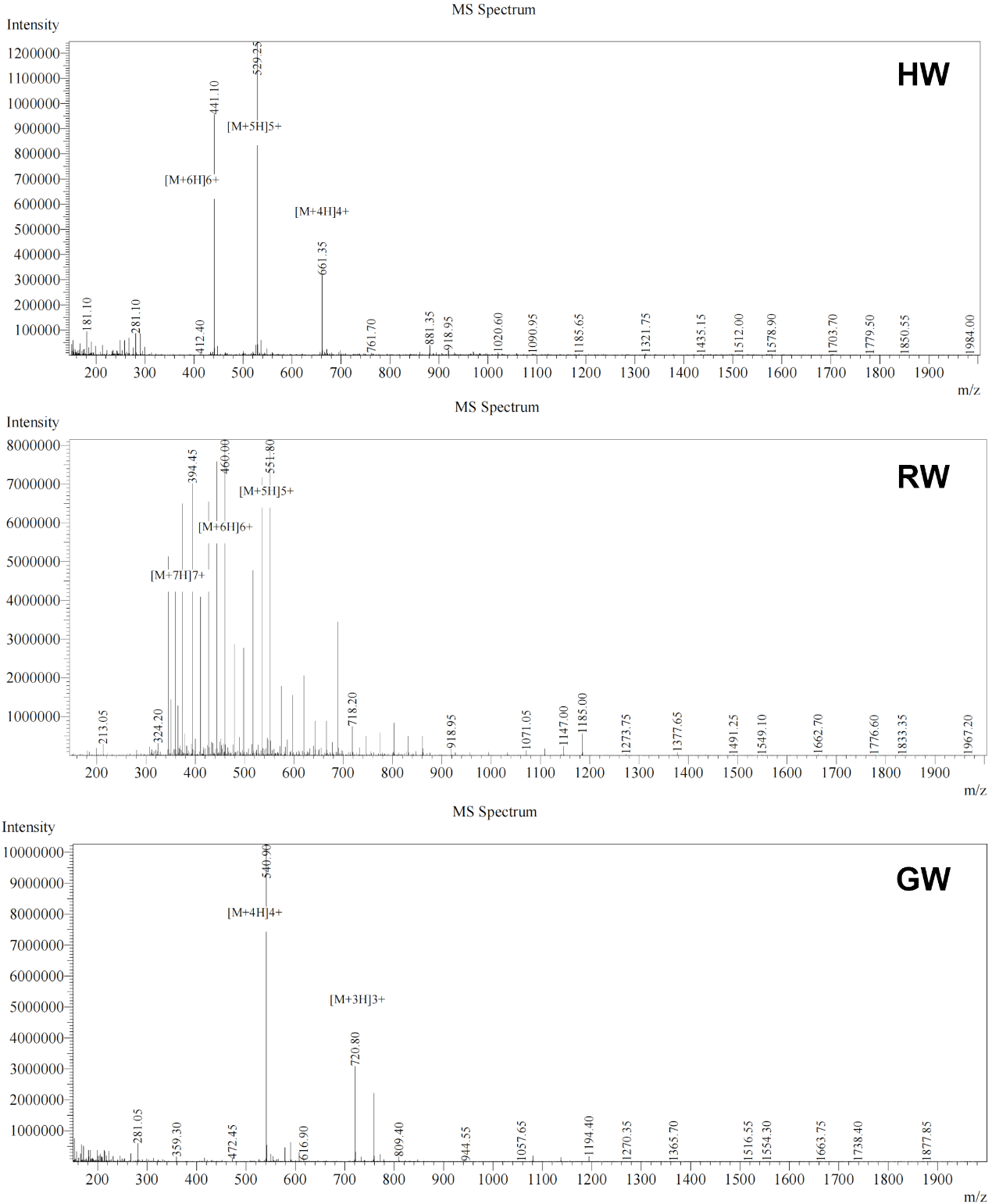


# Figure S2. Matrix-assisted laser desorption/ionization time-of-flight mass spectrometry (MALDI-TOF MS) spectra of the BPs.

#
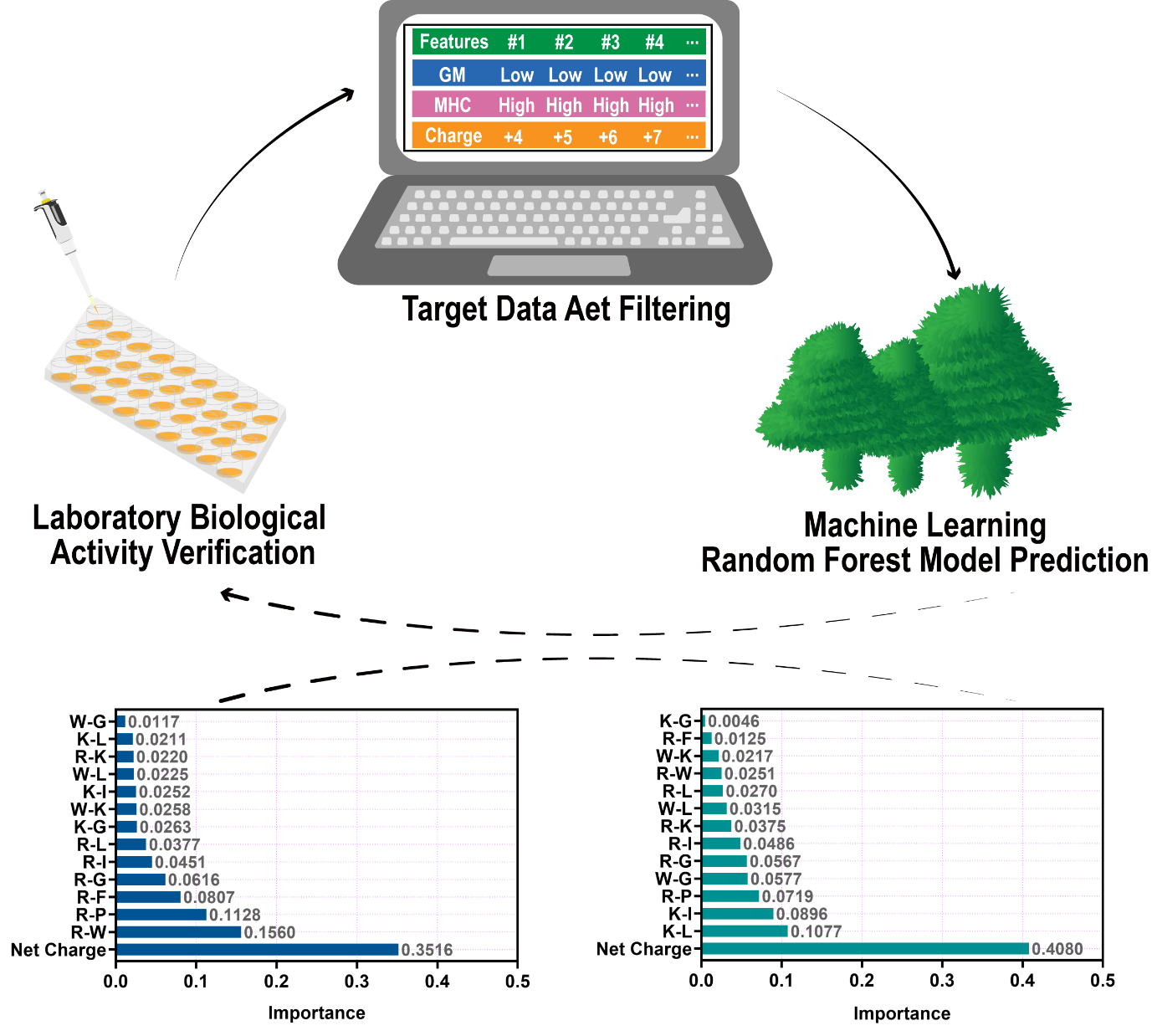


# Figure S3. Schematic representation of filtering suitable motifs using machine learning algorithms. A random forest model identified biologically important motifs from 188 highly cell-selective BPs. The +4 positive charge contributed 35.16% to resistance against Gram-negative bacteria and 40.80% to biocompatibility. Motifs RW (15.60%) and RP (11.28%), which show strong activity (0–8 GM value) against Gram-negative bacteria, were combined with motif RW (25.08%), which has high biocompatibility.


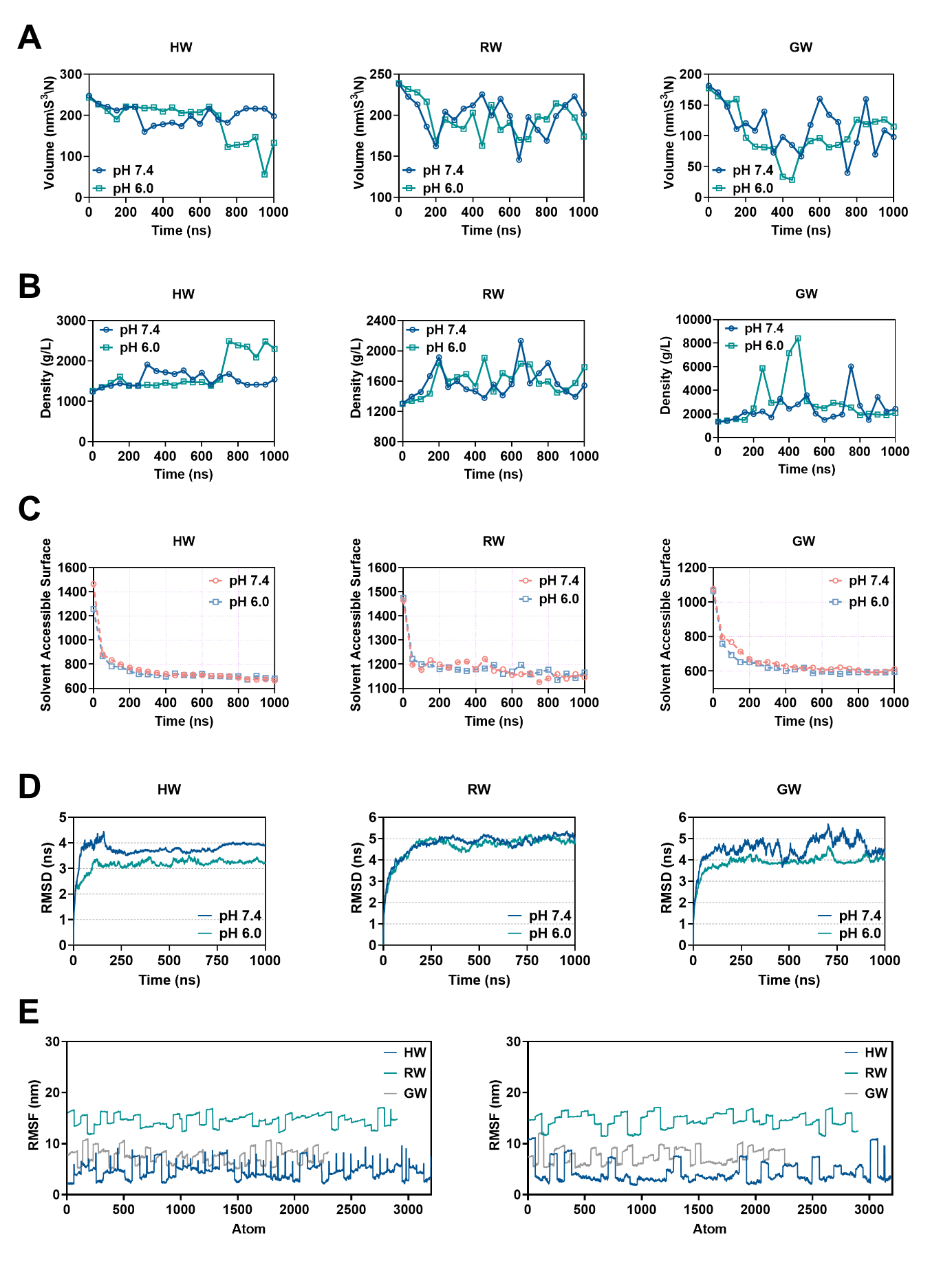


**Figure S4.** **The molecular backbone structural properties of BPs following GROMACS simulation in aqueous solution.** Variations in (A) volume and (B) density; (C) Solvent-

accessible surface area (SASA); The change of (D) root mean square deviation and (E) root mean square fluctuation.


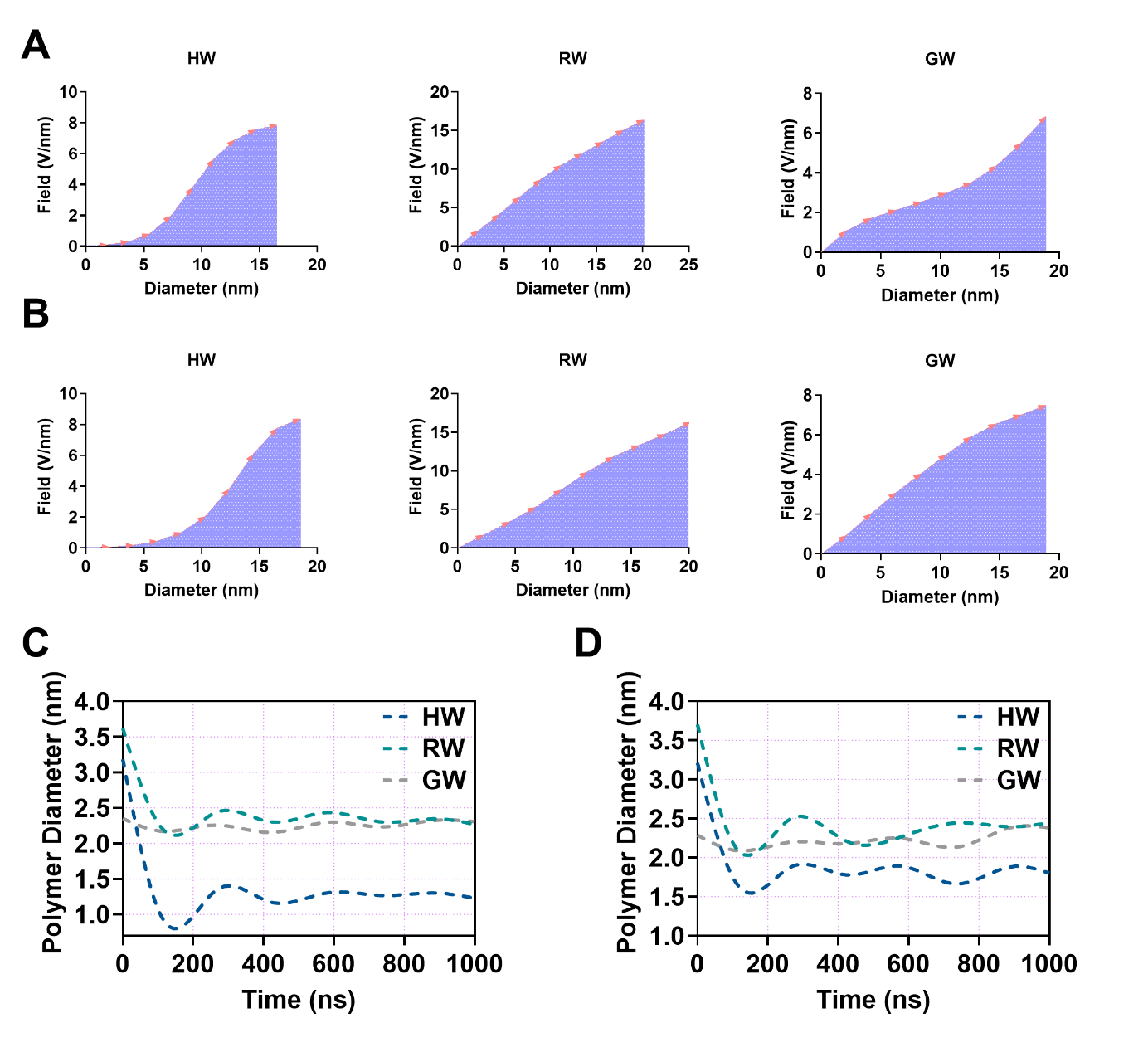


**Figure S5. The polymer spatial properties of BPs following GROMACS simulation in aqueous solution.** Overall potential difference of BPs in pH 6.0 (A) and pH 7.4 (B) environment. The polymer diameter changes of BPs at pH 6.0 (C) and 7.4 (D), respectively.


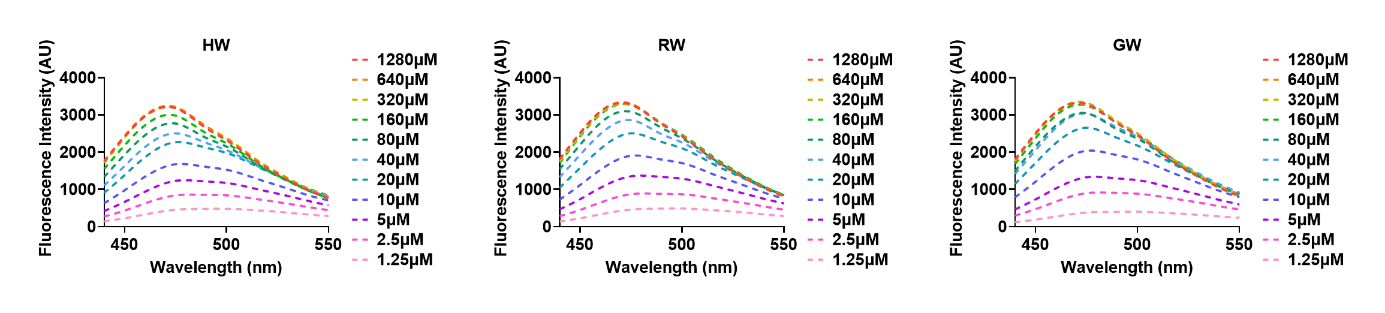


# Figure S6. The fluorescence intensity of BPs at different concentrations was measured by 1, 8-ANS probe.


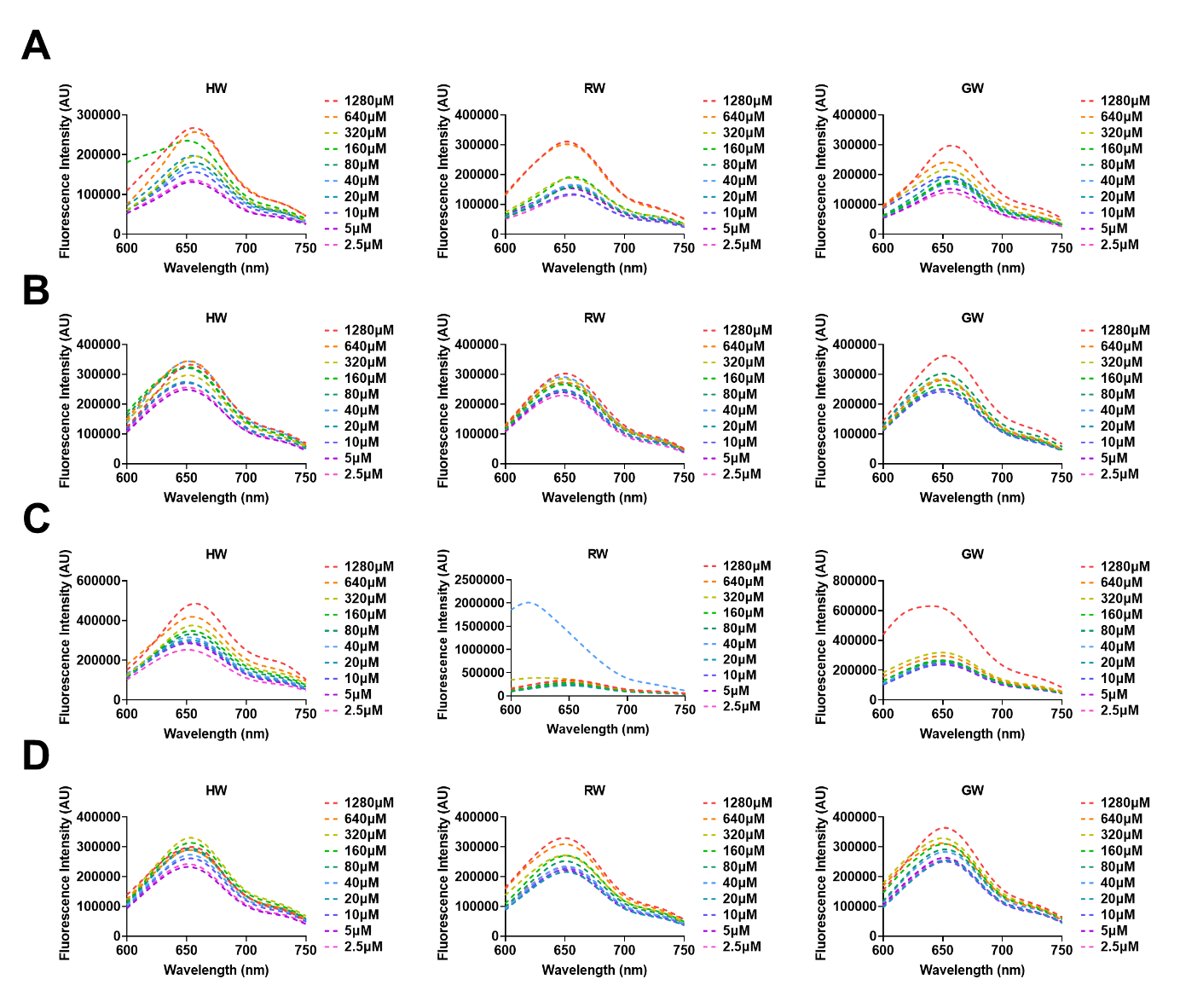


# Figure S7. The fluorescence intensity of BPs was measured by a Nile Red probe at pH values of (A) 7.4, (B) 7.0, (C) 6.5 and (D) 6.0.


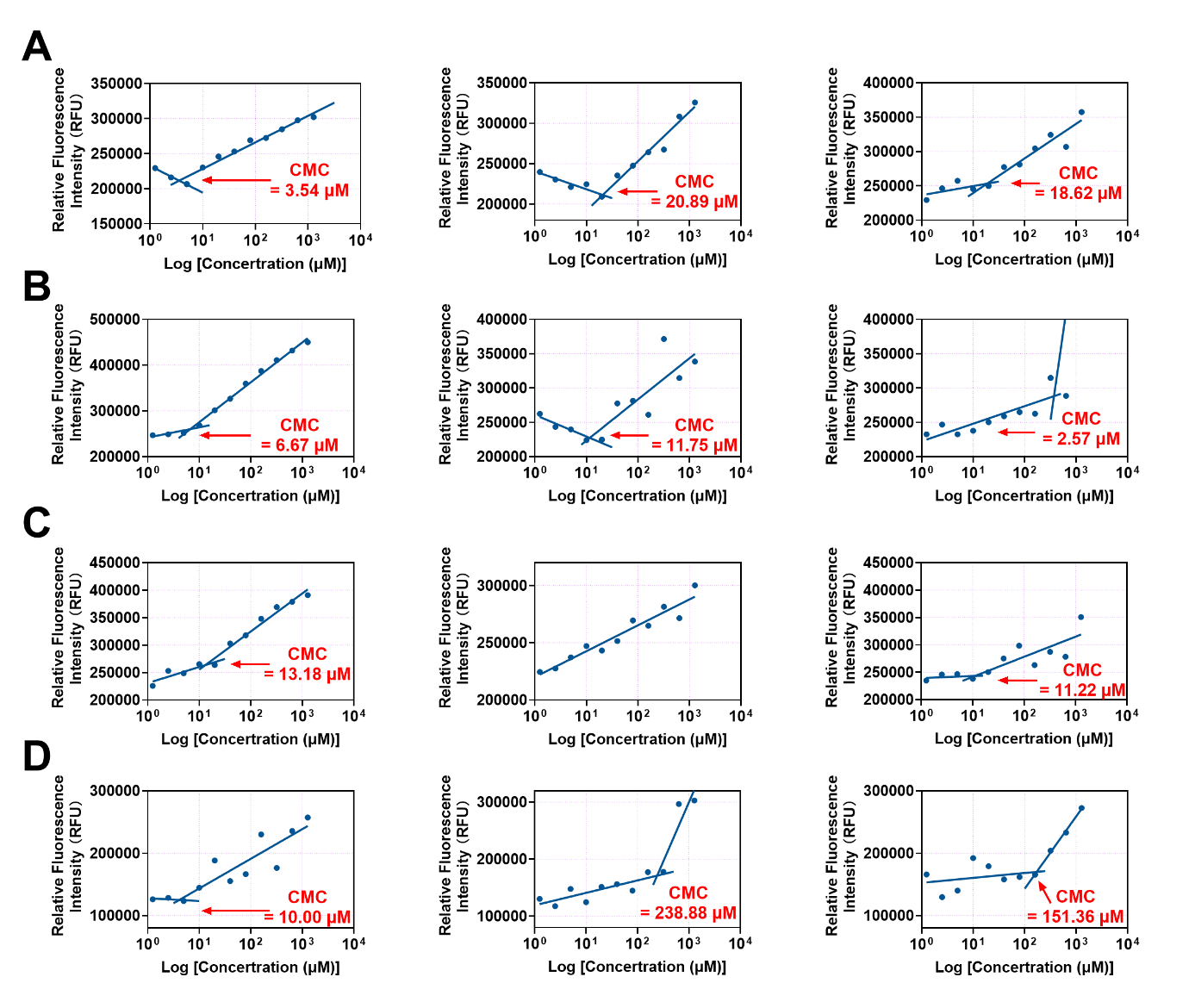


# Figure S8. The critical micelle concentrations of BPs characterized by Nile Red probes were calculated at pH values of (A) 7.4, (B) 7.0, (C) 6.5, and (D) 6.0, respectively.


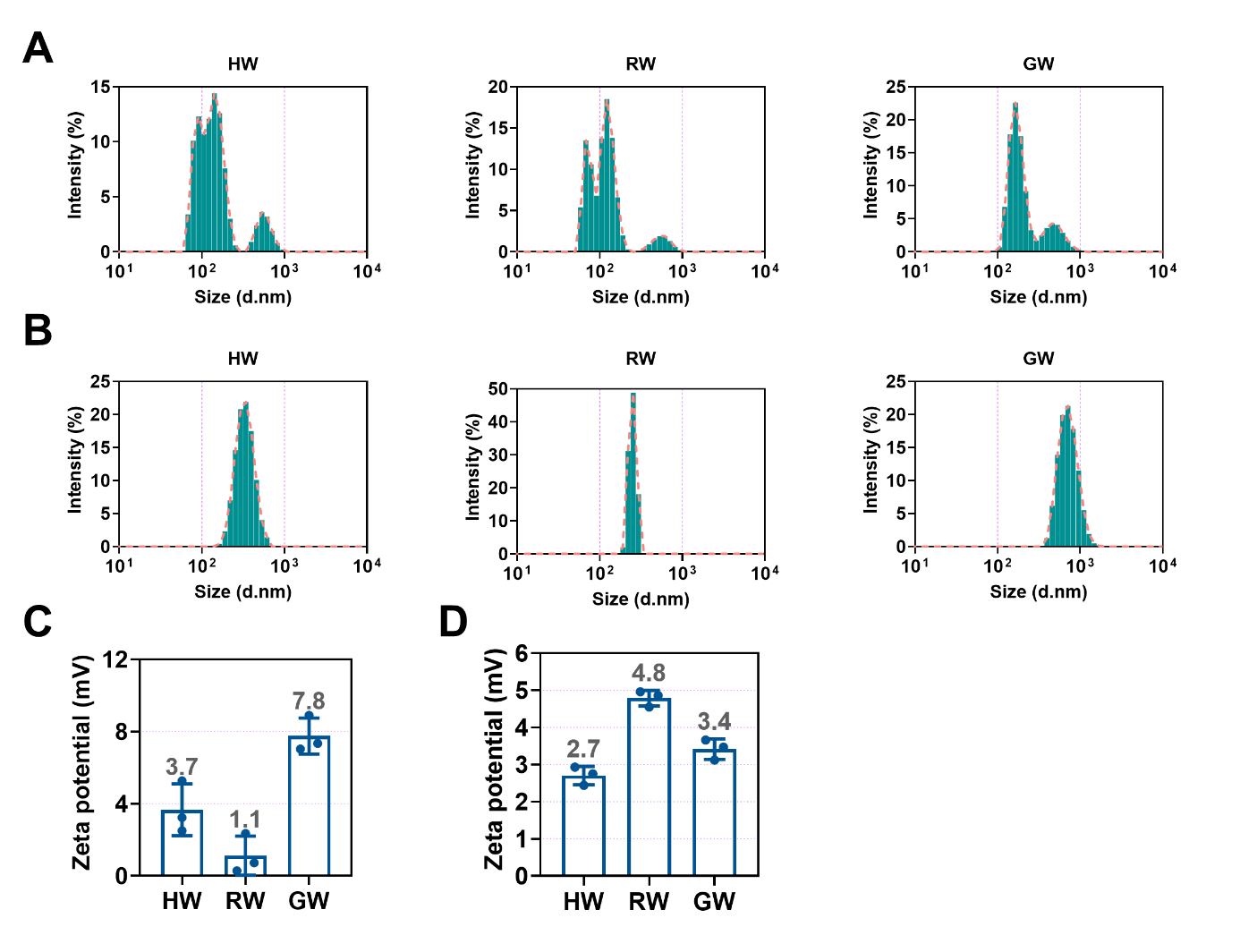


# Figure S9. The hydrodynamic diameter of BPs was determined by DLS method in (A) pH 6.0 and (B) pH 7.4 environment. Zeta potential of BPs was determined by DLS method in (C) pH 6.0 and (D) pH 7.4 environment.


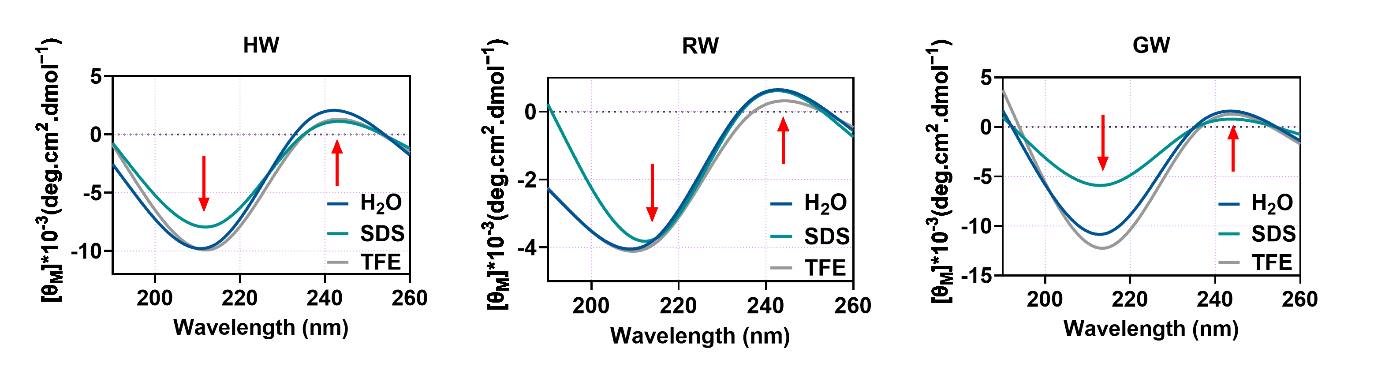


**Figure S10.** Secondary structures of BPs in aqueous solution, 30 mM SDS solution, and 50% TFE solution, monitored by CD; the red arrow highlights the β-sheet signature.


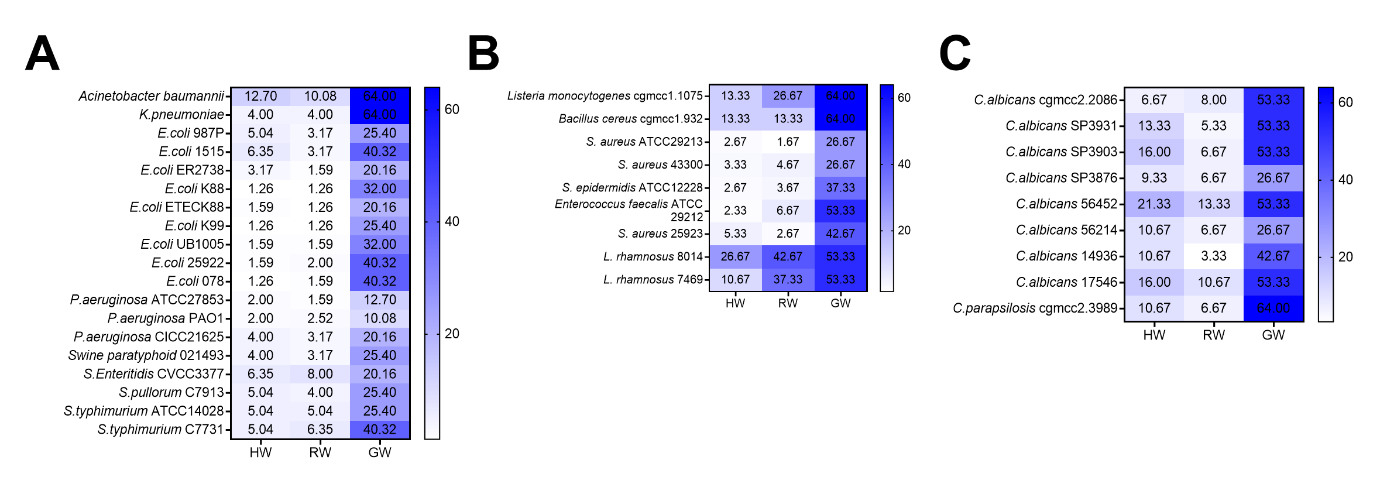


# Figure S11. Minimal inhibitory concentration values of the BPs against (A) gram-negative bacteria, (B) gram-positive bacteria and (C) fungus.

#
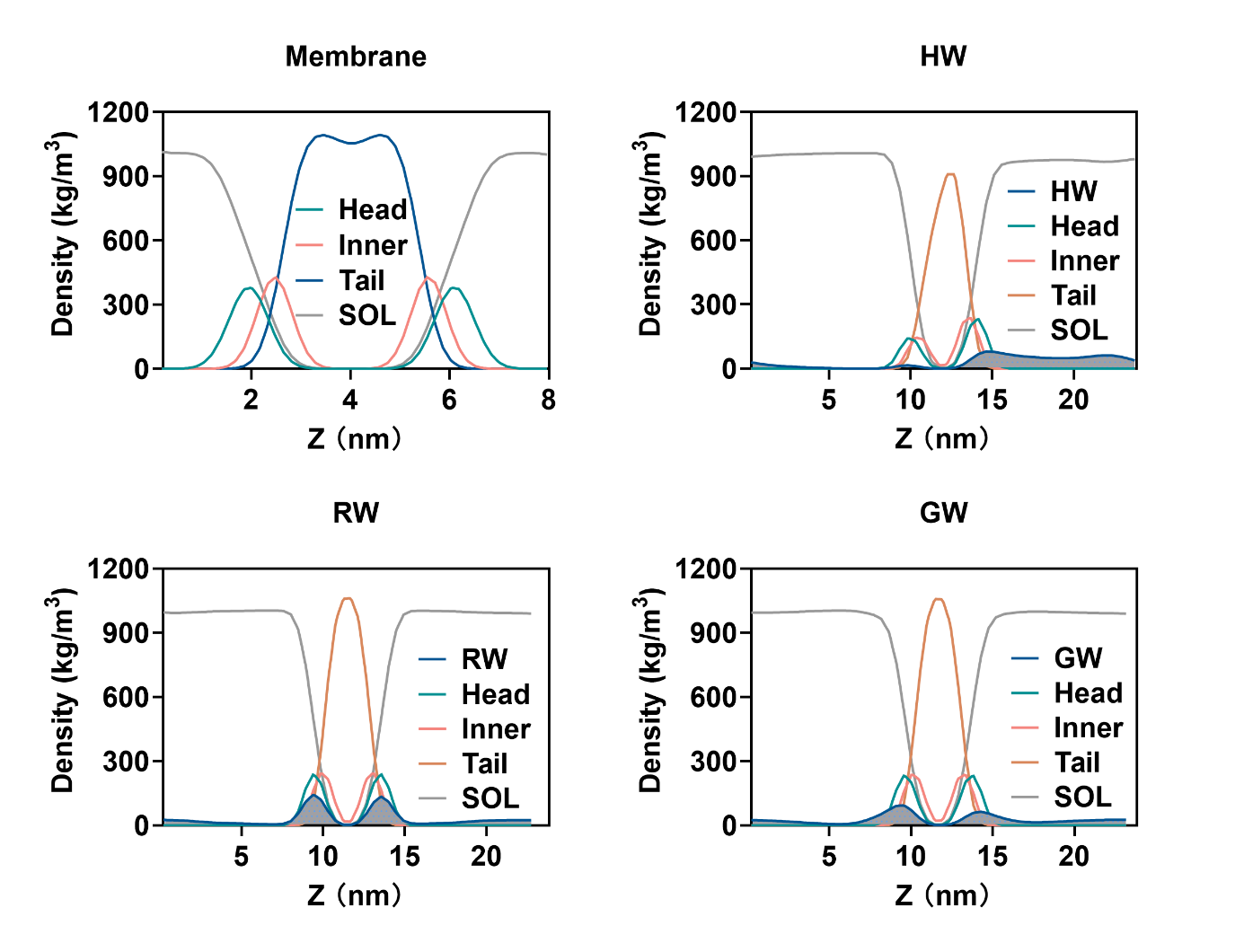


**Figure S12.** Changes in membrane density after BPs interaction with membrane components.

**
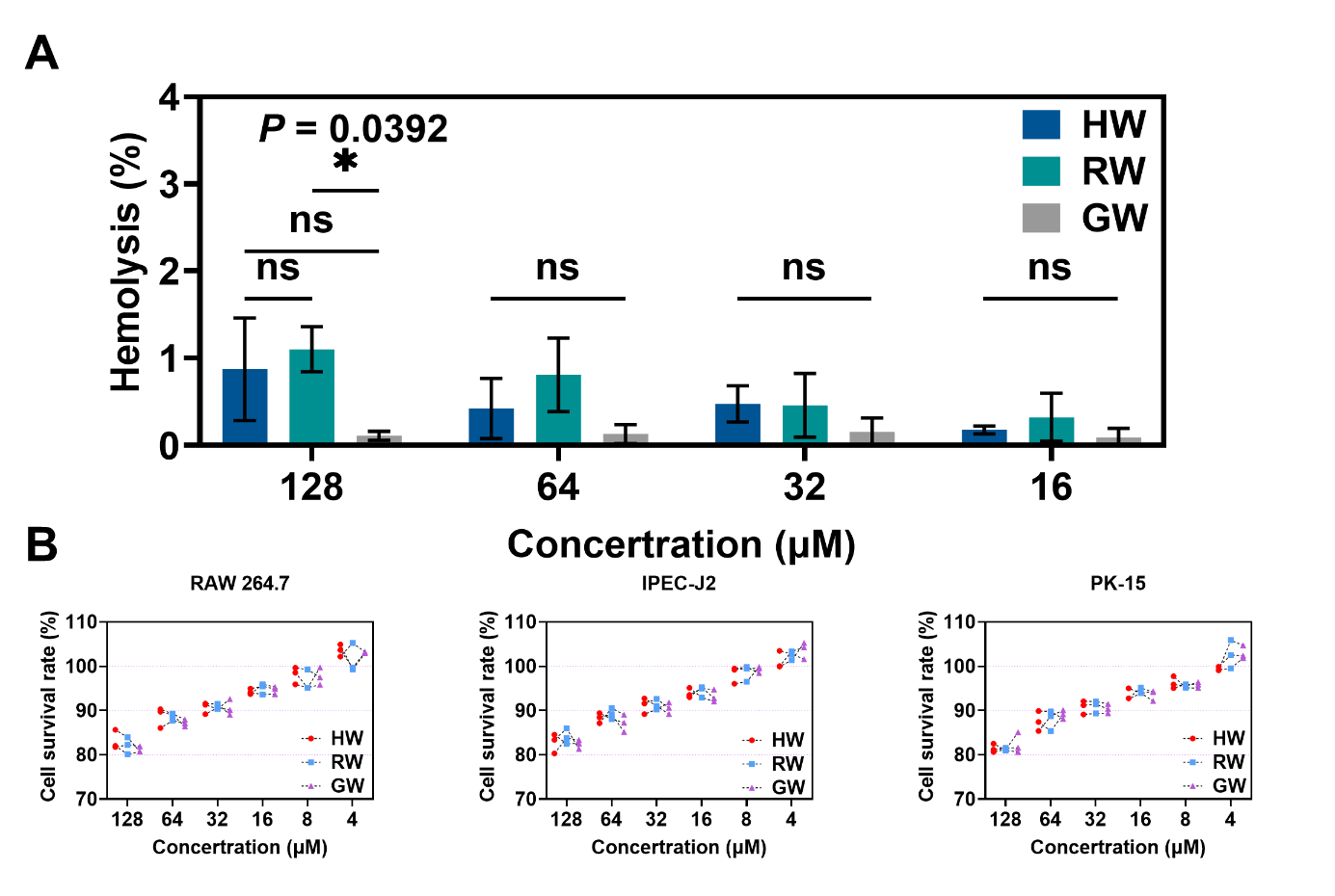
**

# Figure S13. Hemolytic activity of the BPs against human red blood cells (hRBCs). Cytotoxicity of BPs against eukaryotic murine macrophage cells (RAW264.7), intestinal porcine epithelial cell (IPEC-J2) and pig kidney cells (PK-15). Differences between groups exposed to the same concentration were determined by one-way ANOVA followed by Tukey’s post hoc analysis (*n* = 3, * indicates *p* < 0.05).


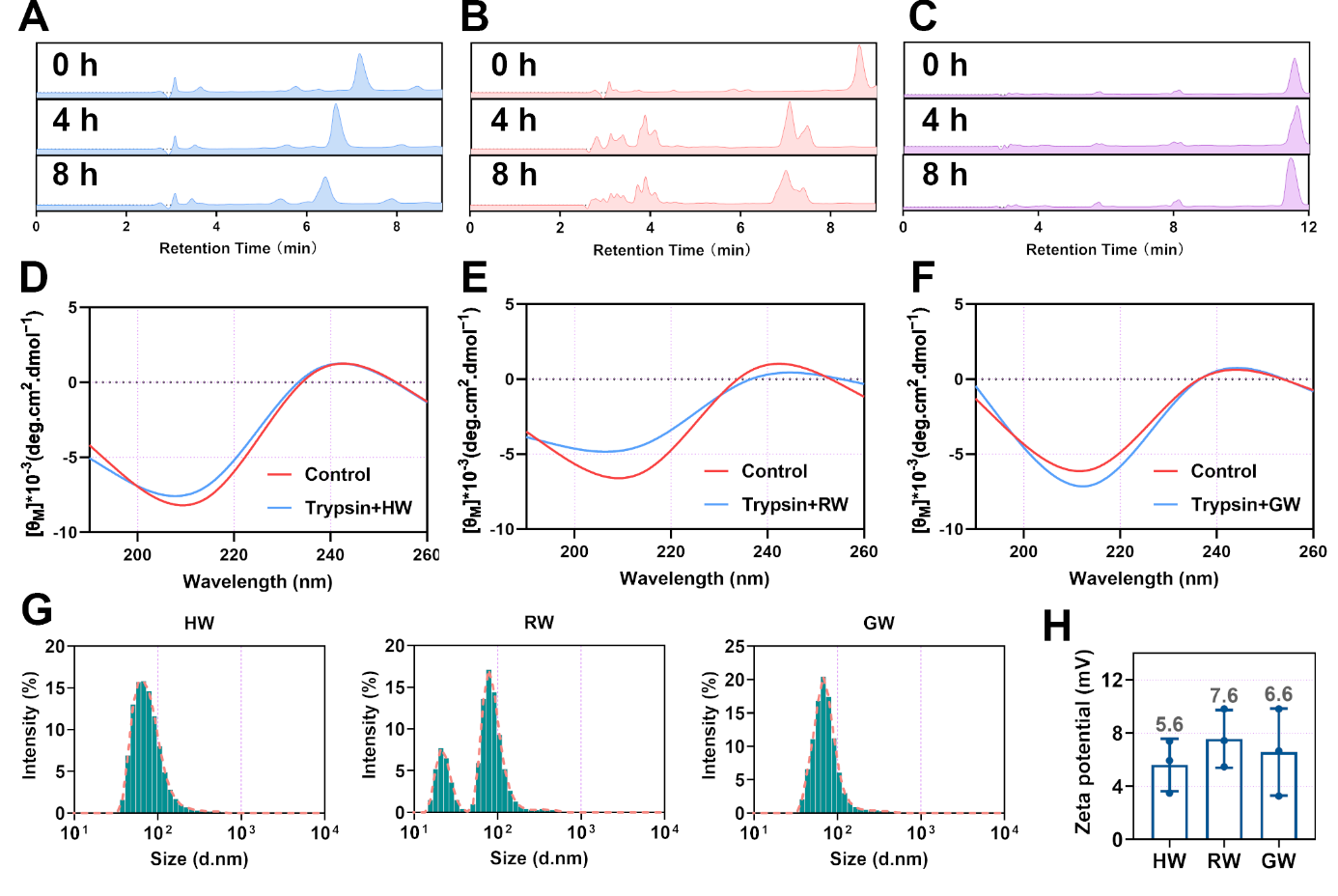


**Figure S14.** The structural fragments of (A) HW, (B) RW, and (C) GW were assessed using HPLC following varying durations of trypsin hydrolysis; The secondary structure changes of (D) HW, (E) RW and (F) GW treated with trypsin (8 mg/ml) for 8 h were detected by circular dichroism chromatography; (G) The hydrodynamic diameter of BPs after trypsin hydrolysis were determined by DLS method; (H) Zeta potential of BPs after trypsin hydrolysis was determined by DLS method.

**
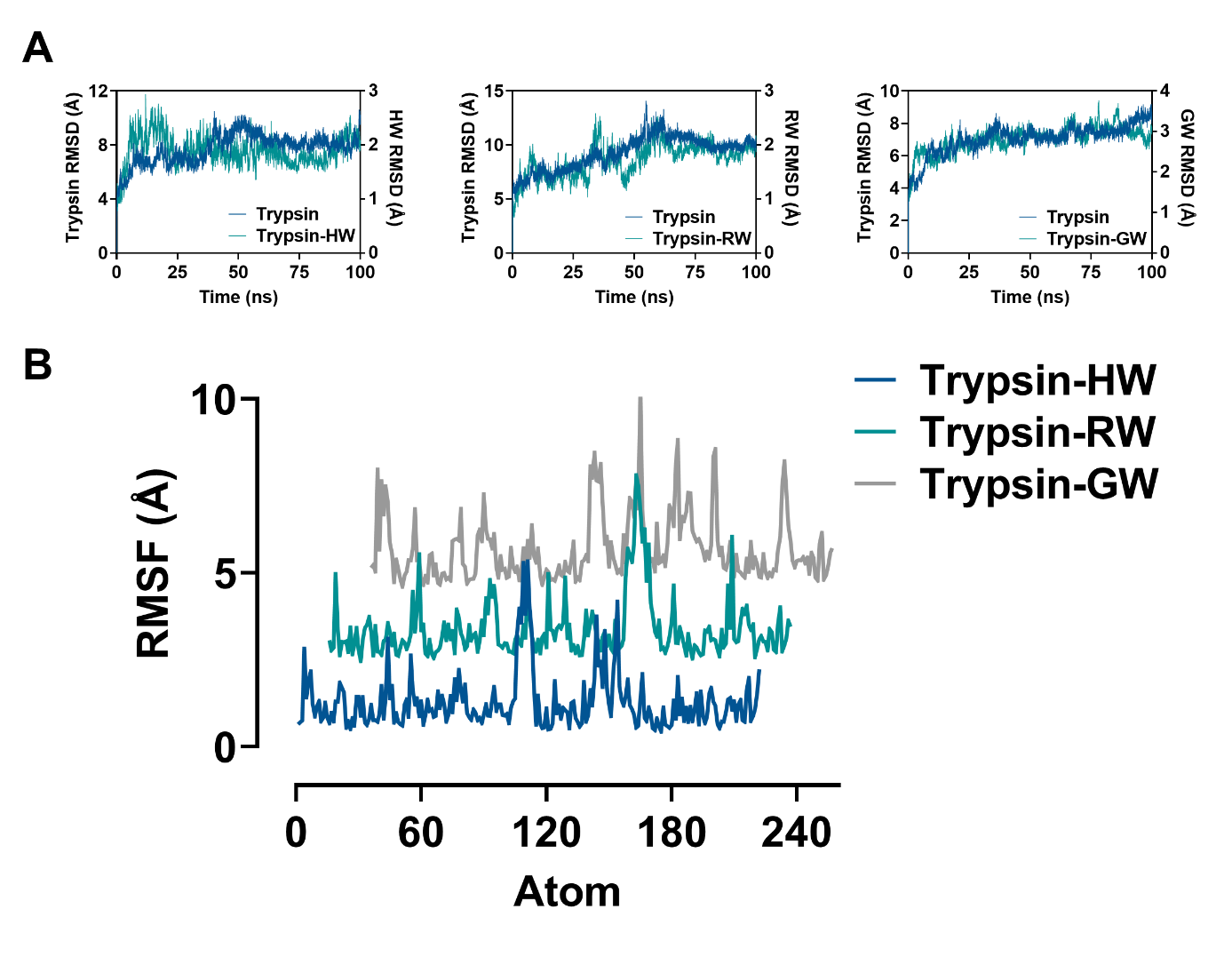
**

# Figure S15. After molecular docking of BPs and trypsin, MD simulations with a duration of 100 ns were performed, and the root mean square deviation (A) and root mean square fluctuation (B) of both complexes were calculated.

#
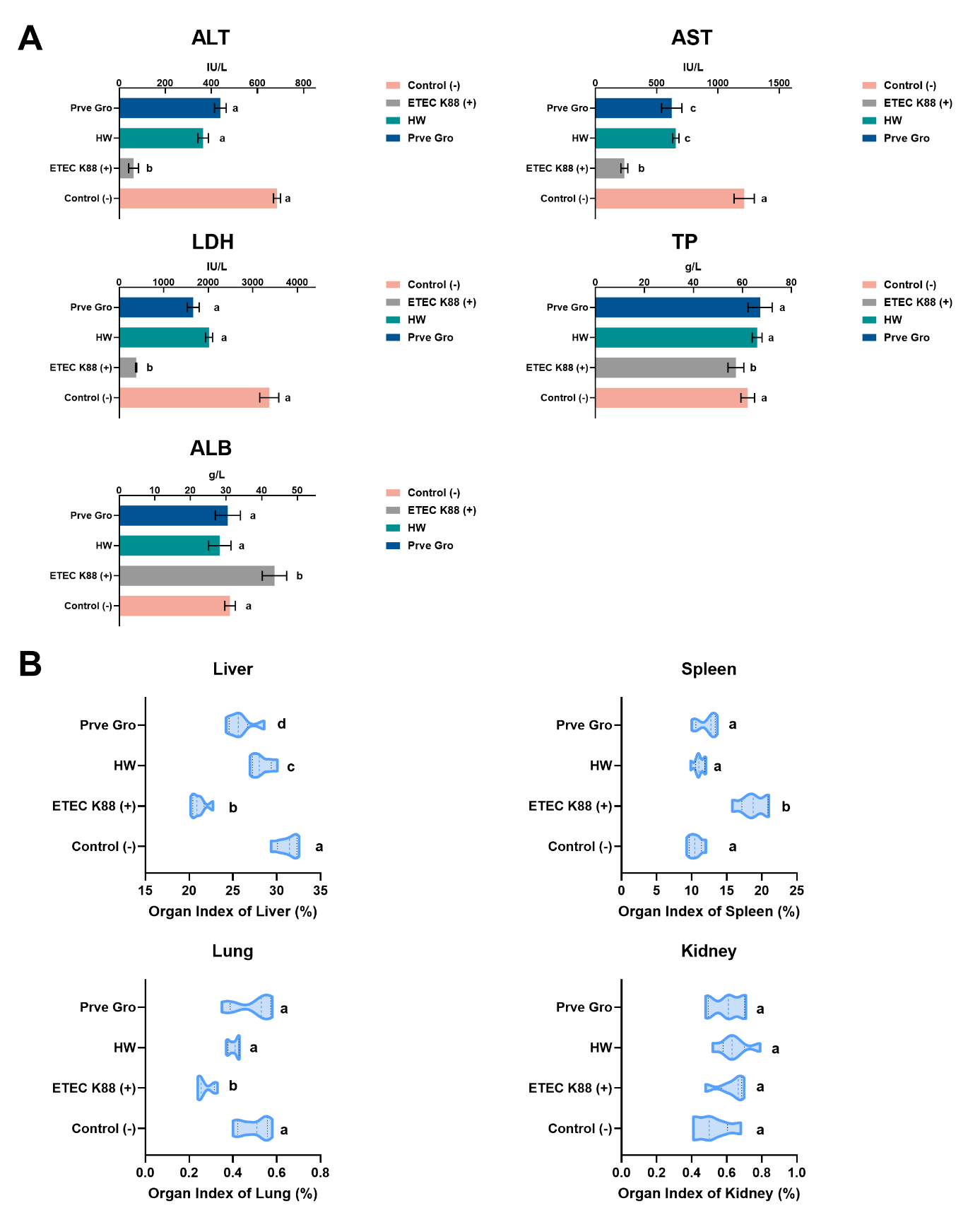


# Figure S16. (A) Hepatic and renal function parameters in C57BL/6 mice serum at 7-day time point. (B) Relative organ weights in C57BL/6 mice at 7-day time point. Data are presented as mean ± s.d.. Statistical differences between groups were determined by one-way ANOVA followed by Tukey’s post hoc test (*n* = 6, different letters indicate significant differences *p* < 0.05).

#
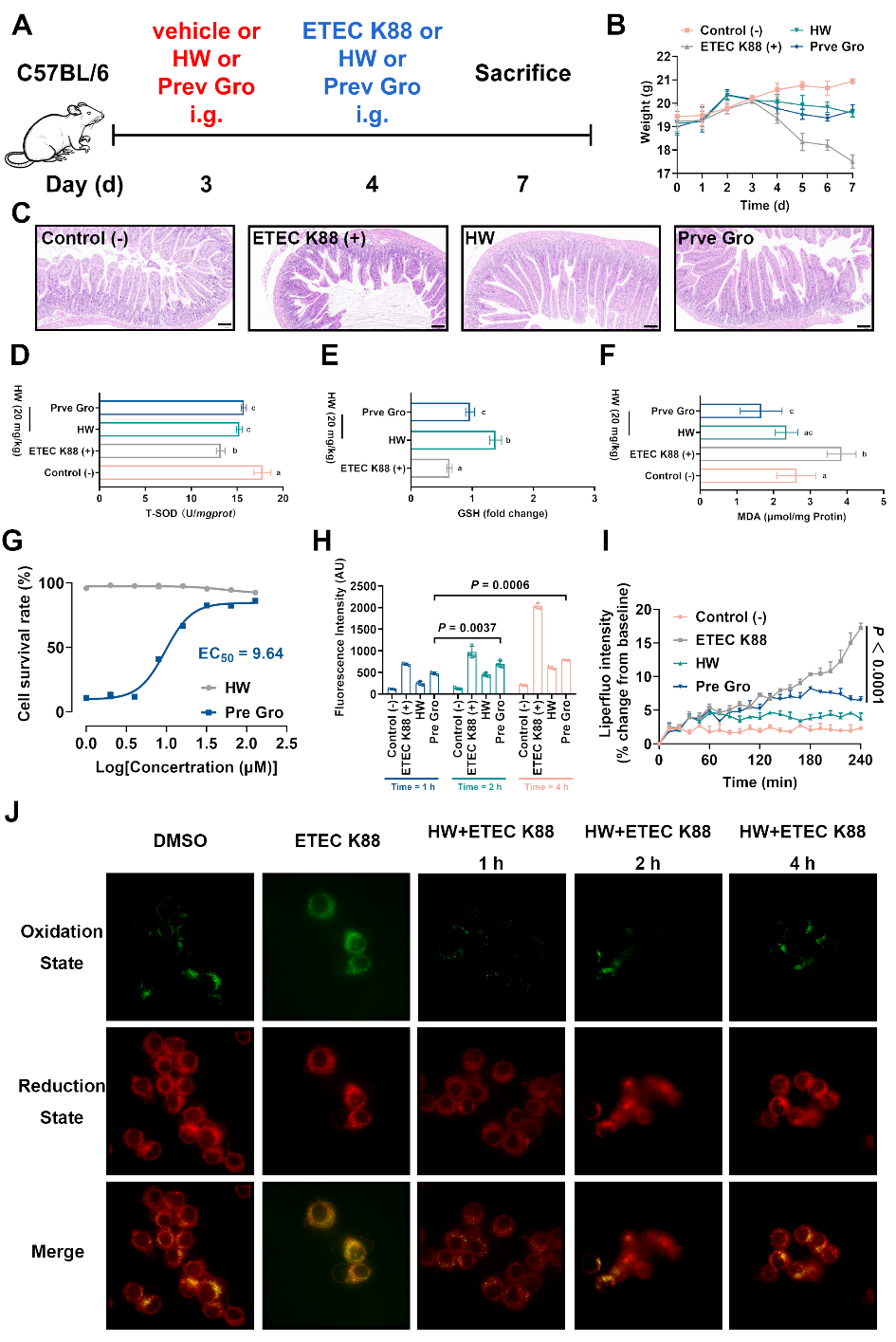


**Figure S17. The lead molecules HW acts as an effective anti-ferroptosis small molecule.** (A) Experimental scheme depicting the induction of intestinal inflammation in mice by ETEC K88. (B) Body weight changes in C57BL/6 mice administered with HW (20 mg kg^−1^) for 7 d. (C) Histopathological evaluation of the jejunum in mice on day 7. Scale bar: 100 µm. (D-F) Measurement of total superoxide dismutase (T-SOD), glutathione (GSH), and malondialdehyde (MDA) activity in jejunal tissue of C57BL/6 mice. *n*=6 (D, E and F). (G) RAW 264.7 cells treated with varying concentrations of HW for 4 h. RAW 264.7 cells pre-exposed to ETEC K88 (OD_600nm_ = 0.4) were incubated with HW for 4 h. (H) Inhibition of ETEC K88-induced ROS production in RAW 264.7 cells by BPs-HW. (I) Detection of lipid peroxides in RAW 264.7 cells treated with BPs-HW using a Liperfluo probe. (J) Super-resolution microscopy of lipid peroxide levels in RAW 264.7 cells, visualized using BODIPY 581/591C11 staining. The ultra-high resolution equipment does not provide any scale output. Statistical differences between groups were determined by one-way ANOVA followed by Tukey’s post hoc test (*n* = 3 for H and I, *n* = 6 for D-F, different letters indicate significant differences *p* < 0.05).

**
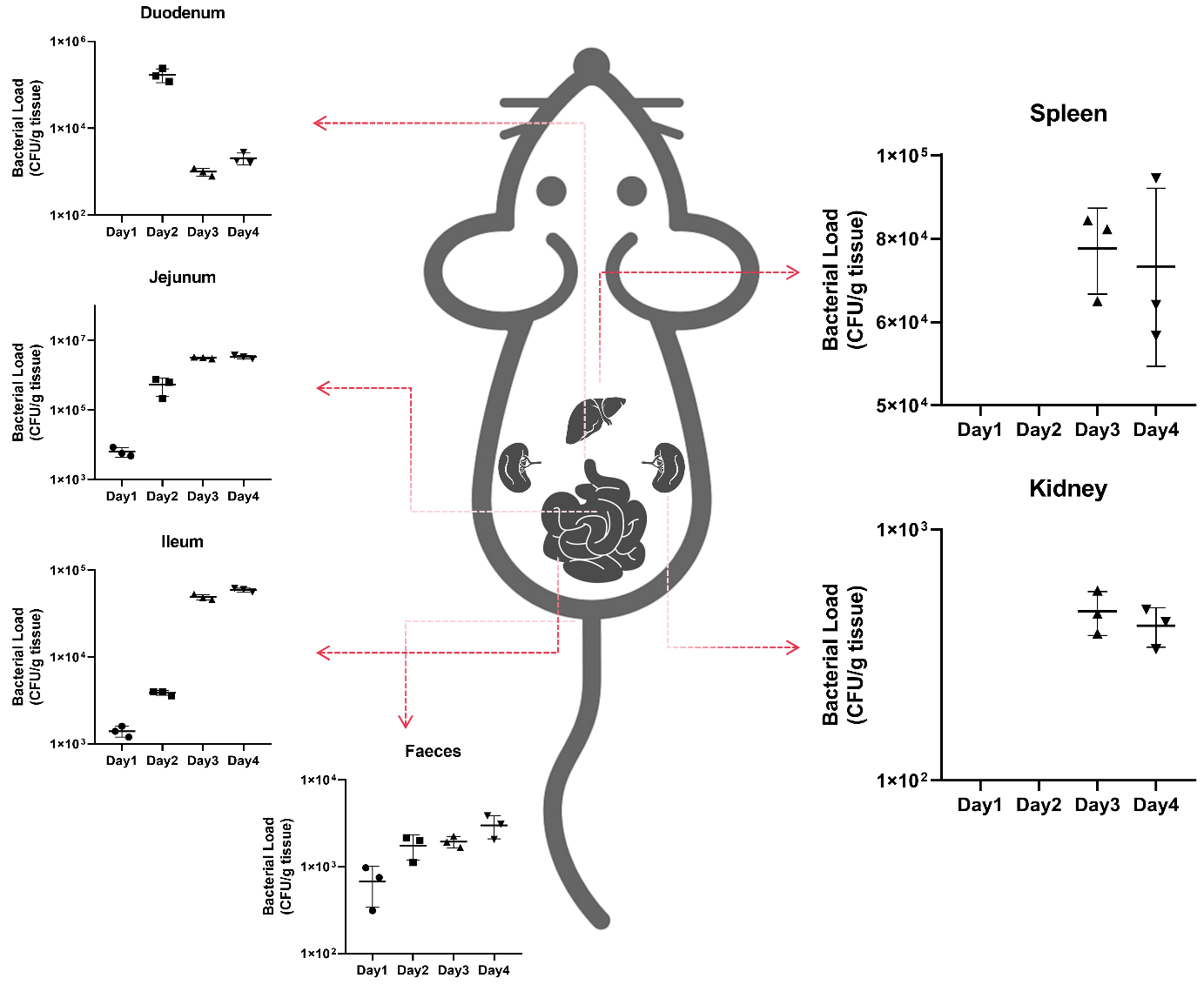
**

# Figure S18. Germ-free C57BL/6 mice were intragastrically administered with ETEC K88 for 4 consecutive days, and the intestinal, fecal and blood microbial contents were monitored.

**
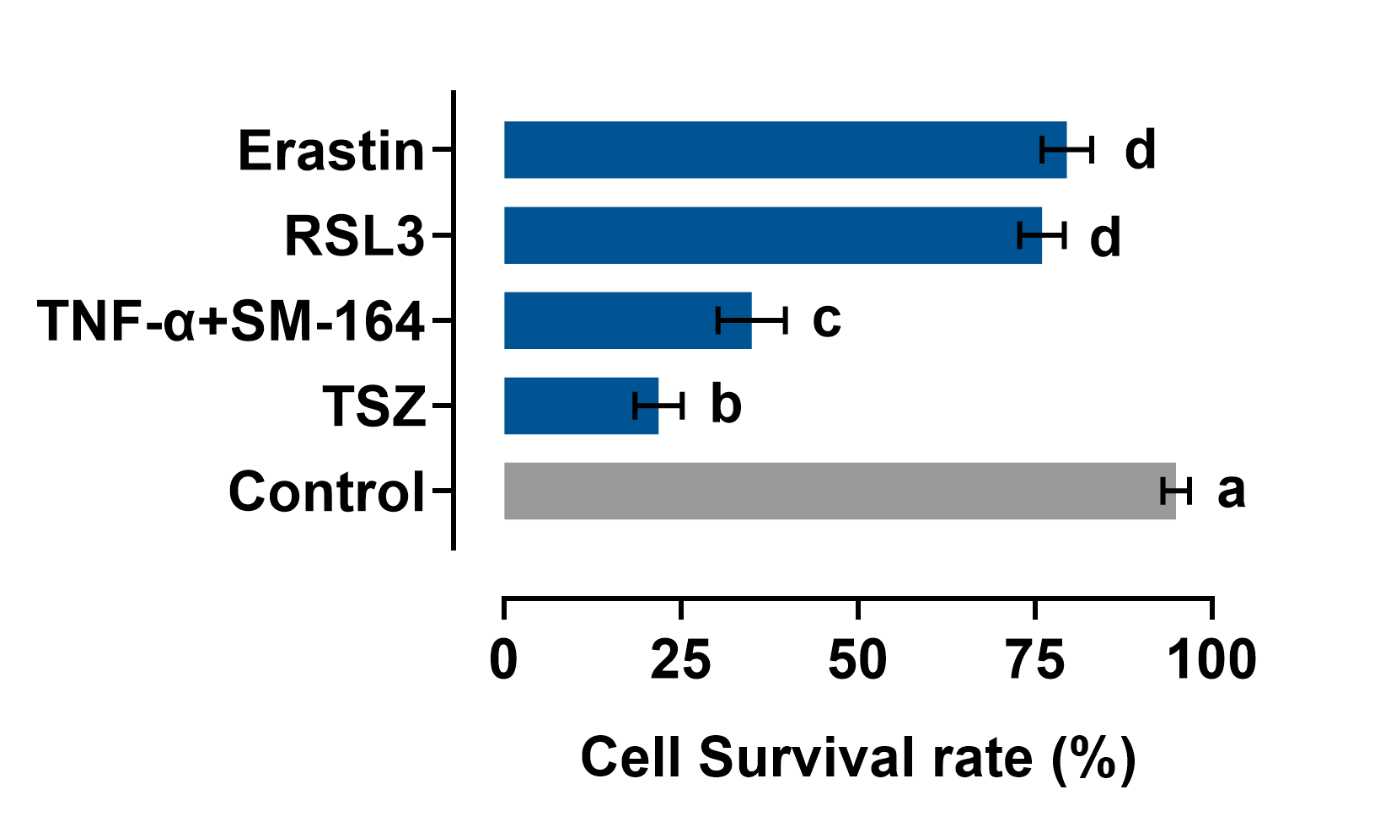
**

# Figure S19. Viability of RAW264.7 cell treated with erastin, RSL3, TNF-α+SM-164, or TSZ. Statistical differences between groups were determined by one-way ANOVA followed by Tukey’s post hoc test (*n* = 3, different letters indicate significant differences *p* < 0.05).

**
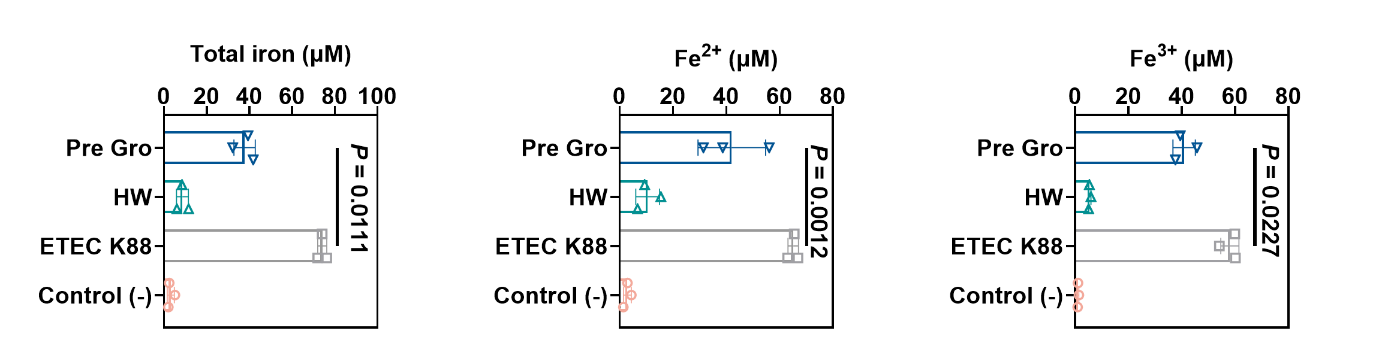
**

# Figure S20. BPs-HW restored the abnormal iron metabolism in RAW264.7 cells stimulated by ETEC K88. Data are presented as mean ± s.d.; Statistical differences between groups were determined by one-way ANOVA followed by Tukey’s post hoc test (*n* = 3).

**
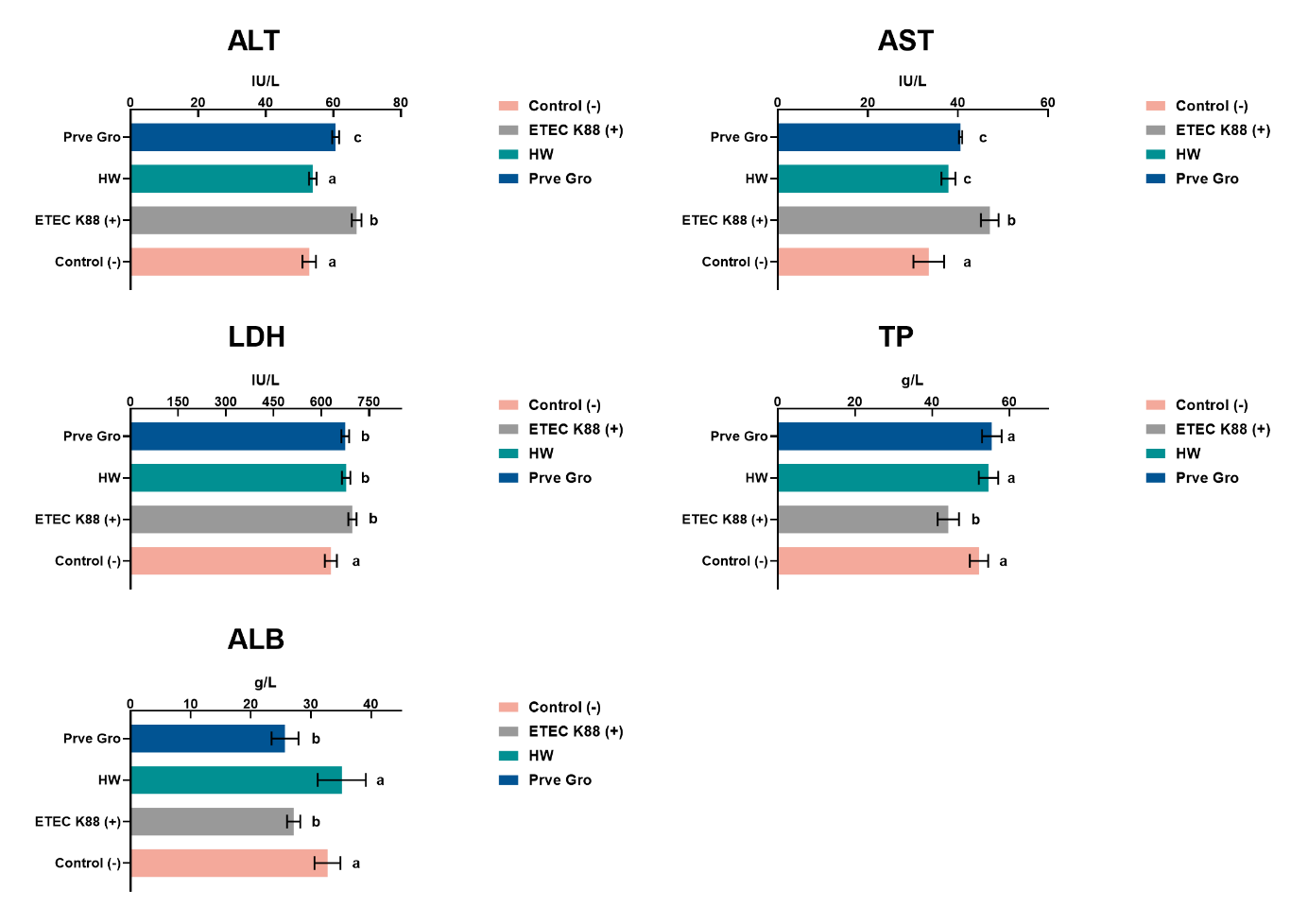
**

# Figure S21. Hepatic and renal function parameters in weaned piglets’ serum at 7-day time point. Data are presented as mean ± s.d.. Statistical differences between groups were determined by one-way ANOVA followed by Tukey’s post hoc test (*n* = 6, different letters indicate significant differences *p* < 0.05).


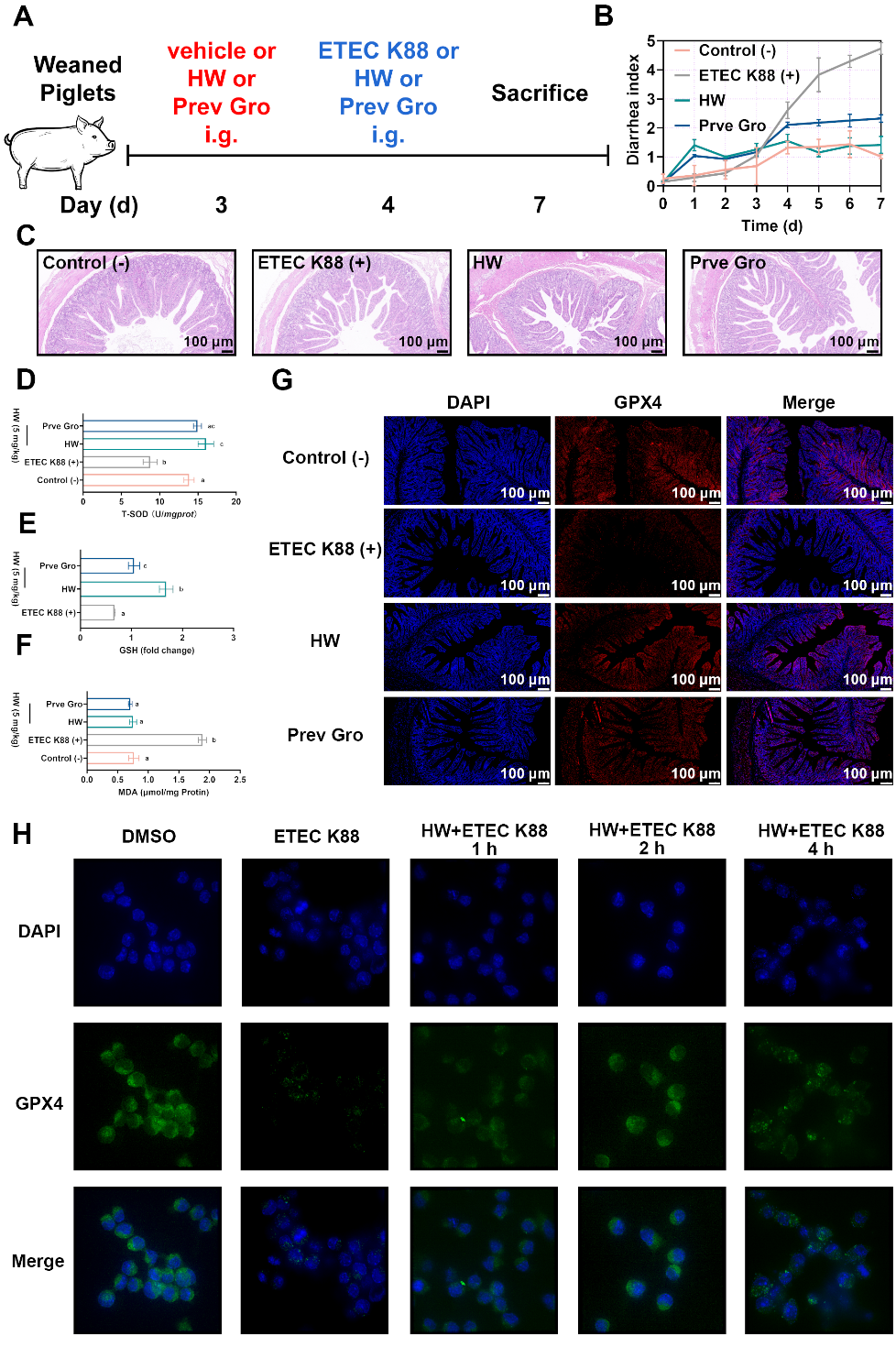


**Figure S22. The lead molecules HW protect against intestinal oxidative stress in weaned piglets by inhibiting ferroptosis.** (A) Experimental model of ETEC K88-induced intestinal inflammation in weaned piglets. (B) Fecal appearance in weaned piglets over 7 d, with corresponding scores assigned. Scores: 1, dry and hard; 2, thick; 3, semi-solid; 4, loose; 5, watery. *n*=6. (C) Histopathological assessment of the jejunum in weaned piglets on day 7. Scale bar: 100 µm. (D-F) Evaluation of T-SOD, GSH, and MDA activity in jejunal tissues of weaned piglets. *n*=6 (B, D, E and F). (G) Representative immunofluorescence images of jejunal tissue in weaned piglets. Red fluorescence indicates GPX4 protein expression. Scale bar: 100 µm. (H) Super-resolution microscopy of GPX4 protein expression in RAW 264.7 cells. The ultra-high resolution equipment does not provide any scale output. Statistical differences between groups were determined by one-way ANOVA followed by Tukey’s post hoc test (*n* = 6, different letters indicate significant differences *p* < 0.05).

**
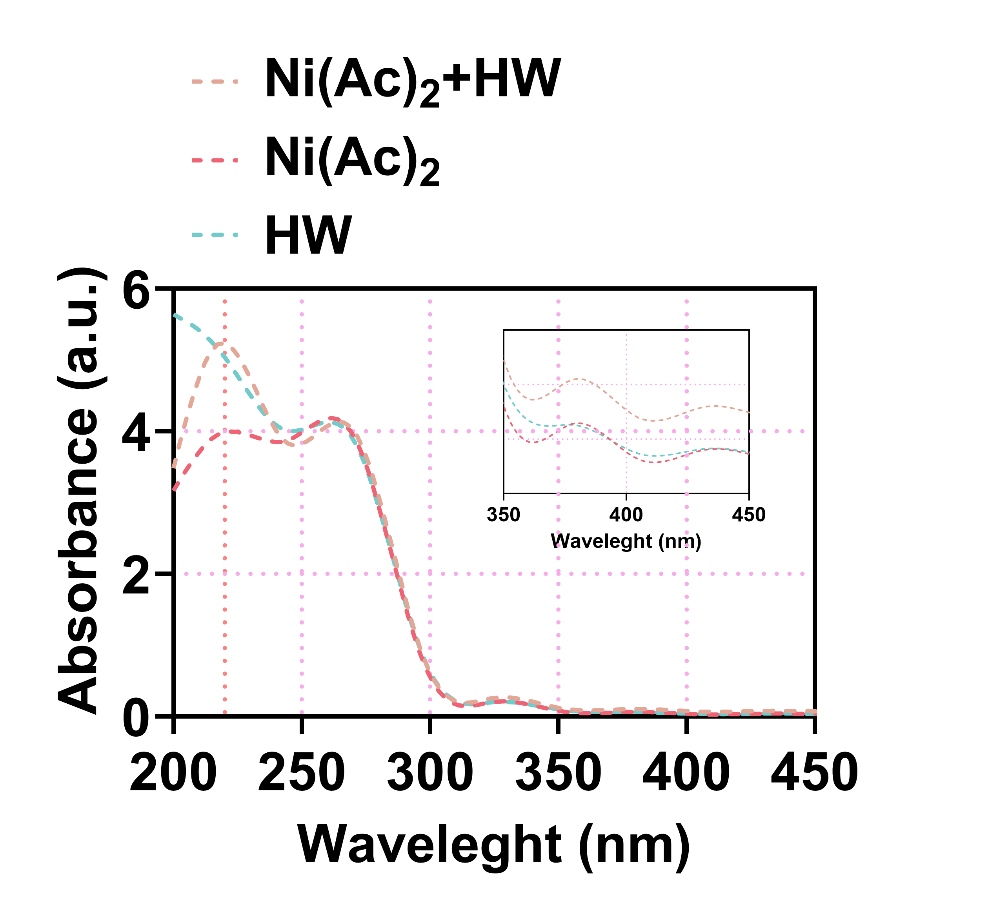
**

**Figure S23**. UV-VIS of Ni-HW from 200-450 nm.

#
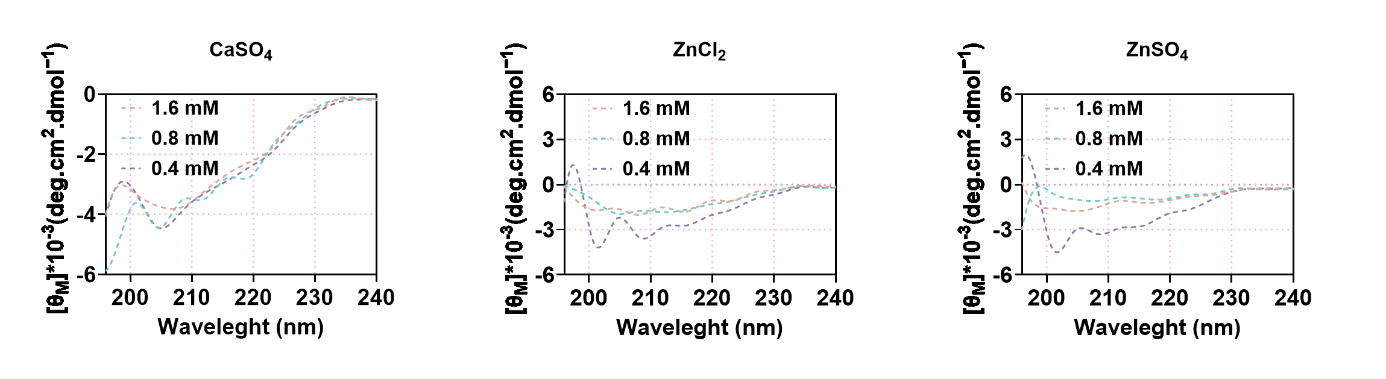


# Figure S24. CD spectra of HW following coordination with various metal acetates.

**
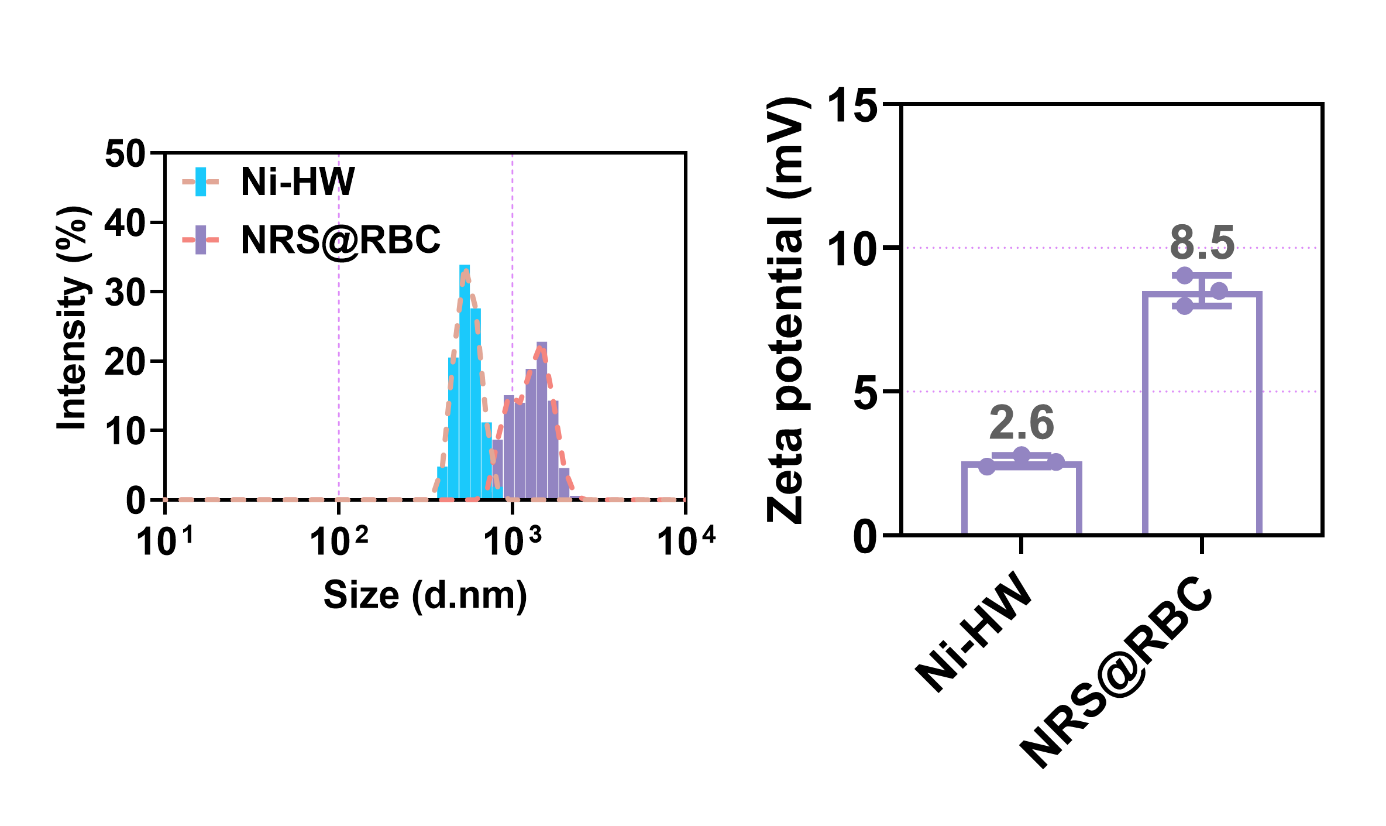
**

# Figure S25. Hydrodynamic diameter and Zeta potential of Ni-HW and NRS@RBC before and after coating with erythrocyte membranes, measured by DLS.

**
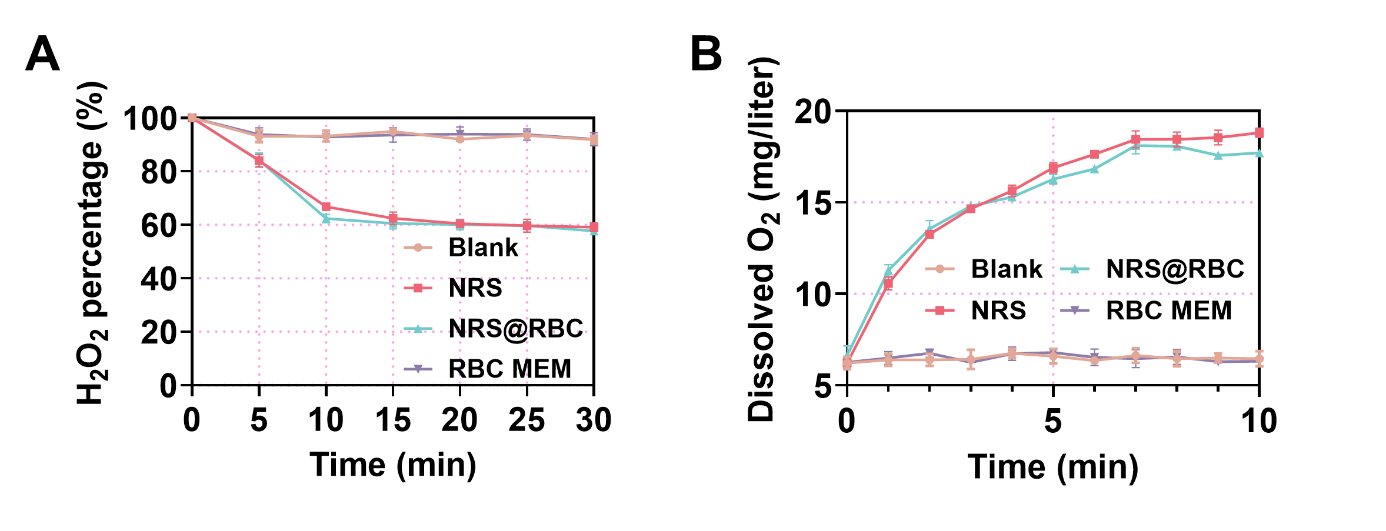
**

# Figure S26. (A) The H_2_O_2_ decomposition profiles in a 10 mM H_2_O_2_ solution in the presence of Blank, NRS, NRS@RBC, and RBC MEM. (B) The O_2_ generation profiles in a 10 millimolar H_2_O_2_ solution under physiological conditions. Statistical differences between groups exposed to the same concentration were analyzed by one-way ANOVA followed by Tukey’s post hoc test (*n* = 3)

**
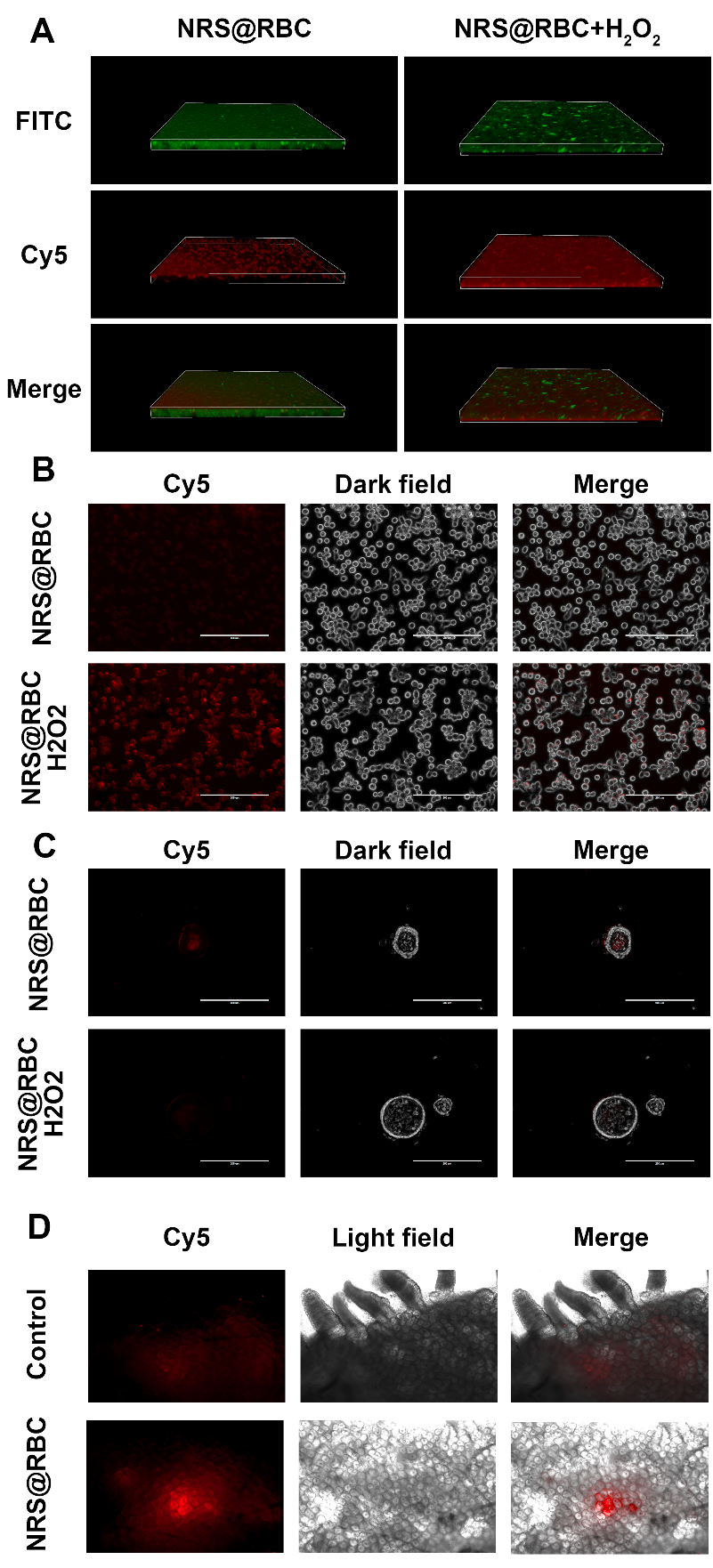
**

# Figure S27. (A) Three-dimensional imaging reveals that drug (indicated in red) penetrate the mucus layer (shown in green); Fluorescence microscope image of (B) IPEC-J2 cells or (C) mouse intestinal crypt-derived organoids in the basolateral chamber, captured 1 h after apical administration of nanoparticles across Transwell membranes coated with a mucus-secreting layer for *in vitro* mucus penetration studies. Scale bar = 200 μm; (D) Fluorescence distribution in colitis tissues after drug administration.

**
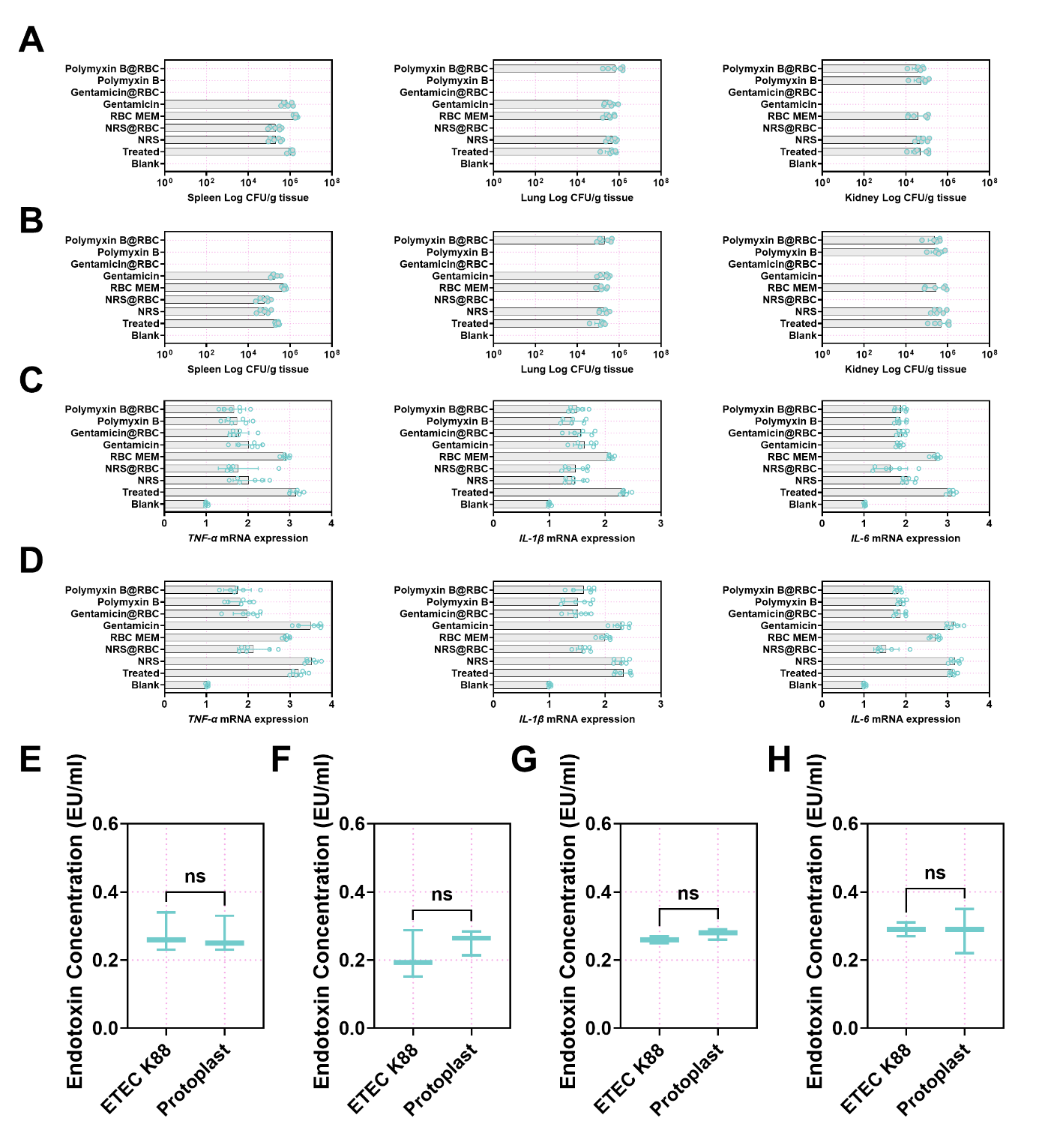
**

# Figure S28. Bacterial loads in spleen, lung and kidney tissues across nine groups of mice during (A) the acute phase and (B) the chronic phase (*n* = 6). The effects of various compounds on the expression levels of inflammatory factors in the colons of mice during (C) the acute phase and (D) the chronic phase; (*n* = 6). The neutralizing effects of (E) NRS, (F) NRS@RBC, (G) polymyxin B, and (H) polymyxin B@RBC against ETEC K88 and protoplast-derived endotoxins; (*n* = 3). Statistical differences between groups exposed to the same concentration were analyzed by one-way ANOVA followed by Tukey’s post hoc test.

**
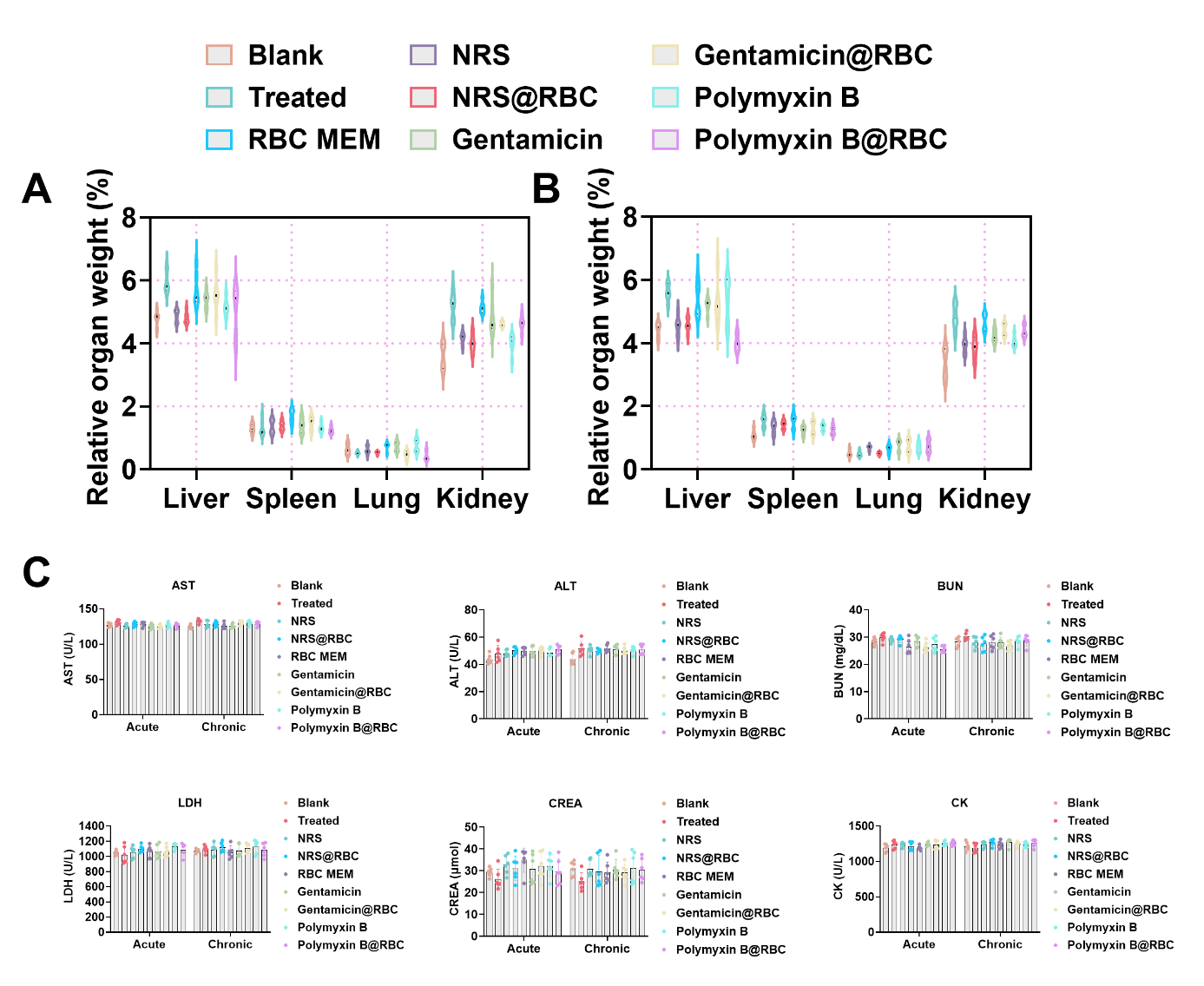
**

# Figure S29. The safety evaluation of various compounds. (A) The blood biochemistry analysis of experimental individuals. (B) Relative organ index of experimental individuals. Statistical differences between groups exposed to the same concentration were analyzed by one-way ANOVA followed by Tukey’s post hoc test.

**
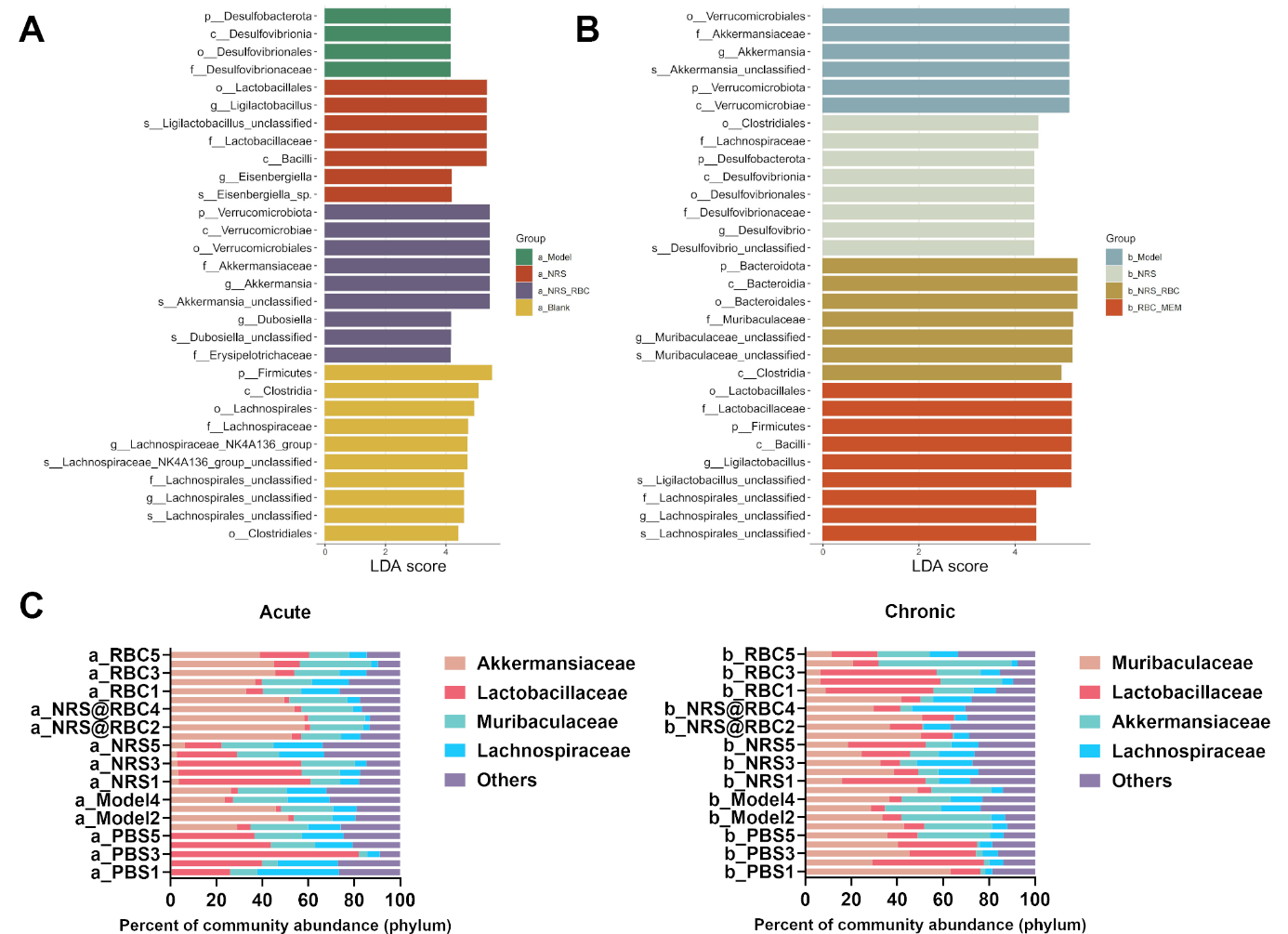
**

**Figure 30.** Linear discriminant analysis effect size (LEf Se) was performed to assess differences across taxonomic levels ranging from phylum to genus. A represents the acute stage, and B represents the chronic stage. (C) Significant changes in the relative abundance of the microbiota at the phylum level.

# Table S1. Physicochemical parameters of BPs.

| **BPs** | **Sequence** | **Theoretical MW** | **Measured MW** | **Retention time [min]** | **Net Charge** | **Hydrophobicity** | **Hydrophobic moment** |
| --- | --- | --- | --- | --- | --- | --- | --- |
| HW | Ac-HHHRPWRPWRPWRPWHHH-NH_2_ | 2639.92 | 2641.25 | 9.413 | +4 | 0.479 | 0.312 |
| RW | Ac-RRRRPWRPWRPWRPWRRR-NH_2_ | 2754.20 | 2754.00 | 12.634 | +10 | 0.099 | 0.329 |
| GW | Ac-GGGRPWRPWRPWRPWGGG-NH_2_ | 2159.38 | 2159.60 | 9.620 | +4 | 0.436 | 0.313 |

# Table S2. Minimum inhibitory concentration (10^-6^ m) of the BPs against gram-negative bacteria.

| **BPs** | **HW** | **RW** | **GW** |
| --- | --- | --- | --- |
| ***Acinetobacter baumannii*** | 13.33±4.62 | 10.67±4.62 | 64.00±0.00 |
| ***K. pneumoniae*** | 4.00±0.00 | 4.00±0.00 | 64.00±0.00 |
| ***E. coli 987P*** | 5.33±2.31 | 3.33±1.15 | 26.67±9.24 |
| ***E. coli 1515*** | 6.67±2.31 | 3.33±1.15 | 42.67±18.48 |
| ***E.coli ER2738*** | 3.33±1.15 | 1.67±0.58 | 21.33±9.24 |
| ***E. coli K88*** | 1.33±0.58 | 1.33±0.58 | 32.00±0.00 |
| ***E. coli ETEC K88*** | 1.67±0.58 | 1.33±0.58 | 21.33±9.24 |
| ***E. coli K99*** | 1.33±0.58 | 1.33±0.58 | 26.67±9.24 |
| ***E. coli UB1005*** | 2.00±1.73 | 1.67±0.58 | 32.00±0.00 |
| ***E. coli 25922*** | 1.67±0.58 | 2.33±1.53 | 42.67±18.48 |
| ***E.coli 078*** | 1.33±0.58 | 2.00±1.73 | 42.67±18.48 |
| ***P. aeruginosa ATCC*27853** | 2.00±0.00 | 2.00±1.73 | 13.33±4.62 |
| ***P. aeruginosa PAO1*** | 2.00±0.00 | 2.67±1.15 | 10.67±4.62 |
| ***P. aeruginosa CICC* 21625** | 5.67±4.04 | 3.33±1.15 | 21.33±9.24 |
| ***Swine paratyphoid* 021493** | 4.67±3.06 | 3.33±1.15 | 26.67±9.24 |
| ***S. Enteritidis CVCC* 3377** | 6.67±2.31 | 9.33±6.11 | 21.33±9.24 |
| ***S. pullorum* C7913** | 5.33±2.31 | 4.67±3.06 | 26.67±9.24 |
| ***S. typhimurium* ATCC 14028** | 5.33±2.31 | 5.33±2.31 | 26.67±9.24 |
| ***S. typhimurium* C7731** | 13.33±4.62 | 10.67±4.62 | 64.00±0.00 |

# Table S3. Minimum inhibitory concentration (10^-6^ m) of the BPs against gram-positive bacteria.

| **BPs** | **HW** | **RW** | **GW** |
| --- | --- | --- | --- |
| ***Listeria monocytogenes cgmcc* 1.1075** | 13.33±4.62 | 26.67±9.24 | 64.00±0.00 |
| ***Bacillus cereus cgmcc* 1.932** | 13.33±4.62 | 13.33±4.62 | 64.00±0.00 |
| ***S. aureus ATCC* 29213** | 2.67±1.15 | 1.67±0.58 | 26.67±9.24 |
| ***S. aureus* 43300** | 3.33±1.15 | 4.67±3.06 | 26.67±9.24 |
| ***S. epidermidis ATCC* 12228** | 2.67±1.15 | 3.67±3.79 | 37.33±24.44 |
| ***Enterococcus faecalis ATCC* 29212** | 2.33±1.53 | 6.67±2.31 | 53.33±18.48 |
| ***S. aureus* 25923** | 5.33±2.31 | 2.67±1.15 | 42.67±18.48 |
| ***L. rhamnosus* 8014** | 26.67±9.24 | 42.67±18.48 | 53.33±18.48 |
| ***L. rhamnosus* 7469** | 10.67±4.62 | 37.33±24.44 | 53.33±18.48 |

# Table S4. Minimum inhibitory concentration (10^-6^ m) of the BPs against fungus.

| **BPs** | **HW** | **RW** | **GW** |
| --- | --- | --- | --- |
| ***C. albicans cgmcc* 2.2086** | 6.67±2.31 | 8.00±0.00 | 53.33±18.48 |
| ***C. albicans* SP3931** | 13.33±4.62 | 5.33±2.31 | 53.33±18.48 |
| ***C. albicans* SP3903** | 16.00±0.00 | 6.67±2.31 | 53.33±18.48 |
| ***C. albicans* SP3876** | 9.33±6.11 | 6.67±2.31 | 26.67±9.24 |
| ***C. albicans* 56452** | 21.33±9.24 | 13.33±4.62 | 53.33±18.48 |
| ***C. albicans* 56214** | 10.67±4.62 | 6.67±2.31 | 26.67±9.24 |
| ***C. albicans* 14936** | 10.67±4.62 | 3.33±1.15 | 42.67±18.48 |
| ***C. albicans* 17546** | 16.00±0.00 | 10.67±4.62 | 53.33±18.48 |
| ***C. parapsilosis cgmcc* 2.3989** | 10.67±4.62 | 6.67±2.31 | 64.00±0.00 |

# Table S5. Minimum inhibitory concentration (10^-6^ m) of BPs against ETEC K88 in the presence of physiological salts.

| **BPs** | **Control** | **Na^+^** | **K^+^** | **Ca^2+^** | **Mg^2+^** | **Zn^2+^** | **Fe^3+^** | **NH^4+^** |
| --- | --- | --- | --- | --- | --- | --- | --- | --- |
| **HW** | 2 | 32 | 1 | 16 | 8 | 1 | 1 | 1 |
| **RW** | 2 | 16 | 2 | 16 | 8 | 2 | 2 | 1 |
| **GW** | 16 | 64 | 8 | 64 | 32 | 8 | 8 | 4 |

# Table S6. Stability analysis of the BPs (10^-6^ m) under different conditions.

| **BPs** | **Control** | **Serum Ratio**^a)^ | | **pH Level**^b)^ | | **Temperature**^c)^ | |
| --- | --- | --- | --- | --- | --- | --- | --- |
|  |  | **50 %** | **25 %** | **pH 2** | **pH 12** | **100 ℃** | **80 ℃** |
| HW | 2 | 1 | 2 | 2 | 4 | 2 | 2 |
| RW | 2 | 4 | 4 | 8 | 4 | 4 | 2 |
| GW | 16 | 8 | 8 | 8 | 16 | 16 | 16 |

a: MIC detection was performed after incubating BPs with fetal bovine serum at ratios of 1:1 and 1:3, respectively, at 37°C for 8 h;

b: BPs powder was dissolved in PBS solution (sterilized by filtration through a 0.22 μm membrane), and the pH of the resulting solution was adjusted to 2 and 12 using HCl and NaOH, respectively. MIC detection was subsequently conducted following incubation at 37°C for 8 h;

c: Appropriate quantities of BPs were incubated in water baths at 80°C and 100°C for 30 min, respectively. After cooling to room temperature, the samples were briefly centrifuged, and the supernatant was collected for MIC detection.

# Table S7. The BPs was subjected to hydrolysis by trypsin, chymotrypsin, pepsin, protease K, and papain at a concentration of 8 mg/ml, demonstrating sustained high antimicrobial activity against ETEC K88.

| BPs | | HW | RW | GW |
| --- | --- | --- | --- | --- |
| Control | | 1.33±0.58 | 1.33±0.58 | 16.00±0.00 |
| Trypsin (8 mg/ml) | 1 h | 1.67±0.58 | 5.33±2.31 | 13.33±4.62 |
|  | 2 h | 1.33±0.58 | 6.67±2.31 | 21.33±9.24 |
|  | 4 h | 1.33±0.58 | 21.33±9.24 | 16.00±0.00 |
|  | 8 h | 1.67±0.58 | 42.67±18.48 | 13.33±4.62 |
| Chymotrypsin (8 mg/ml) | 1 h | 1.33±0.58 | 1.67±0.58 | 21.33±9.24 |
|  | 2 h | 1.67±0.58 | 1.67±0.58 | 21.33±9.24 |
|  | 4 h | 1.00±0.00 | 3.33±1.15 | 16.00±0.00 |
|  | 8 h | 1.33±0.58 | 2.67±1.15 | 18.67±12.22 |
| Pepsase (8 mg/ml) | 1 h | 1.33±0.58 | 1.33±0.58 | 18.67±12.22 |
|  | 2 h | 1.00±0.00 | 1.67±0.58 | 16.00±0.00 |
|  | 4 h | 1.33±0.58 | 2.67±1.15 | 16.00±0.00 |
|  | 8 h | 1.00±0.00 | 2.00±0.00 | 13.33±4.62 |
| Proteinase K (8 mg/ml) | 1 h | 1.17±0.76 | 1.33±0.58 | 13.33±4.62 |
|  | 2 h | 1.67±0.58 | 1.67±0.58 | 21.33±9.24 |
|  | 4 h | 1.33±0.58 | 1.33±0.58 | 16.00±0.00 |
|  | 8 h | 1.67±0.58 | 2.33±1.53 | 13.33±4.62 |
| Papain (8 mg/ml) | 1 h | 1.00±0.00 | 3.00±1.73 | 16.00±0.00 |
|  | 2 h | 1.33±0.58 | 1.33±0.58 | 16.00±0.00 |
|  | 4 h | 1.33±0.58 | 1.33±0.58 | 21.33±9.24 |
|  | 8 h | 0.83±0.29 | 1.33±0.58 | 21.33±9.24 |

#

# Table S8. Effects of protease on the antimicrobial activity of the BPs.

| **BPs** | **Trypsin** | **Chymotrypsin** | **Pepsase** | **Proteinase K** | **Papain** |
| --- | --- | --- | --- | --- | --- |
| HW | 0.6808 | 1.0000 | 0.6742 | 0.7591 | 0.6054 |
| RW | 0.0061 | 0.0695 | 0.2199 | 0.4655 | 0.4045 |
| GW | 1.0000 | 0.1753 | 1.0000 | 1.0000 | 0.1661 |

There were significant differences between protease treatment group and control group. A two-tailed Student’s *t*-test was employed for pairwise group comparisons.

# Table S9. The MM-GBSA binding free energy of BPs and trypsin.

| **BPs** | **Protein** | **MM-GBSA dG Bind (kcal/mol)** |
| --- | --- | --- |
| HW | Trypsin | 8.55 |
| RW | Trypsin | -62.43 |
| GW | Trypsin | -17.27 |

When the value is lower than -30 kcal/mol in MM-GBSA analysis, it indicates that the binding free energy is low and the binding between ligand and protein is stable.

# Table S10. BPs and trypsin bind free energy at the last 1ns of MD simulation.

| **BPs** | **Protein** | **MM-GBSA dG Bind (kcal/mol)** |
| --- | --- | --- |
| HW | Trypsin | -28.28 |
| RW | Trypsin | -132.63 |
| GW | Trypsin | -97.50 |

When the value is lower than -30 kcal/mol in MM-GBSA analysis, it indicates that the binding free energy is low and the binding between ligand and protein is stable.

# Table S11. BPs binding site to trypsin.

| **BPs** | **Hydrogen Bond** | **Salt Bridges** | **π-π Stacking** |
| --- | --- | --- | --- |
| **HW** | Arg-Cys*2 | Arg-Cys*3 | Trp-Trp |
|  | Trp-Tyr |  |  |
|  | Arg-Gln |  |  |
| **RW** | Arg-Asp*2 | Arg-Asp  Arg-Cys*4 | Trp-Tyr*2 |
|  | Pro-Ser |  |  |
|  | Arg-Phe |  |  |
|  | Arg-Cys*3 |  |  |
|  | Pro-Gln |  |  |
|  | Arg-Gln |  |  |
|  | Arg-Ser |  |  |
| **GW** | Gly-Asn | Arg-Cys*2 | Trp-Trp |
|  | Arg-Asn*2 |  |  |
|  | Arg-Lys*2 |  |  |
|  | Arg-Cys*4 |  |  |

**Table S12.** CD data of HW following coordination with various metal acetates.

| **Metal salt content (mM)** | **α-helix content [%]** | | | |
| --- | --- | --- | --- | --- |
|  | **Ni^2+^** | **Cu^2+^** | **Mn^2+^** | **Zn^2+^** |
| 1.6 | 20.7 | 2.2 | 13.6 | 1.8 |
| 0.8 | 22.1 | 0.2 | 6.9 | 4.5 |
| 0.4 | 22.6 | 2.1 | 8.3 | 4.7 |
